# Immobilization of Polyoxometalate in the Metal-Organic Framework rht-MOF-1: Towards a Highly Effective Heterogeneous Catalyst and Dye Scavenger

**DOI:** 10.1038/srep25595

**Published:** 2016-05-09

**Authors:** Jing-Wen Sun, Peng-Fei Yan, Guang-Hui An, Jing-Quan Sha, Guang-Ming Li, Guo-Yu Yang

**Affiliations:** 1 Key Laboratory of Functional Inorganic Material Chemistry Ministry of Education of the People’s Republic of China, Heilongjiang University, Harbin, 150080, China; 2 School of Pharmacy, Jiamusi University, Jiamusi, 154007, China; 3 Key Laboratory of Cluster Science Ministry of Education of the People’s Republic of China, Beijing Institute of Technology, Beijing, 100081, China

## Abstract

A series of three remarkable complexes [PMo_12_O_40_]@[Cu_6_O(TZI)_3_(H_2_O)_9_]_4_·OH·31H_2_O (H_3_TZI = 5-tetrazolylisophthalic acid; denoted as HLJU-1, HLJU = Heilongjiang University), [SiMo_12_O_40_]@[Cu_6_O(TZI)_3_(H_2_O)_9_]_4_·32H_2_O (denoted as HLJU-2), and [PW_12_O_40_]@[Cu_6_O(TZI)_3_(H_2_O)_6_]_4_·OH·31H_2_O (denoted as HLJU-3) have been isolated by using simple one-step solvothermal reaction of copper chloride, 5-tetrazolylisophthalic acid (H_3_TZI), and various Keggin-type polyoxometalates (POMs), respectively. Crystal analysis of HLJU 1−3 reveals that Keggin-type polyoxoanions have been fitted snuggly in the cages of rht-MOF-1 (MOF: metal−organic framework) with large cell volume in a range of 87968−88800 Å^3^ and large pore volume of about 68%. HLJU 1–3 exhibit unique catalytic selectivity and reactivity in the oxidation of alkylbenzene with environmental benign oxidant under mild condition in aqueous phase as well as the uptake capacity towards organic pollutants in aqueous solution.

Polyoxometalates (POMs), as a species of significant metal oxide clusters with high negative charge and abundant topologies, have been employed in many research fields, such as catalysis, optics, magnetism, and biological medicine^1^,^2^,^3^,^4^,^5^,^6^,^7^,^8^,^9^,^10^. Particularly, POMs-catalyzed oxidation reactions are garnering increasing attention although it is limited by their low specific surface area and low stability^11^. Nevertheless, immobilizing POMs in porous solid materials, such as silica and activated carbon is a promising approach to stabilize POMs and optimize their catalytic performance^12^,^13^,^14^,^15^. Among these solid supports, porous metal-organic frameworks (MOFs) offer significant advantages of high surface area and porosity over the traditional solid supports^16^,^17^,^18^,^19^,^20^,^21^,^22^,^23^,^24^,^25^,^26^,^27^,^28^,^29^. Recently, several POMs have been encapsulated into several known MOFs. The resulted POM@MOFs have been applied to alkene epoxidation, oxidative desulfurization, aerobic decontamination, asymmetric dihydroxylation of olefins, and so on^30^,^31^,^32^,^33^,^34^,^35^,^36^,^37^,^38^. Among the reported POM@MOFs, POM@MIL-101 series have been the most investigated because of their large surface areas as well as unique chemical stability^39^,^40^,^41^,^42^,^43^,^44^. In addition to the POM@MIL-101, the POM@HKUST-1 series have been as well intensively studied that display unique catalytic selectivity and conversion in the oxidation of the mercaptans to disulfides and hydrolysis of esters^45^,^46^. Nevertheless, the current studies of POM@MOFs are mostly focused on MIL-101 and HKUST-1^47^,^48^,^49^,^50^. It remains great challenge to the immobilization of POMs into MOFs towards diverse structures and multifunctionalities. It is known that the rht**-**MOF**-**1 is highly porous with large surface area and possess a high concentration of open metal sites (OMSs). It contains four types of cage: cuboid (~5.9 Å), rhombitruncated cuboctahedral (~11.6 Å), β -cage like (~12.1 Å), and α -cage like (~20.2 Å) accessible through microporous quadrate windows (~6 Å), which is a potential host framework to encapsulate POMs that may be applied as catalysts^51^,^52^. Therefore, attemption of immobilizing the POMs into rht**-**MOF**-**1 was conducted by reactions of rht**-**MOF**-**1 with H_3_PMo_12_O_40_, H_4_SiMo_12_O_40_, and H_3_PW_12_O_40_ in DMF and water, respectively. As a result, a series of three POM@MOFs, [PMo_12_O_40_]@[Cu_6_O(TZI)_3_(H_2_O)_9_]_4_·OH·31H_2_O (HLJU-1), [SiMo_12_O_40_]@[Cu_6_O(TZI)_3_(H_2_O)_9_]_4_·32H_2_O (HLJU-2), and [PW_12_O_40_]@[Cu_6_O(TZI)_3_(H_2_O)_6_]_4_·OH·31H_2_O (HLJU-3) have been isolated. X-ray structure analyses indicate that the Keggin-type POMs are incorporated into the cages of rht**-**MOF**-**1. Catalytic experiments reveal that HLJU 1− 3 exhibit unique catalytic selectivity and reactivity in the oxidation of alkylbenzene under mild condition with environmental benign oxidant in aqueous phase as well as the uptake capacity towards organic pollutants in aqueous solution.

## Results and Discussion

X-ray diffraction analysis reveals that HLJU 1− 3 are isomorphous crystallizing in a highly symmetric space group of *Fm*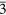 *m* with large cell volume in the range of 87968− 88800 Å^3^. The Keggin-type POMs (H_3_PMo_12_O_40_, H_4_SiMo_12_O_40_, and H_3_PW_12_O_40_) have been first introduced into an open porous system as guests, respectively. The paddle-wheel unit Cu_2_-clusters and triangular inorganic Cu_3_-clusters are connected through the TZI ligands forming a three-dimensional cubic network. Notably, the host framework of HLJU 1− 3 is isostructural with the famous complex rht-MOF-1^52^, indicating that *in situ* preparation of rht-MOF-1 is possible in a mixed solvent of DMF and distilled water in contrast in pure DMF. In a typical structure of HLJU-1, the asymmetric unit of HLJU-1 is of 3 Cu(II) cations, 1/2 triply deprotonated TZI ligand, and 1/12 [PMo_12_O_40_]^3−^ polyoxoanion (abbreviated as PMo_12_) (Figure S1). The PMo_12_ polyoxoanion exhibits the well-known α -Keggin configuration, consisting of a central PO_4_ tetrahedron and four corner-sharing triad {Mo_3_O_13_} clusters. There are three crystallographically independent Cu(II) cations in the structure. Both Cu1 and Cu2 cations adopt the tetragonal pyramid geometry, coordinated by five oxygen atoms, four oxygen atoms from four TZI ligands and one oxygen atoms from axial water molecule. The Cu3 cation is five-coordinated in a trigonal bipyramidal coordination geometry, achieved by three oxygen atoms from three coordinated water molecules and two nitrogen atoms from two coordinated TZI ligands (Figure S2). The TZI ligand is six-coordinated in the hexagonal coordination geometry, achieved by six Cu(II) cations (Figure S3). As a result, the Cu1 and Cu2 cations form a paddle-wheel unit Cu_2_-cluster (Cu_2_(O_2_CR)_4_), and three Cu3 cations form a trinuclear cluster (Cu_3_O(N_4_CR)_3_) (Figure S4).

**Figure 1:**
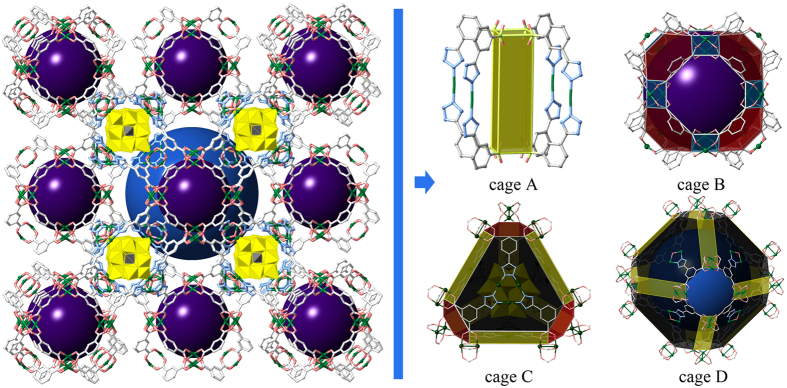
Ball/stick/polyhedral representations of four types of cages, (A–D) in HLJU-1. Color code: Cu, green; N, blue; O, pink; POM, yellow. All H atoms and solvent molecules are omitted for clarity.

**Figure 2:**
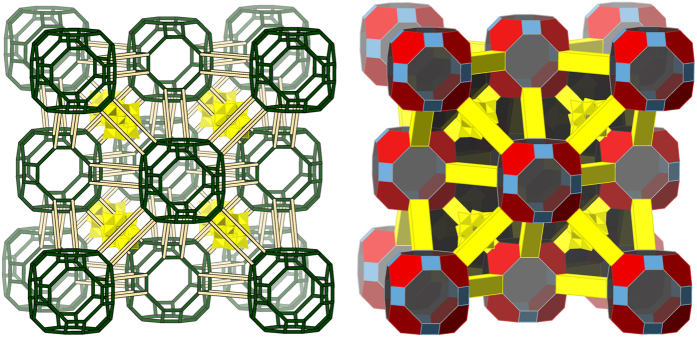
3D HLJU-1 frameworks with *lta* topology. The MOF and POM polyoxoanions are represented by wireframe and polyhedral models.

There are four types of cages (A, B, C and D) with diameters of ca. 5.9, 11.6, 12.1 and 20.2 Å, accessible through the windows for ca. 5.9, 10.1, 7.1 and 8.2 Å, respectively (Fig. 1). Notably, only one of the four cages is occupied by a POM polyoxoanion, while the other filled by solvent molecules. Particularly, Cage A displays a cuboid shape which is constructed by two paddle-wheel unit Cu_2_-clusters and four Cu(N_4_CR)_2_ edges (Figure S5). Cage B provides a rhombitruncated cuboctahedron in which the unit Cu_2_(O_2_CR)_4_ constructs the twelve square, eight hexagonal and six octagonal planes, and carbon atoms of Cu_2_(O_2_CR)_4_ locate on the 48 vertices (Figure S6). Obviously, the relative small diameters of cage A and cage B are not able to encapsulated the Keggin polyoxoanions (~10.5 Å). However, cage C, filling with the Keggin polyoxoanions, is of a β -cage like shape constructed by four large [Cu_3_O(N_4_CR)_3_]_3_ hexagon, four small [Cu_2_(O_2_CR)]_3_ hexagon, and six Cu_2_[Cu_2_(TZI)_2_]_2_ rectangle, in which the carbon atoms of Cu_2_(O_2_CR)_4_ locate on the 24 vertices (Figure S7). While, cage D with α -cage like shape is assembled by eight large [Cu_3_O(N_4_CR)_3_]_3_ hexagon, six [Cu_2_(O_2_CR)]_4_ octagon and twelve Cu_2_[Cu_2_(TZI)_2_]_2_ rectangle (Figure S8). On the basis of the very large cavity, the cage D is as well not suitable to encapsulate the Keggin polyoxoanions due to the week interaction between the framework and POMs. The overall structure of HLJU-1 can be abbreviated as the ***lta*** topology (Fig. 2). The total solvent-accessible volume for HLJU 1− 3 was estimated to be ~68% (~75% for rht-MOF-1) by summing voxels more than 1.2 Å away from the framework using PLATON software^53^,^54^,^55^. Strikingly, the pores are connected in nonlinear channels and facilitate reactant access and product departure. Each encapsulated POM can be accessed via eight adjacent pores. To the best of our knowledge, the verified structure of HLJU 1− 3 is the second type of porous POM@MOF defined by crystal structure after POM@HKUST-1 series. The electron paramagnetic resonance (EPR) of HLJU 1− 3 exhibited the characteristic signal of Cu(II) with g =  2.12 (Figure S11).

**Figure 3:**
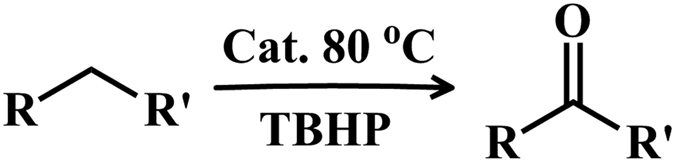
The reaction of alkylbenzenes with TBHP yielding corresponding Phenyl Ketones.

Since HLJU 1− 3 contain catalytically active components of POMs and Cu^II^-MOF, it is encouraged to evaluate the catalytic ability of HLJU 1− 3 in the oxidation reaction of alkylbenzene (Fig. 3). First of all, the contrast test of the catalytic activity among HLJU− 1, rht-MOF-1 and (NBu_4_)_3_PMo_12_O_40_ have been performed in the methylbenzene oxidation, respectively (Table 1, entries 1− 3). GC− MS analysis showed that HLJU-1 exhibits the best conversion of 99% and single selectivity of benzoic acid (Table 1, entry 1), proving that the insertion of the POM into MOFs significantly enhanced the selectivity and reactivity for the oxidation reaction of alkylbenzene. Further contrast experiments of catalytic activity on oxidation of ethylbenzene reveal that HLJU-1 readily afforded acetophenone in the highest yield of 81% among HLJU 1− 3 (Table 1, entry 4-6). The catalytic activity clearly indicates the difference of the POM polyoxoanions among HLJU 1− 3, which is consistent with known sequence of [PMo_12_O_40_]^3−^ >  [PW_12_O_40_]^3−^  >  [SiMo_12_O_40_]^4− ^56. To investigate the effect of the size of the substrate on the catalytic conversions and selectivity, several substrates of alkylbenzene have been employed in the catalytic reactions by HLJU-1. As a result, (Table 1, entries 7− 10) the catalytic conversions decrease along with the size increase of the alkylbenzenes, which is attributed to that the reactions may occur only on the solid surfaces. To further identify the catalytic sites of the alkylbenzene (e.g., on the crystal surfaces or in the open channels), the reaction time was extended to 36 h. The conversions of oxidation reaction reveal that the smallest ethylbenzene and the largest 4-ethylbiphenyl were not obvious increased along with the reaction time increase (8% and 12%, respectively).

**Figure 4:**
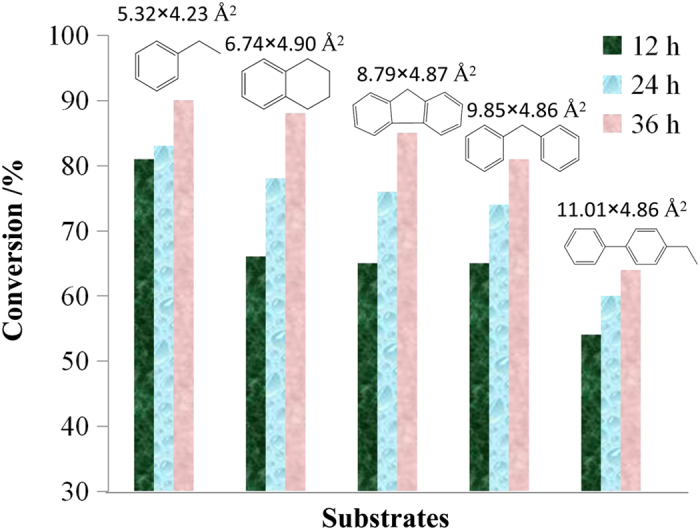
Conversions of oxidation reactions of alkylbenzenes with different sizes for the formation of phenylketones. Catalyst (0.005 mmol), alkylbenzene (1 mmol) and TBHP (5 mmol) were stirred at 80 °C for 12 h, 24 h and 36 h. Conversions are based on GC analysis. The molecular size of substrate is indicated at the top of each column.

However, the medium size of tetrahydronaphthalene, fluorene, and biphenyl methane exhibits a remarkable increase (23%, 21% and 16%, respectively) (Fig. 4). Thus, the corelationship between reaction rate and steric effect suggests that ethylbenzene can be diffused through the pores and touched the inner POM polyoxoanions. In contrast, tetrahydronaphthalene, fluorene, and biphenyl methane with larger steric effect are not diffused through the pores. They may absorb onto the surface pore containing Keggin complexes, leading to lower reaction rate. It should be noticed that the different catalytic conversions among the similar steric effect of fluorine, biphenyl methane, and 4-ethylbiphenyl may result from the activation of their benzene rings. The phenyl group would activate the adjacent C_sp3_-H of benzyl group for the oxidation reactions. Thus, the fluorene and biphenyl methane with two phenyl groups possess the high conversion and reaction rate. Strikingly, HLJU-1 is recyclable up to at least the 5th cycle without losing its reactivity and selectivity under the reaction conditions. The recycled catalyst can be reused for these reactions after simple filtration, washing with acetonitrile, and drying. The PXRD patterns of HLJU-1 remain almost unchanged before and after the catalytic reactions, indicating the high stability and immobility of HLJU-1 (Figure S17).

We monitored the accessibility of the open channels to several substrate molecules and TBHP oxidant by ^1^H NMR and GC-MS (see the SI for details). The NMR spectrum is clearly indicative of pore accessibility to ethylbenzene molecules and TBHP. A more quantitative analysis was conducted by GC− MS, from which uptake amounts of 16.9 wt % (for ethylbenzene) and 18.2 wt % (for TBHP) were obtained. On the contrary, no detectable amount was observed by ^1^H NMR analysis for larger substrate molecules such as tetrahydronaphthalene, fluorene, and biphenyl methane under the same experimental conditions. These combined results suggest that the Cu(II) sites in the channel walls and POM polyanions in voids are indeed reachable by substrates of relatively small sizes, thus allowing much higher catalytic performance than in the case of larger substrates. The latter molecules have difficulty entering the interior pore spaces, and reactions can only occur at exterior solid surfaces.

Immobilization of POM polyanions results in high selectivity and reactivity than single POM polyanions and MOF. It is likely that electrostatic interactions between the solvent accessible Cu(II) centers of the MOF structure and the encapsulated [PMo_12_O_40_]^3−^ units are present in HLJU− 1, and these stabilize HLJU− 1 relative to its components. Such electrostatic POM− MOF interactions could simultaneously increase the rates of the substrate oxidation [PMo_12_O_40_]^3−^ reduction step in the overall oxidations catalyzed by the POM@MOF. It is speculated that the mechanism of the catalytic reaction inside the pores of HLJU− 1 involves multiple steps and the proposed mechanism, which are list in supporting information (see Scheme S1).

**Figure 5:**
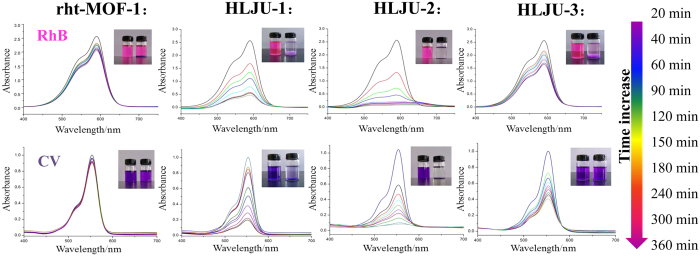
(top) UV−vis spectra of rhodamine B after the addition of solid rht-MOF-1 and HLJU 1−3 as time increase. (bottom) UV− vis spectra of crystal violet after the addition of solid rht-MOF-1 and HLJU 1− 3 as time increase. The inset photographs highlight the scavenging effects.

The toxicities of dyes have brought about a significant threat to the aqueous environment and caused serious consequences, such as aesthetic pollution, even carcinogenicity and perturbation to aquatic life. Nevertheless, most dyestuffs are difficult to degrade because of their stability to light and oxidants^57^. MOFs and POMs have been extensively indagated for adsorption and degradation dye molecules^58^,^59^,^60^,^61^,^62^. However, MOFs and POMs exhibit several weak points: for MOFs, the relative low stability in solution and brittleness or lack of flexibility; for POMs, feasible dissolution in water or polar organic liquids and relatively low surface area, which hampering their realistic applications. Recently, there have been two reports involved in the use of POM@MOFs composite as dyes adsorbent in Wang’s group and Yang’s group, which indicated that the combination of POM@MOFs could overcome the defects of each component^65,66^. Then HLJU 1− 3 are applied to remove dyes from aqueous solutions. To contrast the adsorption activity of rht-MOF-1 and HLJU 1− 3, the UV/visible absorption spectra of rhodamine B and crystal violet solution in the presence of rht-MOF-1 and HLJU 1− 3 were conducted, respectively. As shown in Fig. 5, the uptake capacity of the HLJU 1− 3 are obviously higher than that for rht-MOF**-**1. It is attributed to effect of the charges from the POM polyoxoanions on the uptake capacity. While the uptake capacity of the HLJU-2 is obviously higher than that for HLJU-1 and HLJU-3, attributing to that the electron charge of polyoxoanions SiMo_12_^4−^ in HLJU-2 is more than those of PMo_12_^3−^ and PW_12_^3−^ in HLJU-1 and HLJU-3 respectively. This result indicates that the more negative charges the more uptake capacity. It is worth to note that the crystal violet uptake capacity of HLJU-2 (0.093 mmol·g^-1^) is much higher than that of {[Cd(DMF)_2_-Mn^III^(DMF)_2_TPyP](PW_12_O_40_)}·2DMF·5H_2_O (0.057 mmol·g^−1^), a layered POM-Mn^III^-metalloporphyrin-based hybrid material^36^. The rhodamine B uptake capacity of HLJU-2 (10 mmol·g^−1^) is higher than that of H_6_P_2_W_18_O_62_@MOF-5 (9 mmol·g^−1^) and lowed than that of PW_11_V@MIL-101 (40 mmol·g^−1^)^63^,^64^. The adsorption of the rhodamine B occurs in the open channels, which lead to the higher uptake capacity of PW_11_V@MIL-101. Since the size of windows in HLJU 1− 3 (5.9 Å) is much smaller than the diameters of the dyes (10.8 Å for rhodamine B and 13.2 Å for crystal violet), it can be concluded that the adsorption of the dyes occurs on the solid surfaces.

## Conclusions

Isolation of POM@MOF HLJU 1− 3 first demonstrates that the Keggin POMs can be immobilized into the *β*-cage of rht-MOF-1 by a solvothermal method with highly ordered and porous structure. The highly ordered structure results in the well dispersion of POMs that synergistically promote the catalytic oxidation activity of alkylbenzenes, while highly porous structure with plentiful POM polyoxoanions enhance the adsorption efficiency for RhB and crystal violet dyes. Significantly, various pore dimensions in HLJU 1− 3 afford an opportunity for selection of substrates in the catalytic reactions. This approach may inspire the study on immobilization of POMs into various MOFs to construct functional porous frameworks as heterogeneous catalysts and pollutants scavenger.

## Methods

### Synthesis of HLJU 1**−**3

A solution of CuCl_2_·2H_2_O (0.03 g, 0.18 mmol), 5-tetrazolylisophthalic acid (H_3_TZI) (0.011 g, 0.047 mmol) and H_3_PMo_12_O_40_·*n*H_2_O (0.1 g, 0.055 mmol) for HLJU-1, H_3_SiMo_12_O_40_·*n*H_2_O (0.1 g, 0.055 mmol) for HLJU-2, and H_4_PW_12_O_40_·*n*H_2_O (0.1 g, 0.035 mmol) for HLJU-3 in 1 mL of *N,N*-dimethylformamide (DMF) and 1 mL of distilled water was heated to 85 °C for 12 h, followed by slow cooling to room temperature. Blue or green octahedral crystals of HLJU 1− 3 were then collected. The entire yields for HLJU 1− 3: 60− 80% based on Cu. IR (KBr, cm^−1^) for HLJU-1: 1626(s), 1566(s), 1438(s), 1387(s), 1273(w), 1048(w), 942(m), 870(m), 808(m), 752(w); for HLJU-2: 1646(s), 1570(s), 1435(s), 1387(s), 1251(w), 1108(m), 957(s), 905(m), 784(s), 751(s), 731(s); for HLJU-3: 1651(s), 1566(s), 1501(s), 1436(s), 1389(s), 1250(w), 1101(m), 1059(m), 956(m), 884(w), 811(w), 751(s), 733(s). Elemental Anal. Calcd (Found %) for C_108_H_171_N_48_Cu_24_PMo_12_O_160_ (7409.10)(HLJU-1): C, 17.51(17.53); H, 2.33(2.36); N, 9.07(9.09); for C_108_H_172_N_48_Cu_24_SiMo_12_O_160_ (7407.22)(HLJU-2): C, 17.51(17.54); H, 2.34(2.38); N, 9.08(9.11); for C_108_H_147_N_48_Cu_24_PW_12_O_148_ (8247.72)(HLJU-3): C, 15.73(17.75); H, 1.80(1.83); N, 8.15(8.18).

### Crystal data for HLJU-1

(CCDC no. 1058671): C_108_H_171_N_48_Cu_24_PMo_12_O_160_, *M* =  7409.10, cubic, a =  b =  c =  44.599(3) Å, α  =  β  =  γ  =  90.00°, *V* =  88712(18) Å^3^, *T* =  293(2) K, space group F*m*
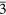
*m*, Z =  8, 18345 reflections measured. The final *R*_1_ values were 0.0875 (*I* >  2*σ*(*I*)). The final *wR* (*F*^2^) values were 0.2584 (*I* >  2*σ*(*I*)). The final *R*_1_ values were 0.0997 (all data). The final *wR* (*F*^2^) values were 0.2696 (all data).

### Crystal data for HLJU-2

(CCDC no. 1058599): C_108_H_172_N_48_Cu_24_SiMo_12_O_160_, *M* =  7407.22, cubic, a =  b =  c =  44.614(5) Å, α  =  β  =  γ  =  90.00°, *V* =  88800(30) Å^3^, *T* =  293(2) K, space group F*m*
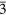
*m*, Z =  8, 16911 reflections measured. The final *R*_1_ values were 0.0710 (*I* >  2*σ*(*I*)). The final *wR* (*F*^2^) values were 0.2017 (*I* >  2*σ*(*I*)). The final *R*_1_ values were 0.0967 (all data). The final *wR* (*F*^2^) values were 0.2188 (all data).

### Crystal data for HLJU-3

(CCDC no. 1058600): C_108_H_147_N_48_Cu_24_PW_12_O_148_, *M* =  8247.72, cubic, a =  b =  c =  44.742(7) Å, α  =  β  =  γ  =  90.00°, *V* =  87968(4) Å^3^, *T* =  293(2) K, space group F*m*
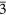
*m*, Z =  8, 3470 reflections measured. The final *R*_1_ values were 0.0994(*I* >  2*σ*(*I*)). The final *wR* (*F*^2^) values were 0.2314 (*I* >  2*σ*(*I*)). The final *R*_1_ values were 0.1302 (all data). The final *wR* (*F*^2^) values were 0.2547 (all data).

### Characterization

All of the chemicals were obtained from commercial sources and used without further purification. Elemental (C, H and N) analyses were performed on a Perkin-Elmer 2400 analyzer. FT-IR data were collected on a Perkin-Elmer 100 spectrophotometer by using KBr pellets in the range of 4500− 450 cm^−1^. Thermal analyses were carried out on a STA-6000 with a heating rate of 10 °C min^−1^ in a temperature range from 30 °C to 800 °C in atmosphere. Powder X-ray diffraction (PXRD) data were recorded on a Rigaku D/Max-3B X-ray diffractometer with CuKα as the radiation source (λ  =  0.15406 nm) in the angular range θ  =  5− 50° at room temperature. GC− MS spectra were recorded on a SHIMADZU GCMS-QP2010. Nuclear magnetic resonance (NMR) was carried out on a Bruker AVANCE III 500 system. The concentration of dyes was analyzed by a UV-visible spectrophotometer (Perkin-Elmer 35), which recorded the temporal UV-visible spectral variations of the dyes with characteristics absorbance peak. Electron paramagnetic resonance (EPR) spectra were recorded on a EMX-10/12 spectrometer.

### Typical procedure for oxidation of alkylbenzenes

Oxidation reactions were performed for six alkylbenzenes: methylbenzene, ethylbenzene, fluorine, tetrahydronaphthalene, biphenyl methane, and 4-ethyl-1,1′ -biphenyl. In a typical reaction, ethylbenzene (1 mmol), TBHP (5 mmol), and catalyst (0.005 mmol) were allowed to stir at 80 °C for 12 h. The conversion and selectivity were obtained by GC analysis using a capillary SE-54 column with a flame ionization detector (FID). After the reaction, the catalyst was separated by filtration subjected to a recycling experiment after full washing and heated at 100 °C for 6 h under vacuum.

### Dye Adsorption Experiment

For Rhodamine B: adsorbent (50 mg) was added into a 50 mL aqueous solution of rhodamine B (9.5 mg·L^−1^) under stirring at room temperature. After 30 min, the solution was centrifuged, and the plasma was analyzed by UV-vis absorption spectroscopy. The amount of adsorbed dyes was calculated (Eq. 1).


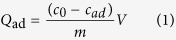


where Q_ad_ (mmol/g) is the amount of adsorbed dyes by adsorbent **1**, C_0_ is the initial concentration of dyes in the water (mmol/L), C_ad_ is the concentration of dyes after adsorption (mmol/L), V is the volume of the solution (L), and m is the mass of adsorbent **1** (g).

For crystal violet: adsorbent (50 mg) was added into a 50 mL aqueous solution of crystal violet (15 mg·L^−1)^ under stirring at room temperature. After 30 min, the solution was centrifuged, and the plasma was analyzed by UV-vis absorption spectroscopy.

## Additional Information

**How to cite this article**: Sun, J.-W. *et al.* Immobilization of Polyoxometalate in the Metal-Organic Framework rht-MOF-1: Towards a Highly Effective Heterogeneous Catalyst and Dye Scavenger. *Sci. Rep.* **6**, 25595; doi: 10.1038/srep25595 (2016).

## Supplementary Material

Supplementary Information

## Figures and Tables

**Table 1:**
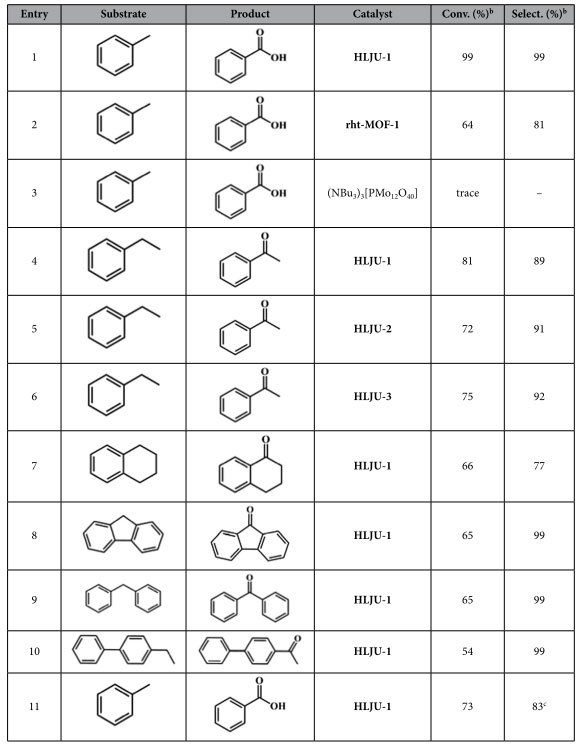
Oxidation of Alkylbenzenes to Phenyl Ketones Catalyzed by POM−MOFs^a^.

^a^Alkylbenzene (1 mmol), TBHP (5 mmol), and catalyst (0.005 mmol) were stirred at 80 °C for 12 h.

^b^Conversion (%) and selectivity (%) were determined by GC-MS on an SE-54 column.

^c^Fifth cycle, and the byproduct is 1-phenylethanol.
