# Supplementary material for: Immobilization of Polyoxometalate in the Metal-Organic Framework rht-MOF-1: Towards a Highly Effective Heterogeneous Catalyst and Dye Scavenger
Source: Sci Rep. 2016 May 9;6:25595. doi: 10.1038/srep25595 (PMC4860640; doi:10.1038/srep25595)
Supplement: Supplementary Information [file srep25595-s1.pdf]

Immobilization of Polyoxometalate in the Metal-Organic Framework rht-MOF-1: Towards a Highly Effective Heterogeneous Catalyst and Dye Scavenger

Jing-Wen Sun,[1] Peng-Fei Yan,[1,*] Guang-Hui An,[1] Jing-Quan Sha,[3] Guang-Ming Li[1,*] and Guo-Yu Yang,[2,*]

[1]*MOE Key Laboratory of Functional Inorganic Material Chemistry, P. R. China; School of Chemistry and Materials Science, Heilongjiang University; Harbin, Heilongjiang 150080, P. R. China*

[2]*MOE Key Laboratory of Cluster Science, School of Chemistry, Beijing Institute of Technology, Beijing 100081, China*

[3]*School of Pharmacy, Jiamusi University, Jiamusi, Heilongjiang 154007, P. R. China*

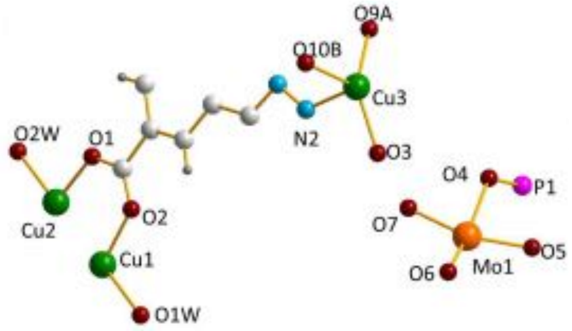

Fig. S1 Asymmetric unit of HLJU-1. Color code: Cu(green), N(blue), C(white), O(red), Mo(orange), P(pink).

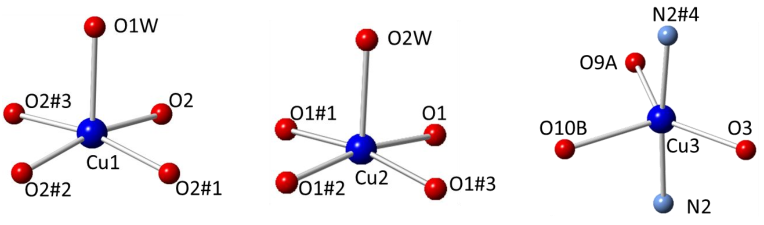

Fig. S2 The coordination environment of the Cu^{II} center in HLJU-1. Color code: Cu(blue), N(light blue), O(red). Symmetry code: #1, y, x, z; #2, y, x, -z; #3, x, y, -z; #4, 0.5-z, y, 0.5-x.

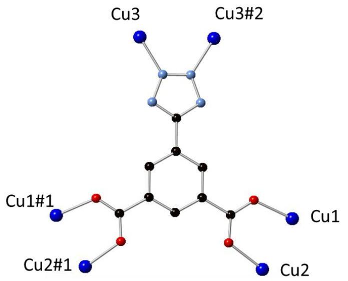

Fig. S3 The coordination environment of the ligand in HLJU-1. Color code: Cu(blue), N(light blue), O(red), C(black). Symmetry code: #1, x, -y, z; #2, 0.5-z, 0.5-x, y.

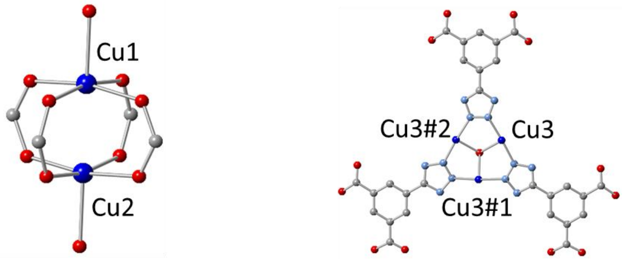

Fig. S4 The di-nuclear Cu cluster and the tri-nuclear Cu cluster in HLJU-1. Color code: Cu(blue), N(light blue), O(red), C(white). Symmetry code: #1, 0.5-y, z, 0.5-x; #20.5-z, 0,5-x, y.

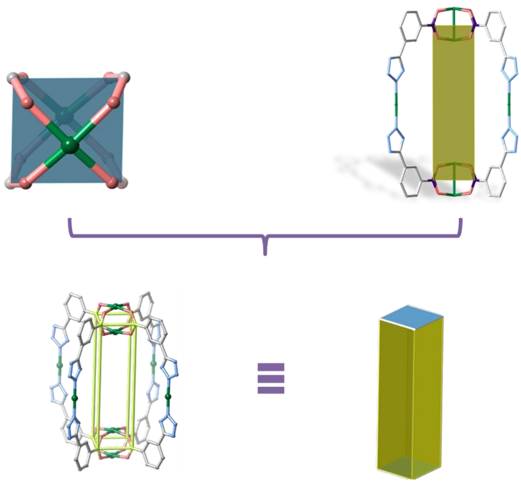

Fig. S5 Ball/stick/polyhedral representations of cage A in HLJU-1. Color code: Cu, green; N, blue; O, pink.

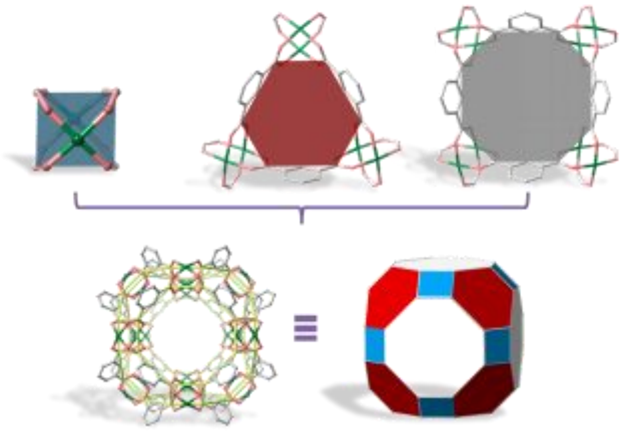

Fig. S6 Ball/stick/polyhedral representations of cage B in HLJU-1. Color code: Cu, green; O, pink.

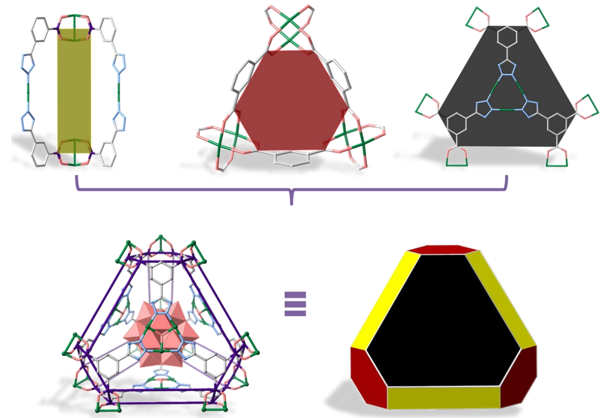

Fig. S7 Ball/stick/polyhedral representations of cage C in HLJU-1. Color code: Cu, green; N, blue; O, pink; POM, pink polyhedra.

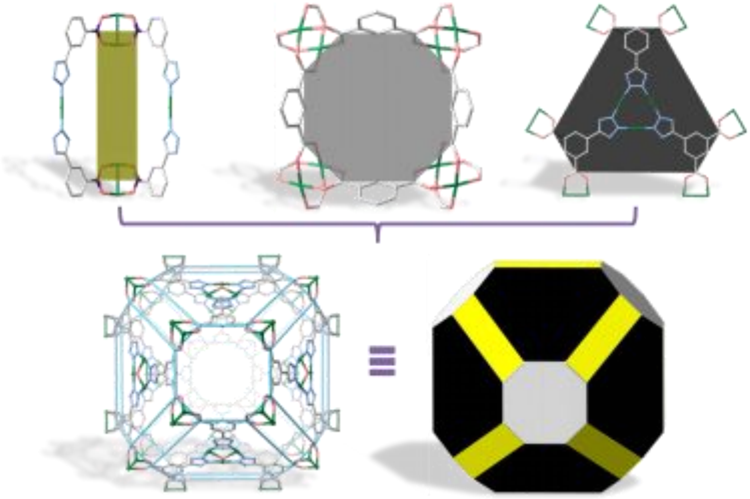

Fig. S8 Ball/stick/polyhedral representations of cage D in HLJU-1. Color code: Cu, green; N, blue; O, pink.

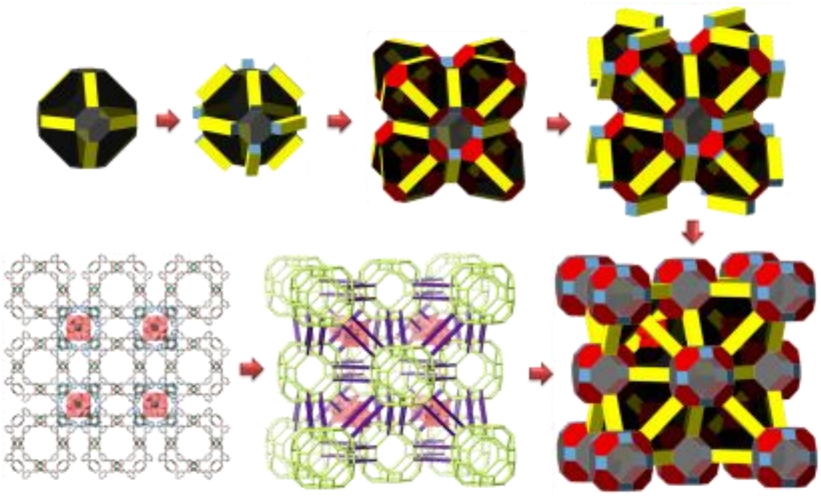

Fig. S9 Schematic showing the corresponding strategy from SBBs to MOFs.

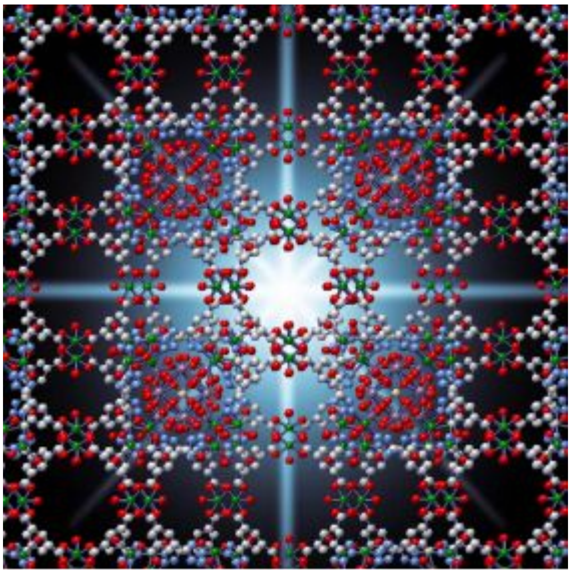

Fig. S10 Ball and stick model of HLJU-1.

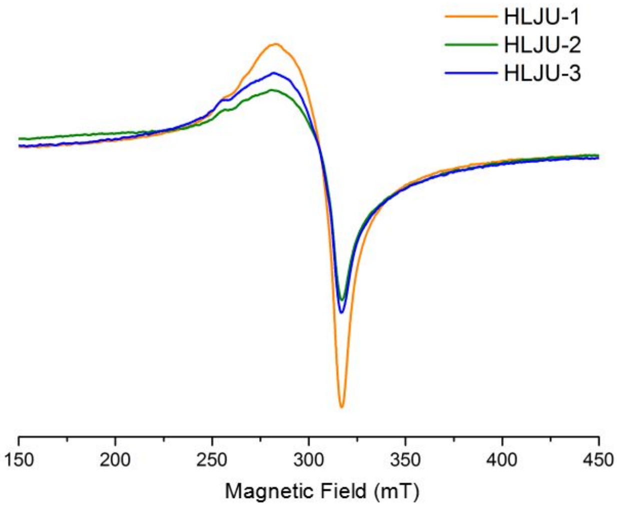

Fig. S11 EPR spectra of HLJU-1 (g=2.1186), HLJU-2 (g=2.1185), and HLJU-3 (g=2.1197) in solid state at 108 K, respectively.

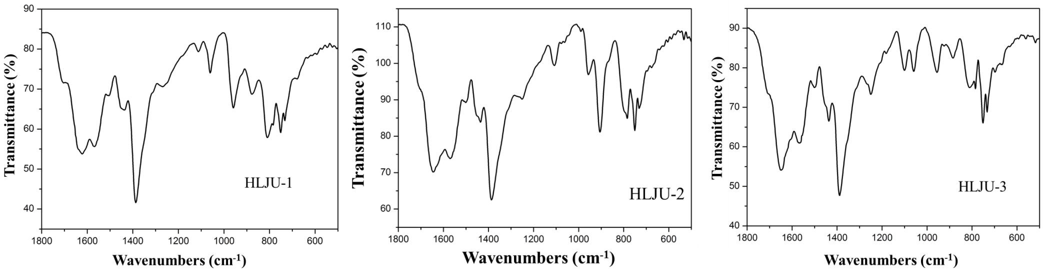

Figure S12. FT-IR spectra of HLJU 1-3.

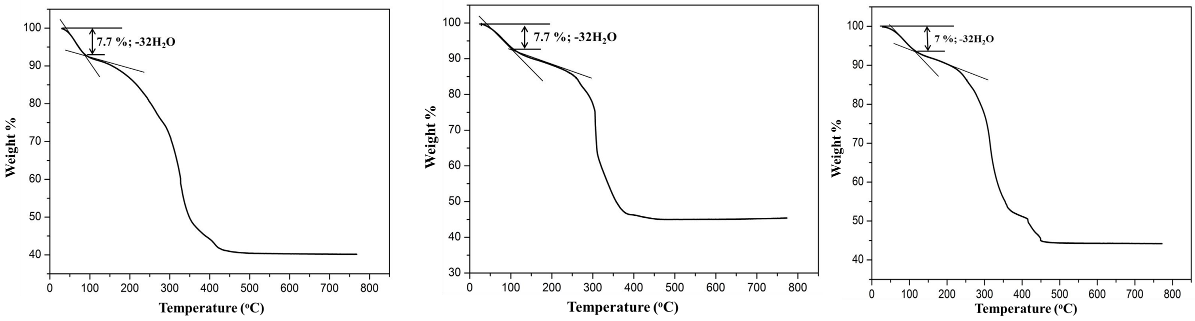

Figure S13. TGA spectra for HLJU 1-3. For HLJU-1, <100 °C, loss of free water molecules; 100-200 °C, loss of coordinated water; >200 °C, framework degradation. For HLJU-2, <100 °C, loss of free water molecules; 100-250 °C, loss of coordinated water; >250 °C, framework degradation. For HLJU-3, <100 °C, loss of free water molecules; 100-250 °C, loss of coordinated water; >250 °C, framework degradation.

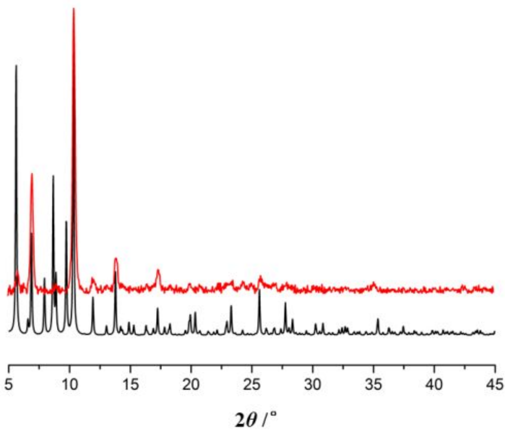

Figure S14. The simulated (black) and experimental (red) PXRD patterns for HLJU-1, respectively.

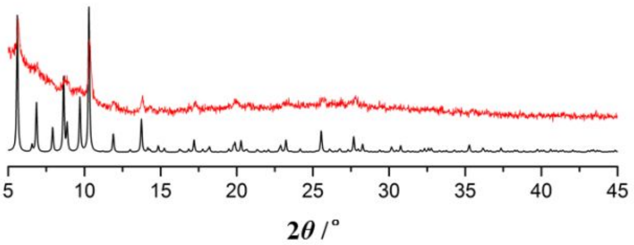

Figure S15. The simulated (black) and experimental (red) PXRD patterns for HLJU-2, respectively.

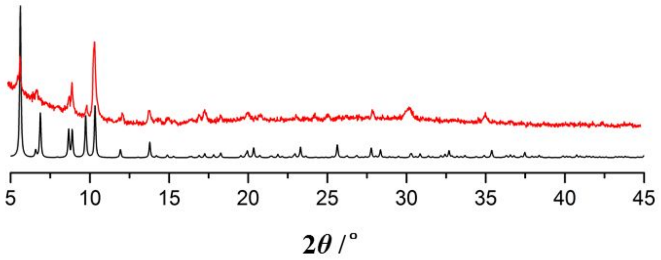

Figure S16. The simulated (black) and experimental (red) PXRD patterns for HLJU-3, respectively.

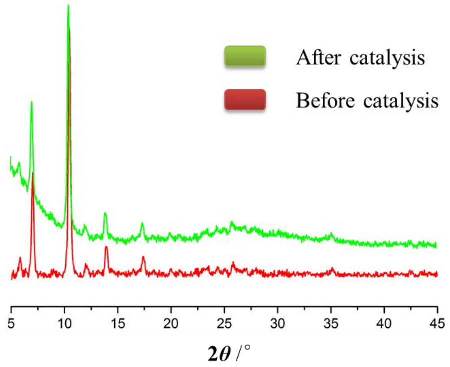

Figure S17. The PXRD patterns of HLJU-1, and HLJU-1 after catalytic. No decomposition is observed.

1H NMR and 13C NMR spectra

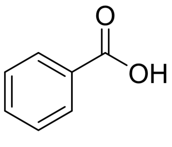

^{1}H NMR (400 MHz, $CDCl_3$) δ 12.23 (br, 1H), 8.28 – 8.00 (m, 2H), 7.85 – 7.55 (m, 1H), 7.55 – 7.44 (m, 2H); ^{13}C NMR (101 MHz, $CDCl_3$) δ 172.44, 133.84, 130.24, 129.35, 128.50.

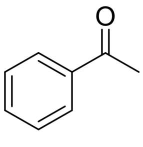

^{1}H NMR (400 MHz, $CDCl_3$) δ 8.09 – 7.84 (m, 2H), 7.68 – 7.51 (m, 1H), 7.51 – 7.40 (m, 2H), 2.60 (s, 3H); ^{13}C NMR (101 MHz, $CDCl_3$) δ 198.11, 137.14, 133.09, 128.56, 128.29, 26.58.

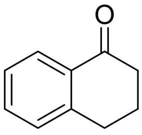

^{1}H NMR (400 MHz, $CDCl_3$) δ 8.03 (dd, J = 7.8, 1.0 Hz, 1H), 7.46 (td, J = 7.5, 1.4 Hz, 1H), 7.36 – 7.15 (m, 2H), 2.96 (t, J = 6.1 Hz, 2H), 2.76 – 2.51 (m, 2H), 2.13 (dt, J = 12.7, 6.4 Hz, 2H); ^{13}C NMR (101 MHz, $CDCl_3$) δ 198.32, 144.49, 133.38, 132.62, 128.78, 127.13, 126.61, 39.16, 29.70, 23.29.

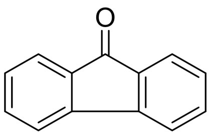

^{1}H NMR (400 MHz, $CDCl_3$) δ 7.64 (d, J = 7.3 Hz, 2H), 7.55 – 7.36 (m, 3H), 7.27 (td, J = 7.1, 1.9 Hz, 2H); ^{13}C NMR (101 MHz, $CDCl_3$) δ 193.84, 144.40, 134.66, 134.12, 129.04, 124.24, 120.30.

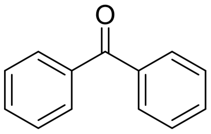

^{1}H NMR (400 MHz, $CDCl_3$) δ 7.94 – 7.67 (m, 4H), 7.60 (t, J = 7.4 Hz, 2H), 7.49 (t, J = 7.7 Hz, 4H); ^{13}C NMR (101 MHz, $CDCl_3$) δ 196.69, 137.63, 132.44, 130.06, 128.31.

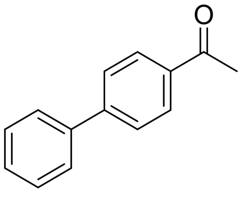

^{1}H NMR (400 MHz, $CDCl_3$) δ 8.13 – 7.95 (m, 2H), 7.78 – 7.68 (m, 2H), 7.65 (d, J = 5.4 Hz, 2H), 7.50 (dd, J = 10.1, 4.7 Hz, 2H), 7.46 – 7.37 (m, 1H), 2.66 (s, 3H); ^{13}C NMR (101 MHz, $CDCl_3$) δ 197.72, 145.78, 139.89, 135.89, 128.98, 128.93, 128.25, 127.29, 127.23, 26.66.

^{1}H NMR and ^{13}C NMR spectra

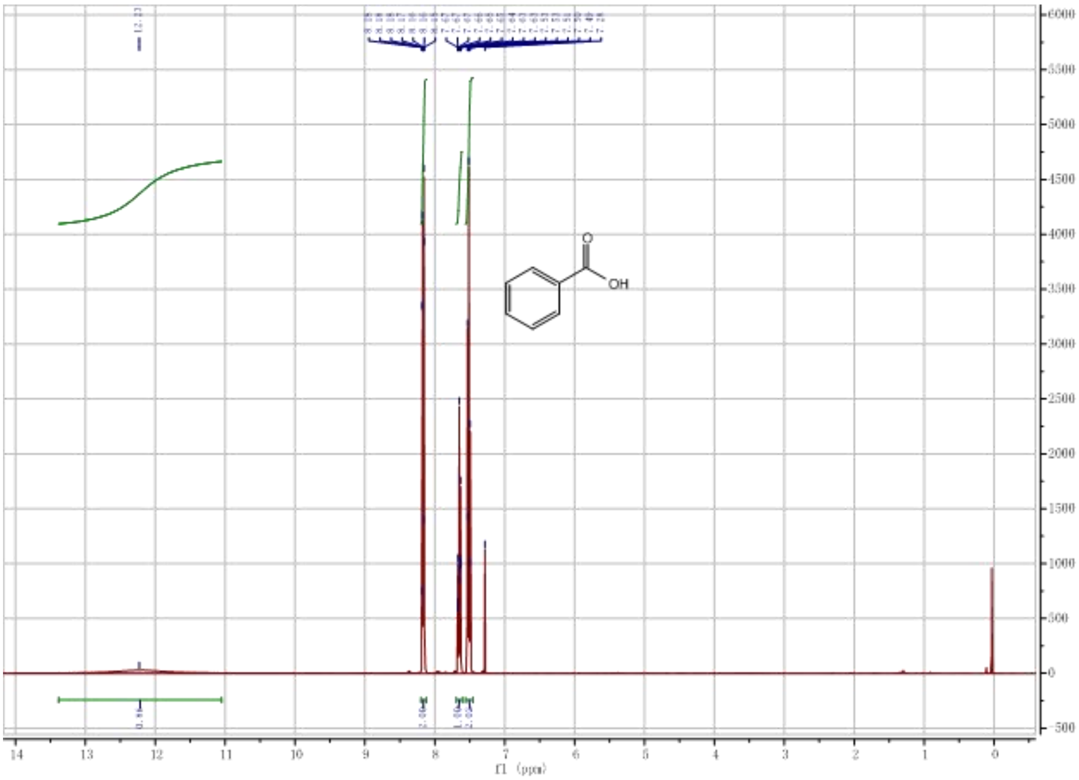

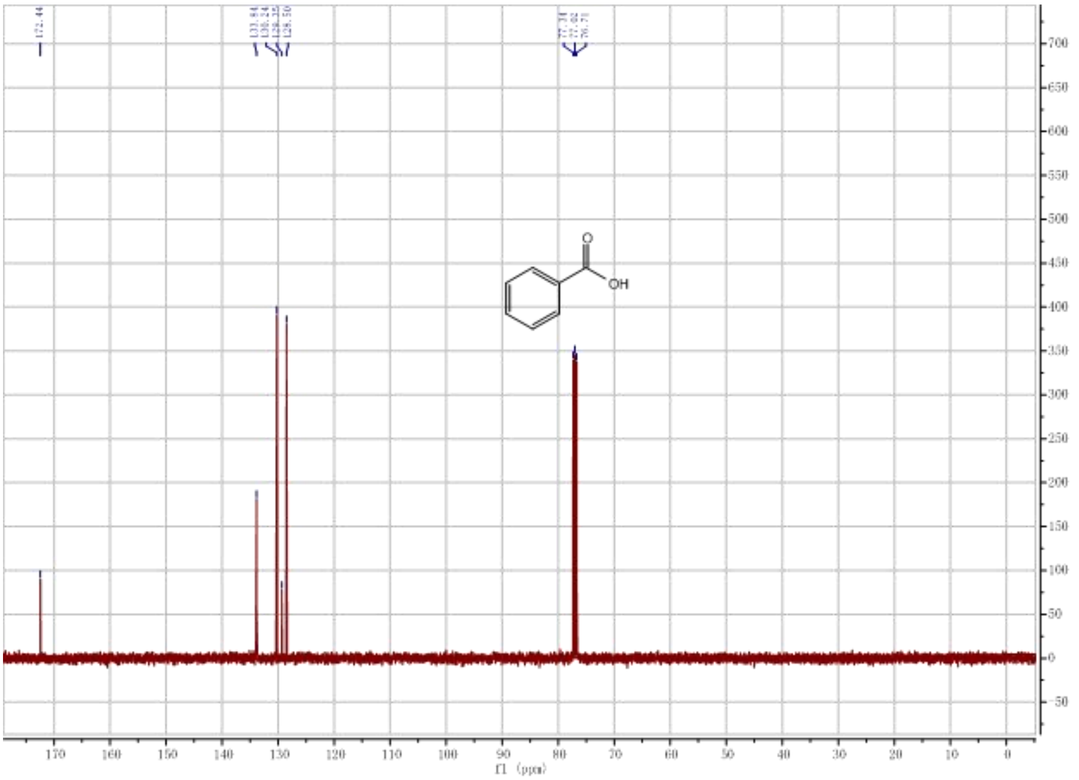

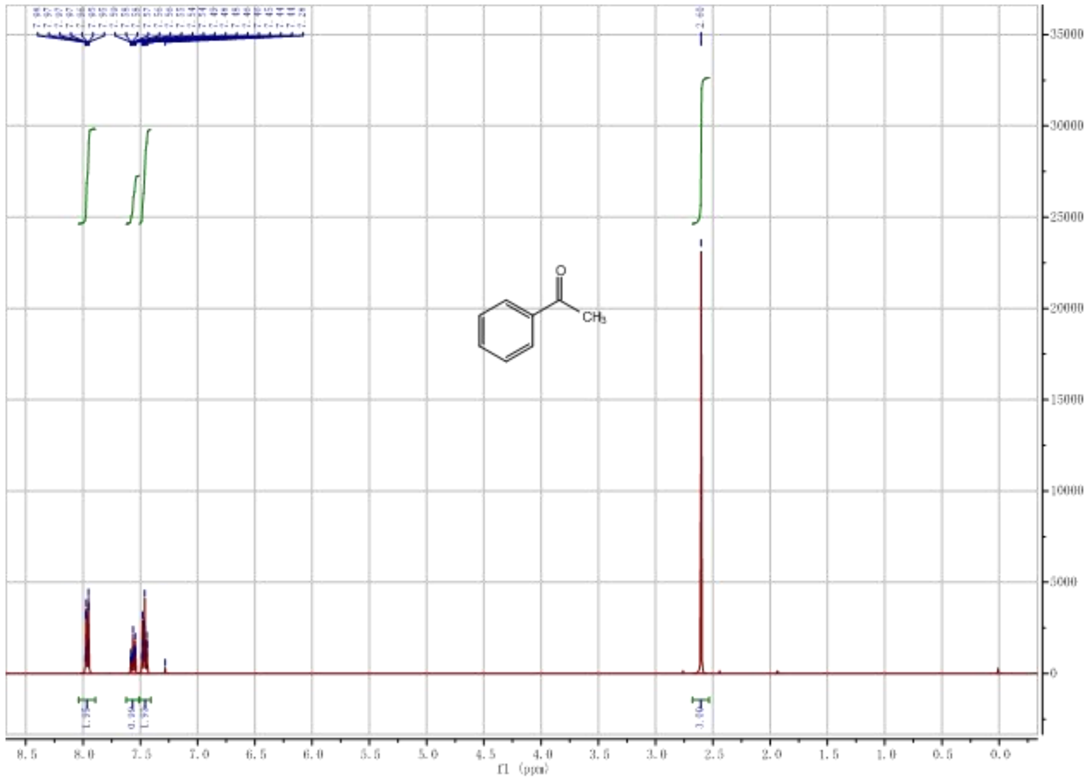

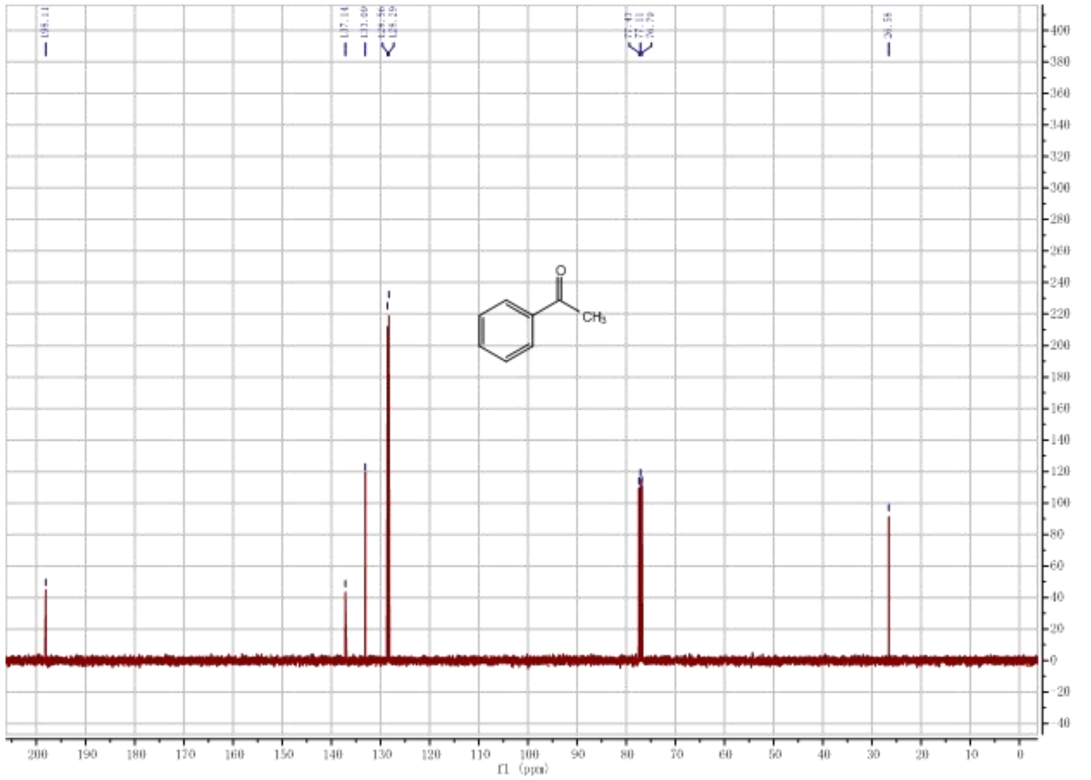

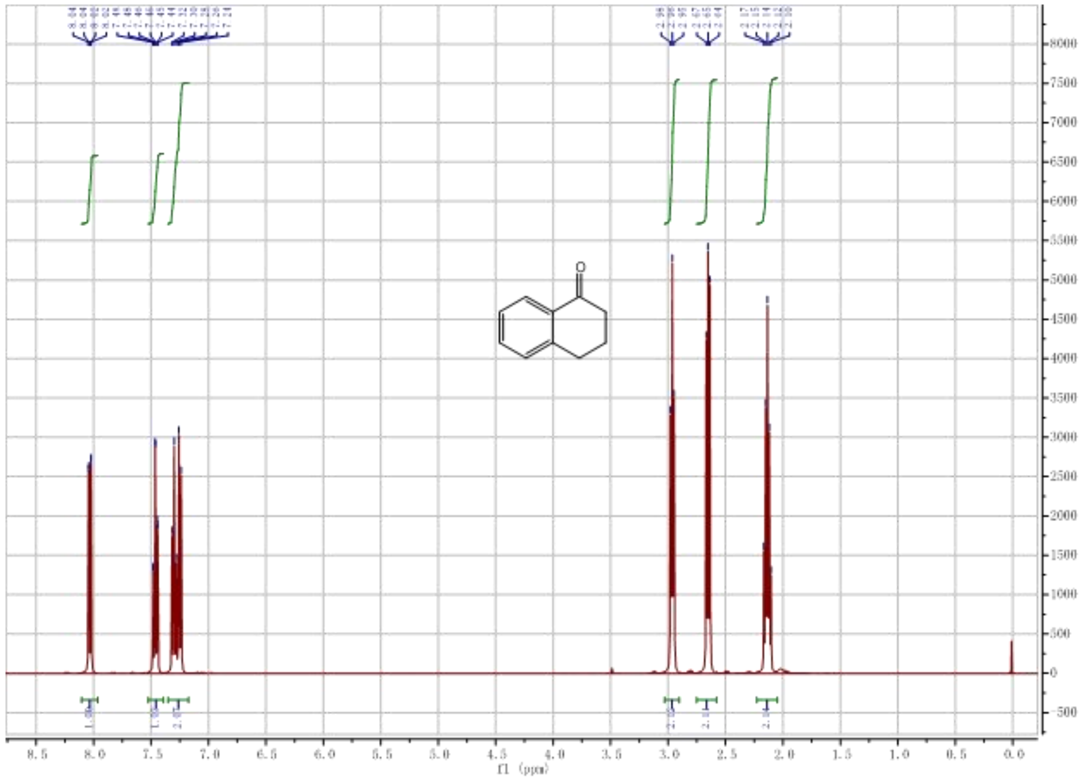

f1 (ppm)

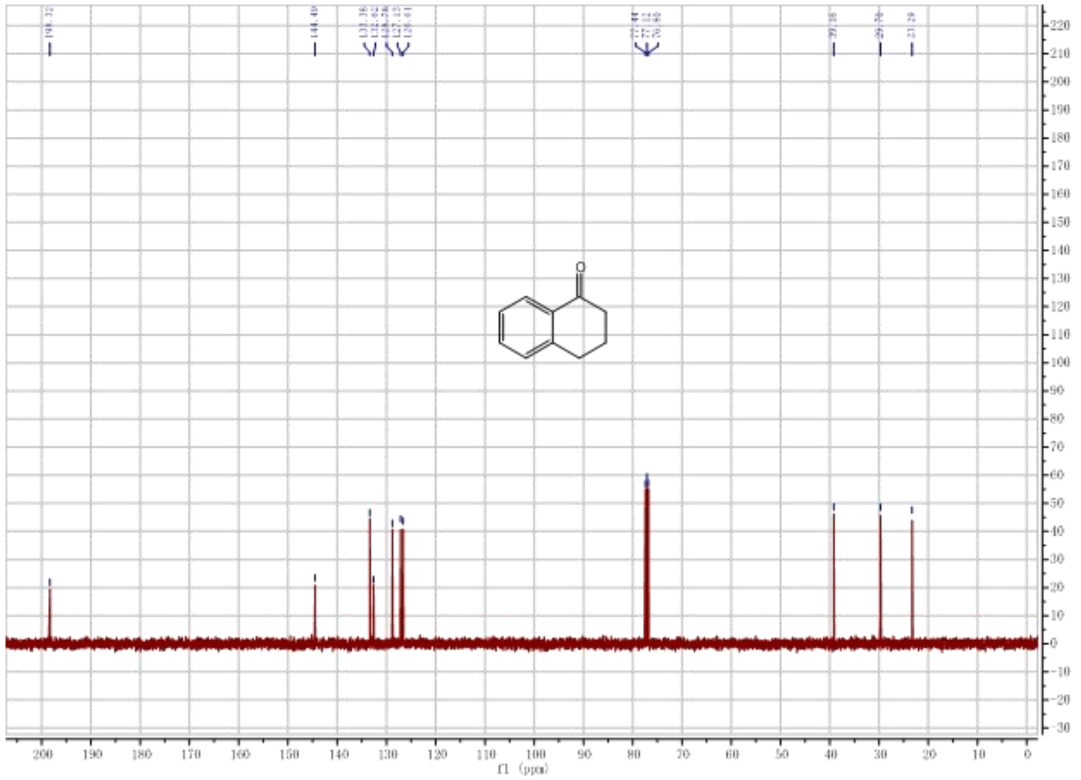

f1 (ppm)

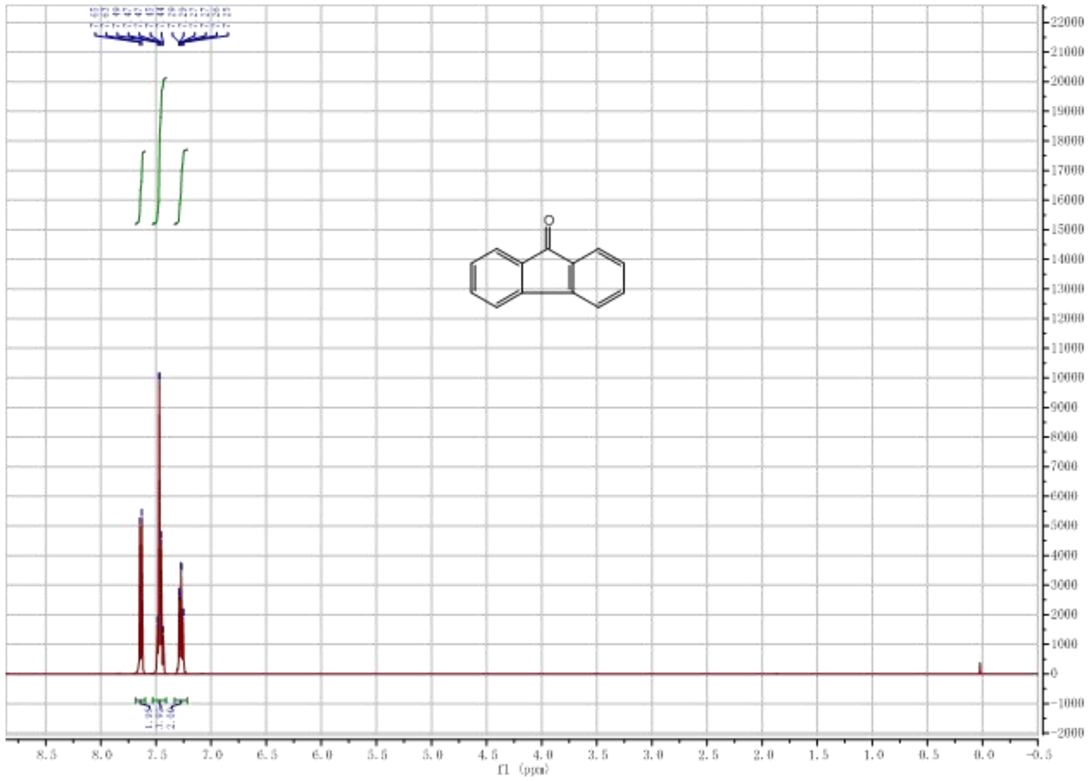
f1 (ppm)

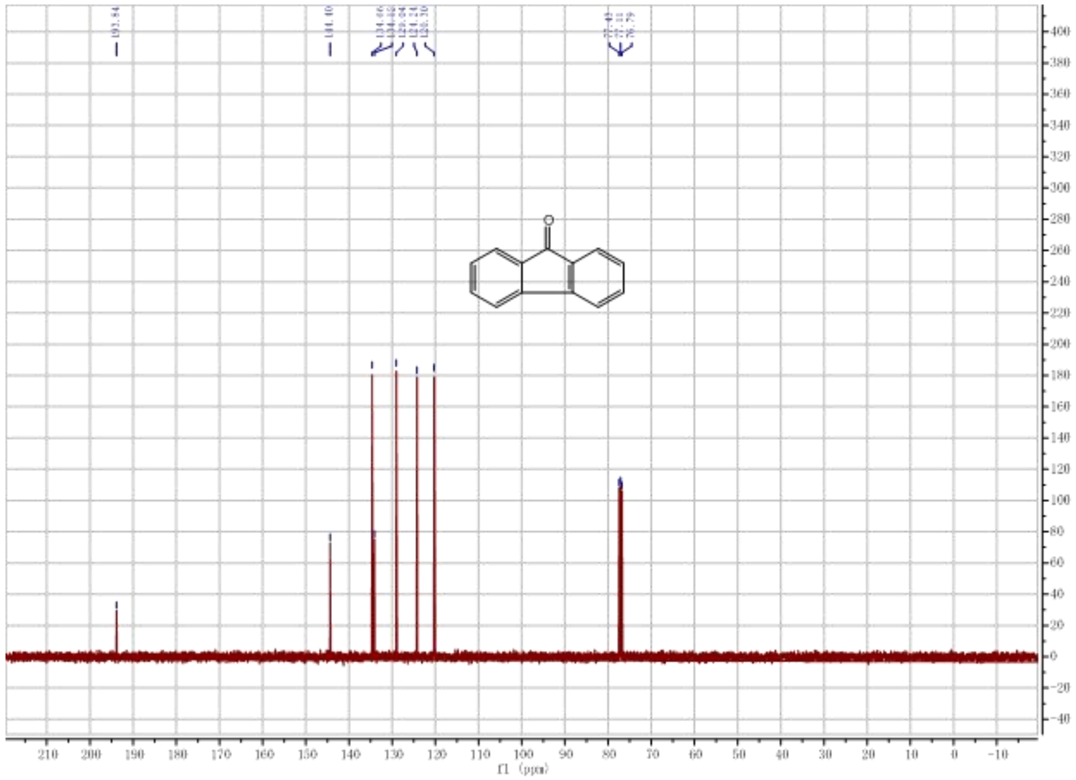
193.84
144.40
f1 (ppm)

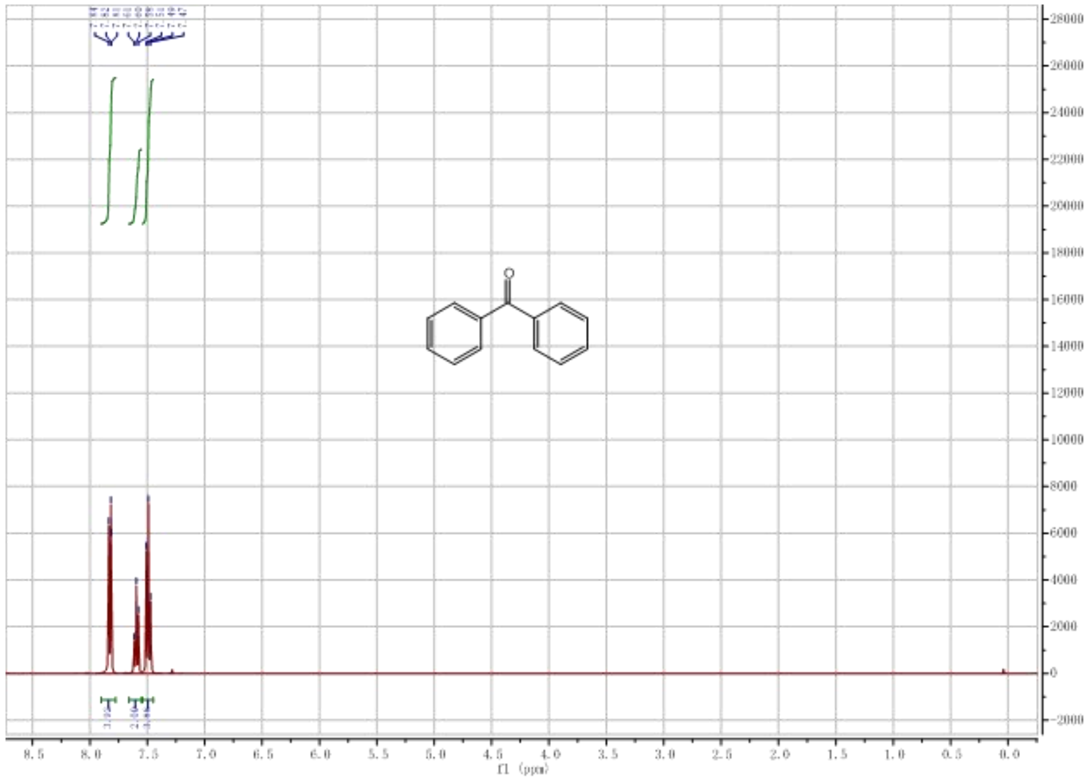

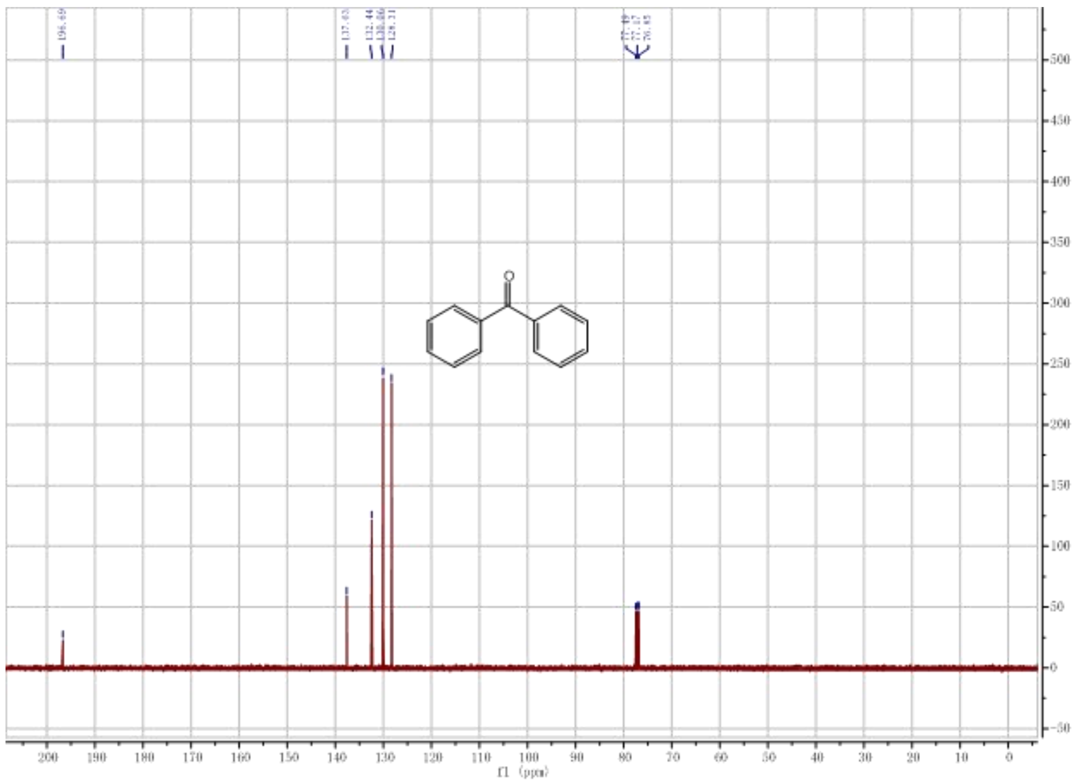

196.69
137.63
132.44
130.06
128.31
77.49
77.17
76.85
f1 (ppm)

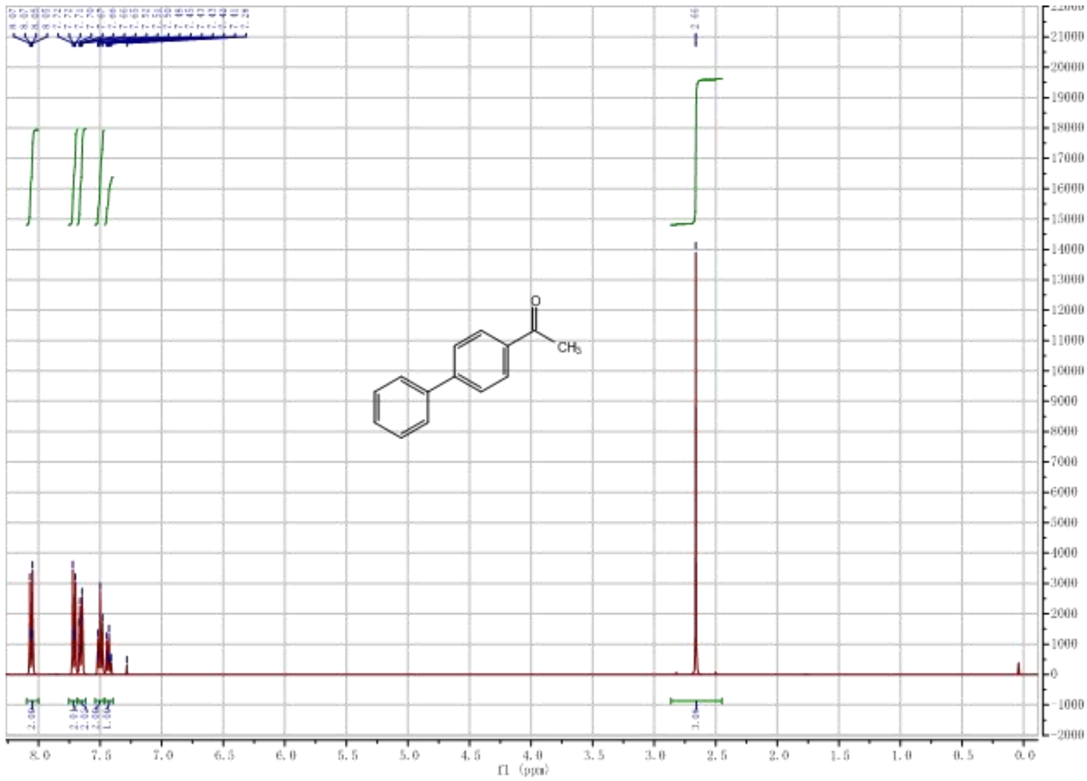
O
CH3
f1 (ppm)

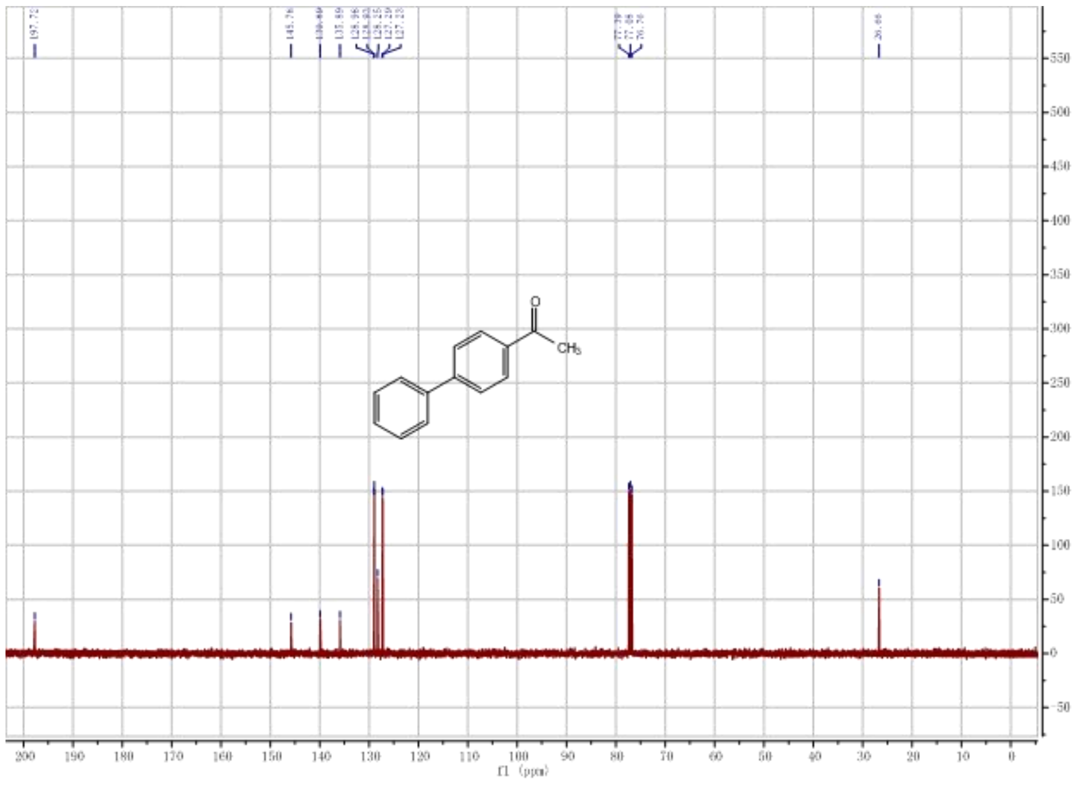
197.72
O
CH3
f1 (ppm)

GC-MS trace of the catalytic results for the selective oxidation of alkylbenzenes:

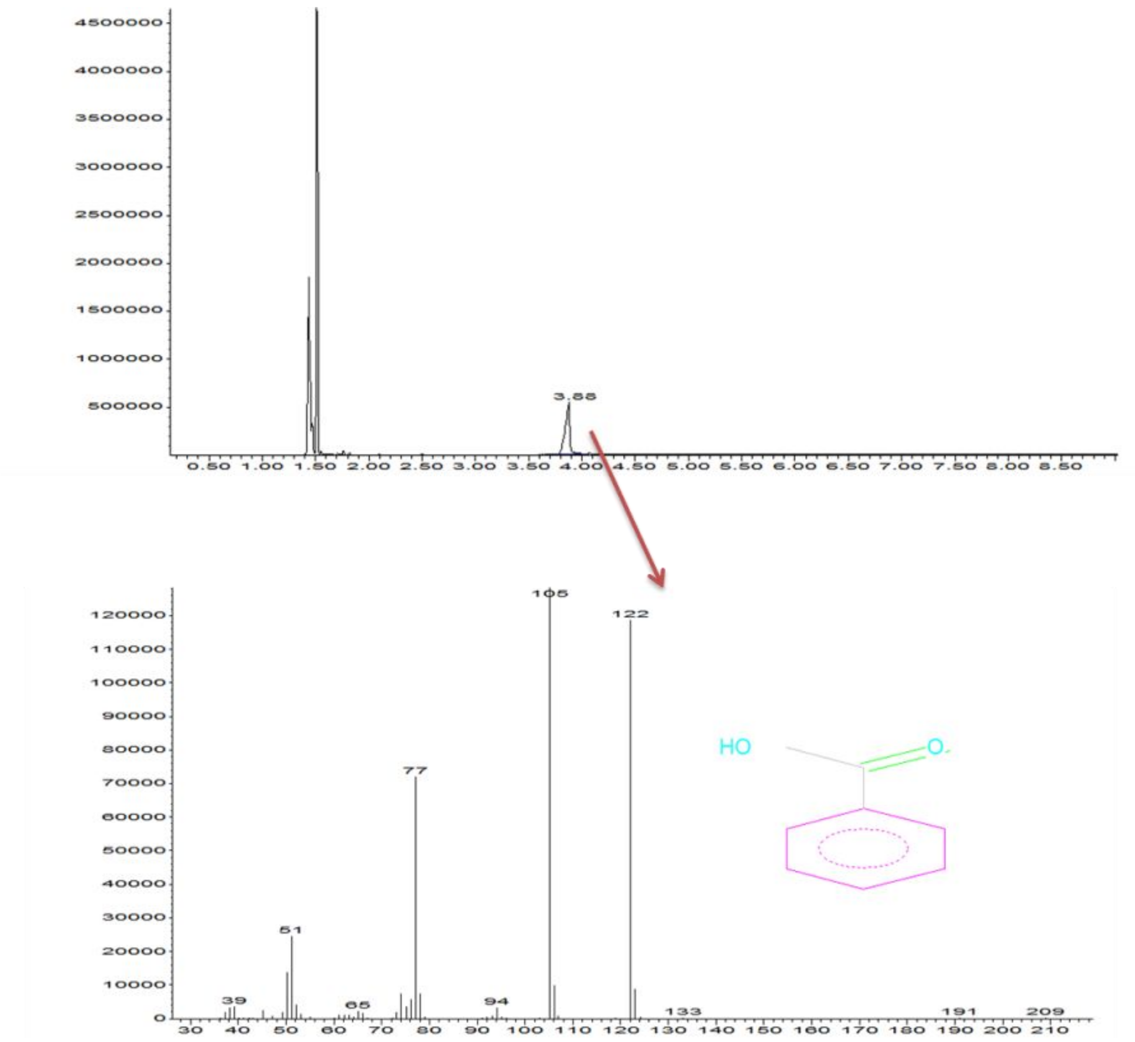

Figure S18. GC trace of the catalytic results for the selective oxidation of benzoic acid catalyzed by HLJU-1 for 12 h(top); MS spectra for the product at 3.88 min(bottom).

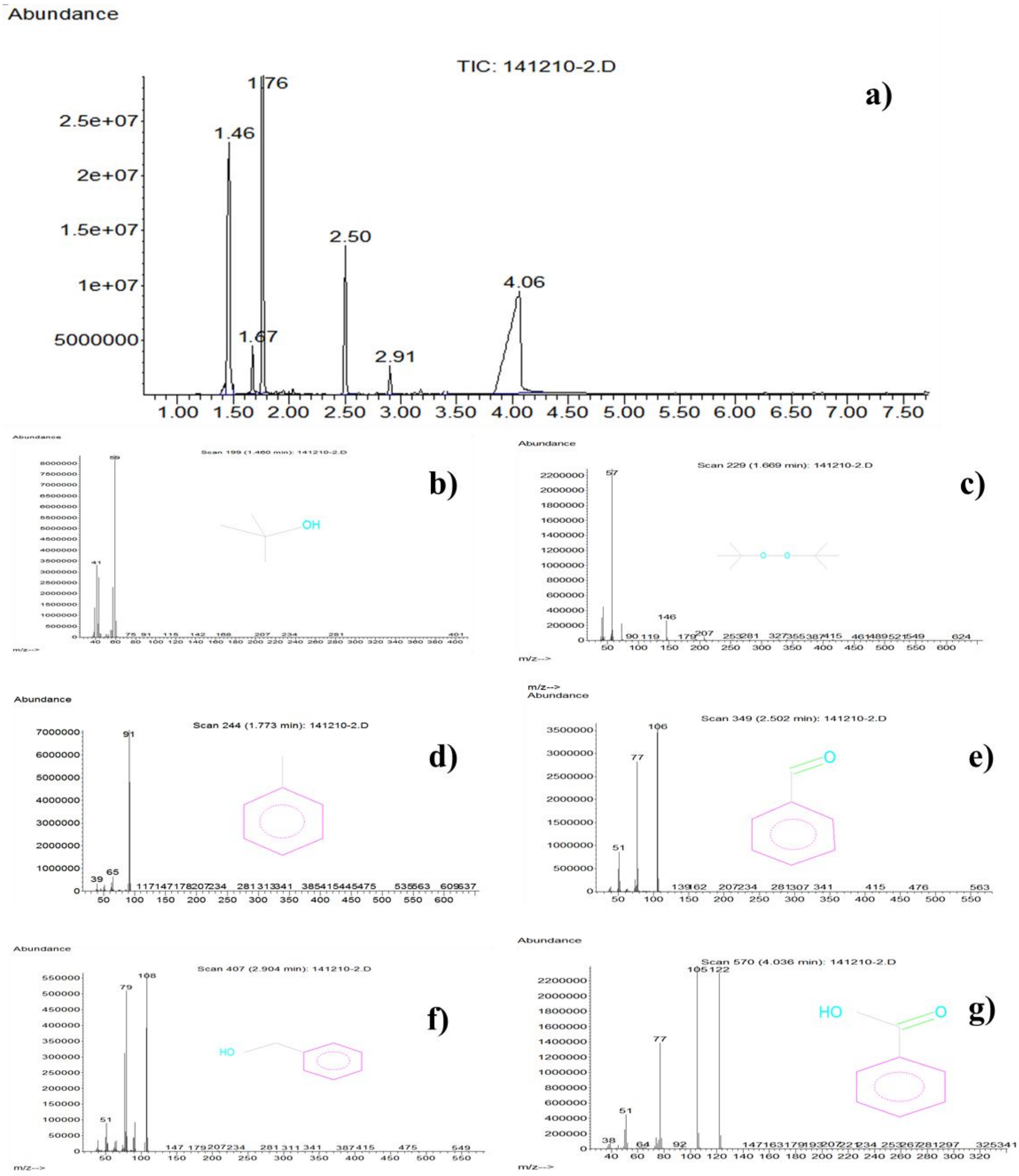

Figure S19. GC trace of the catalytic results for the selective oxidation of benzoic acid catalyzed by rht-MOF-1 for 12 h (a); MS spectra for the products at 1.46 min (b), 1.67 min (c), 1.76 min (d), 2.50 min (e), 2.91 min (f), 4.06 min (g).

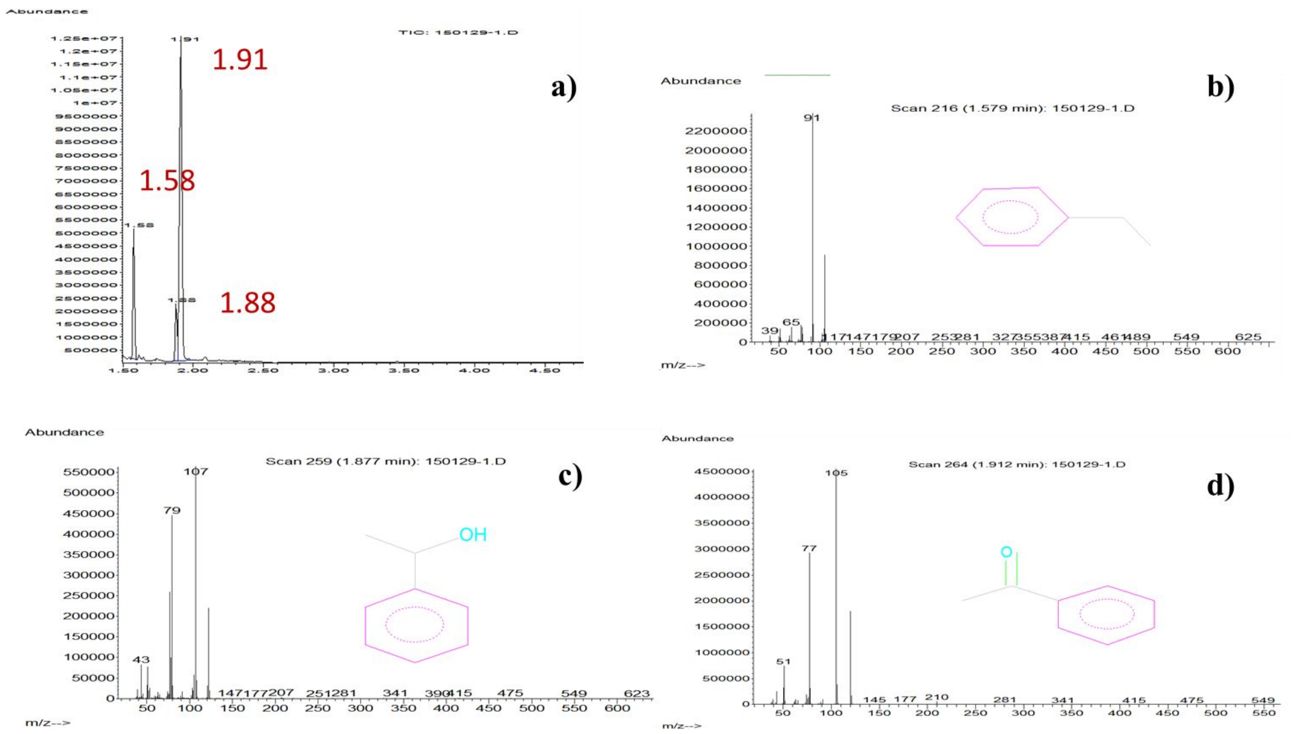

Figure S20. GC trace of the catalytic results for the selective oxidation of ethylbenzene catalyzed by HLJU-1 for 12 h (a); MS spectra for the products at 1.58 min (b), 1.91 min (c), 1.88 min (d).

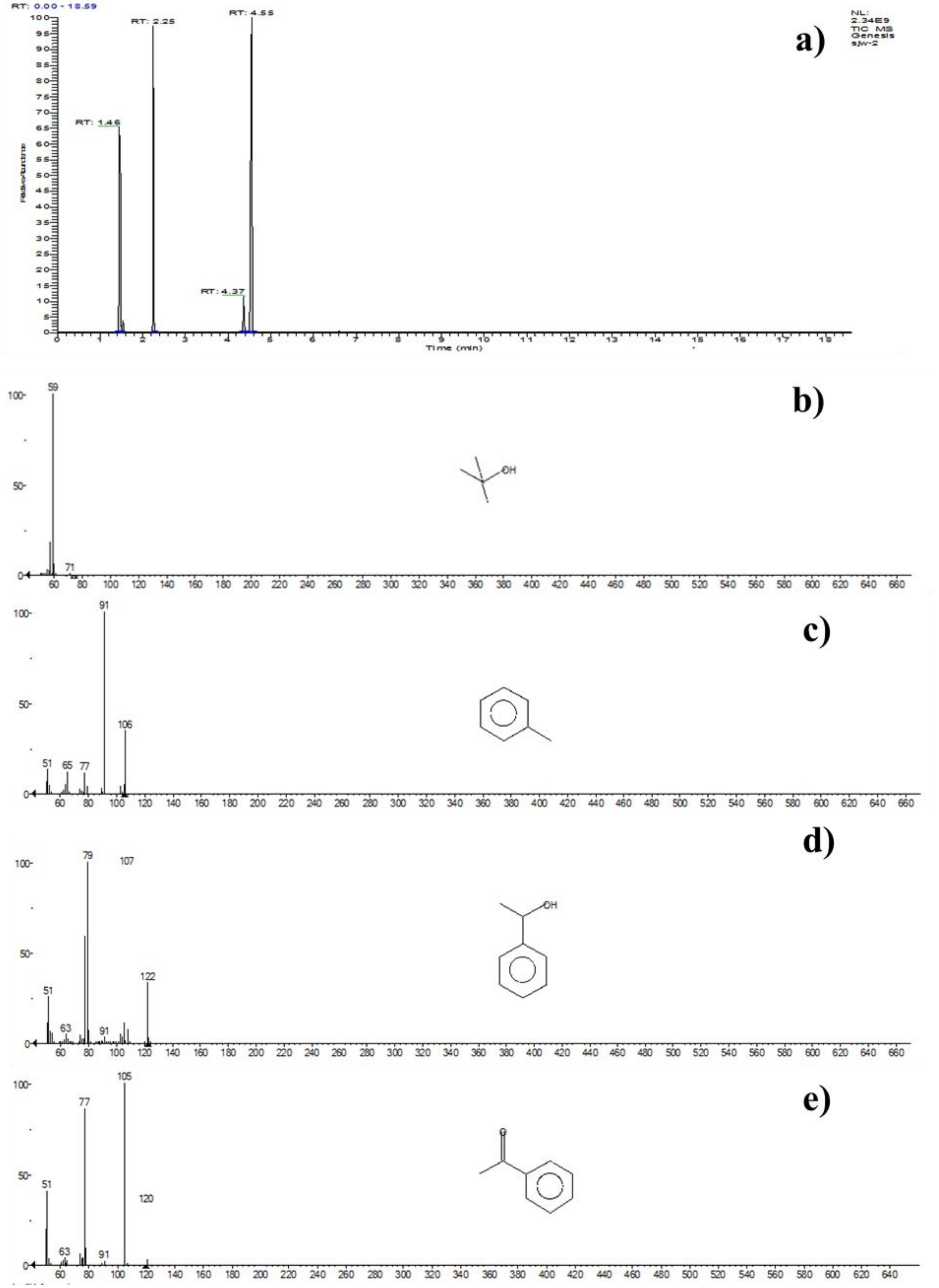

Figure S21. GC trace of the catalytic results for the selective oxidation of ethylbenzene catalyzed by HLJU-2 for 12 h (a); MS spectra for the products at 1.46 min (b), 2.25 min (c), 4.37 min (d) , 4.55 min (d).

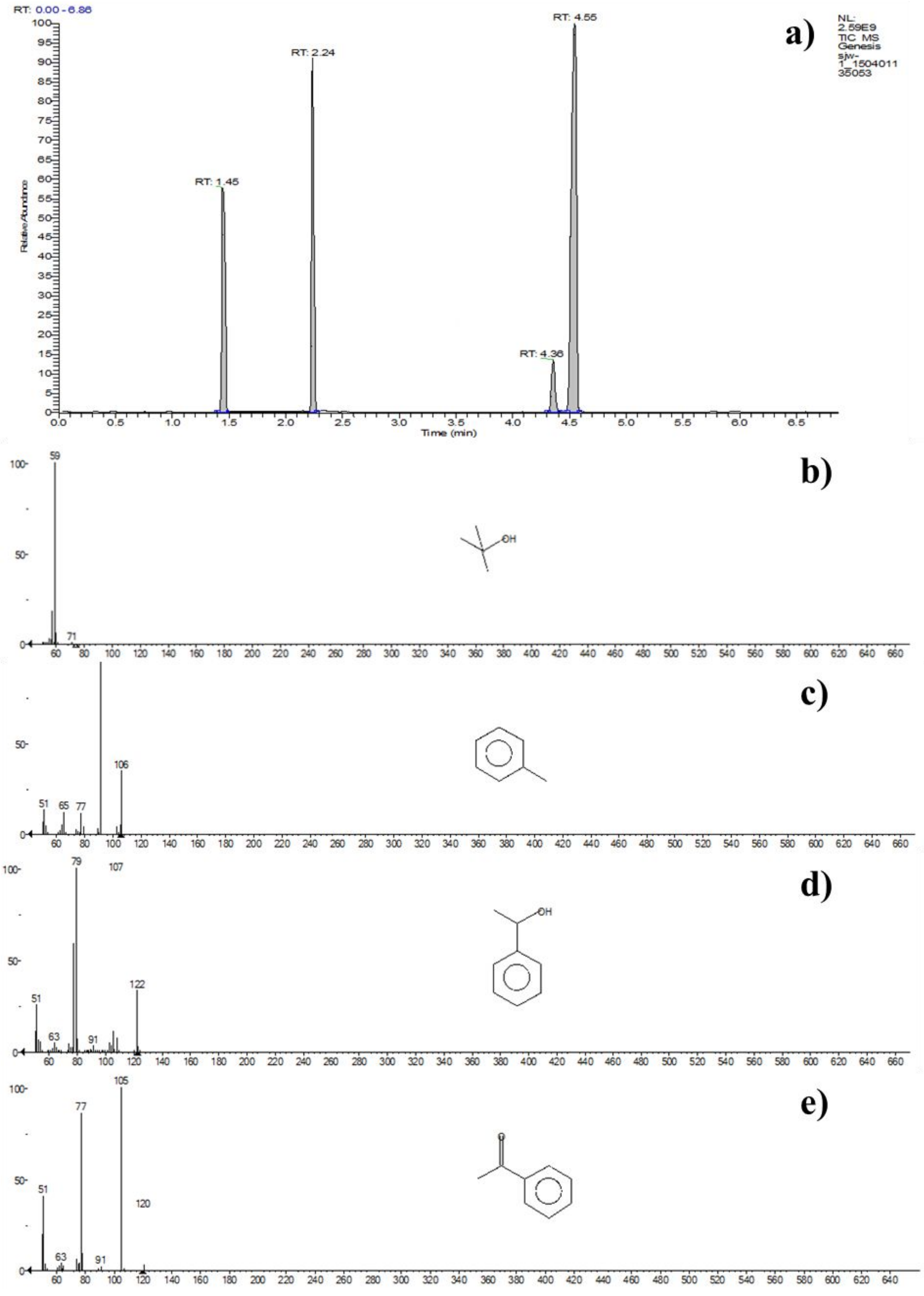

Figure S22. GC trace of the catalytic results for the selective oxidation of ethylbenzene catalyzed by HLJU-3 for 12 h (a); MS spectra for the products at 1.45 min (b), 2.24 min (c), 4.36 min (d) , 4.55 min (d).

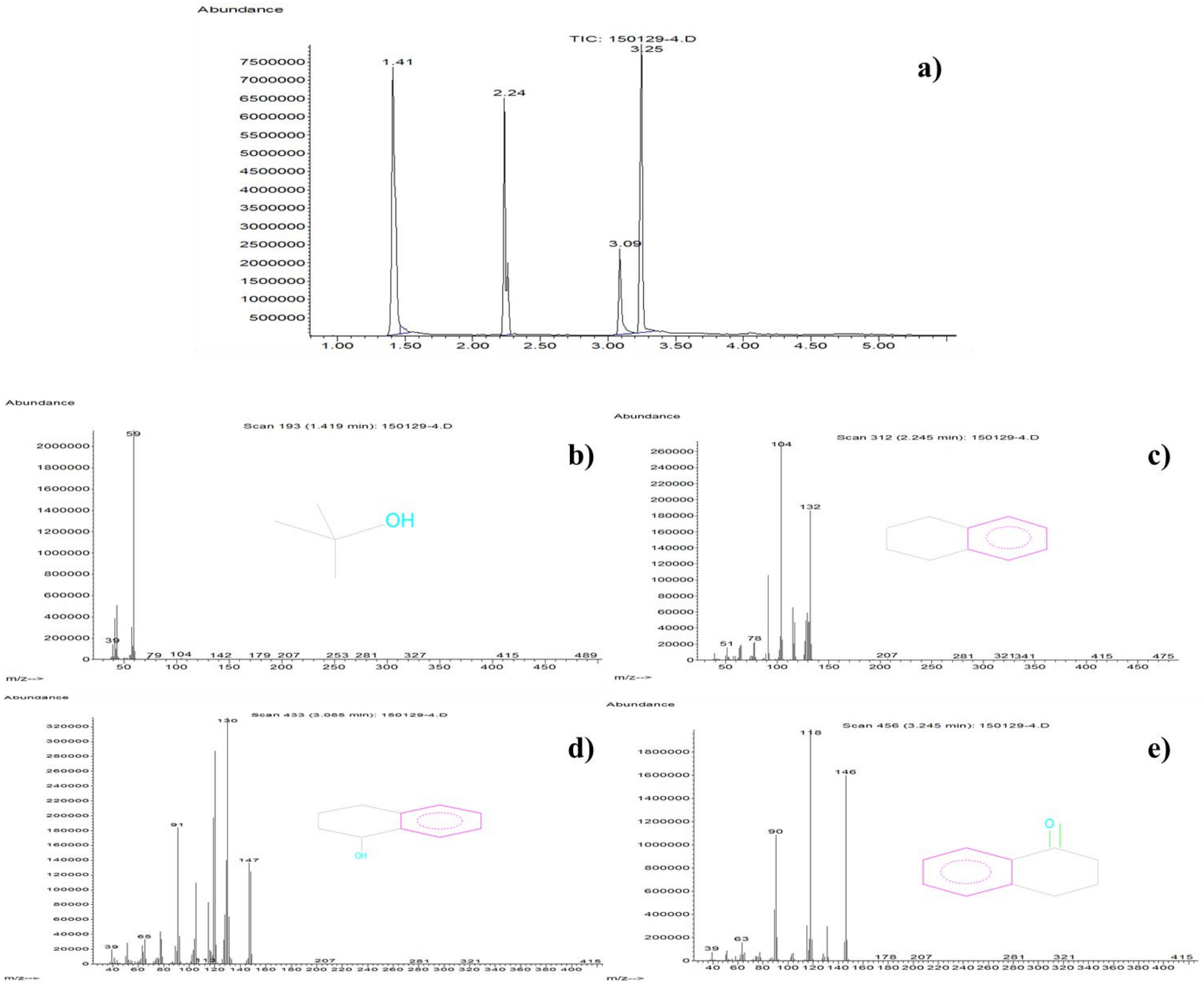

Figure S23. GC trace of the catalytic results for the selective oxidation of tetrahydronaphthalene catalyzed by HLJU-1 for 12 h (a); MS spectra for the products at 1.41 min (b), 2.24 min (c), 3.09 min (d), 3.25 min (e).

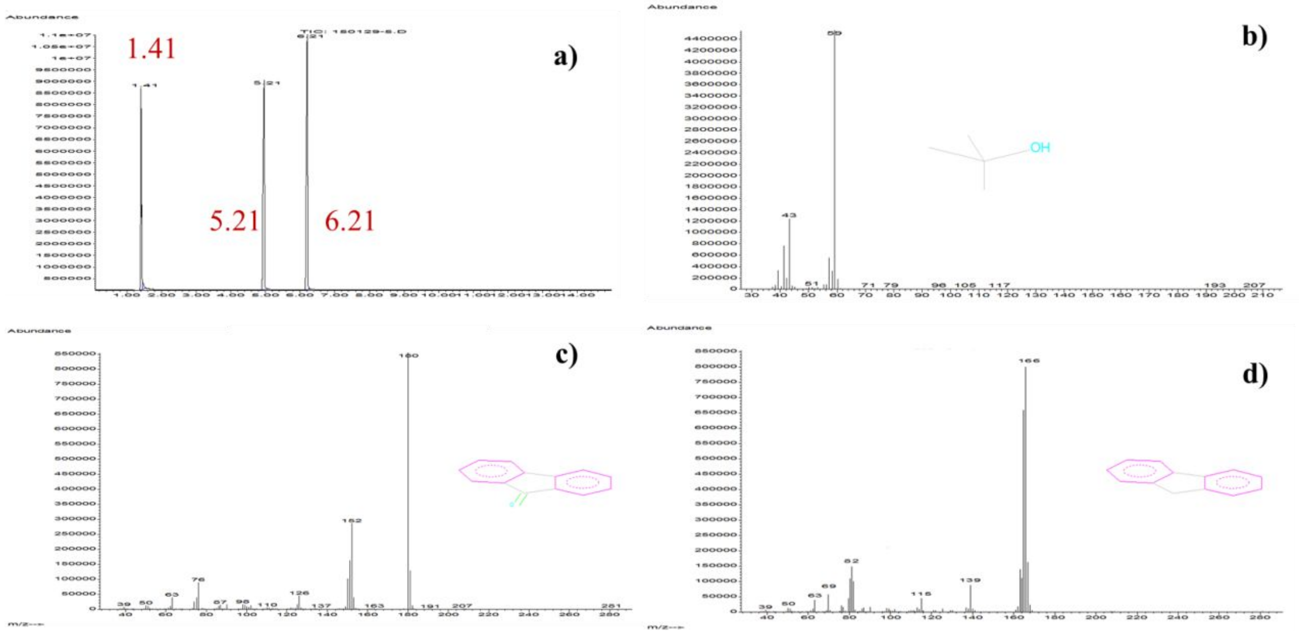

Figure S24. GC trace of the catalytic results for the selective oxidation of fluorene catalyzed by HLJU-1 for 12 h (a); MS spectra for the products at 1.41 min (b), 5.21 min (c), 6.21 min (d).

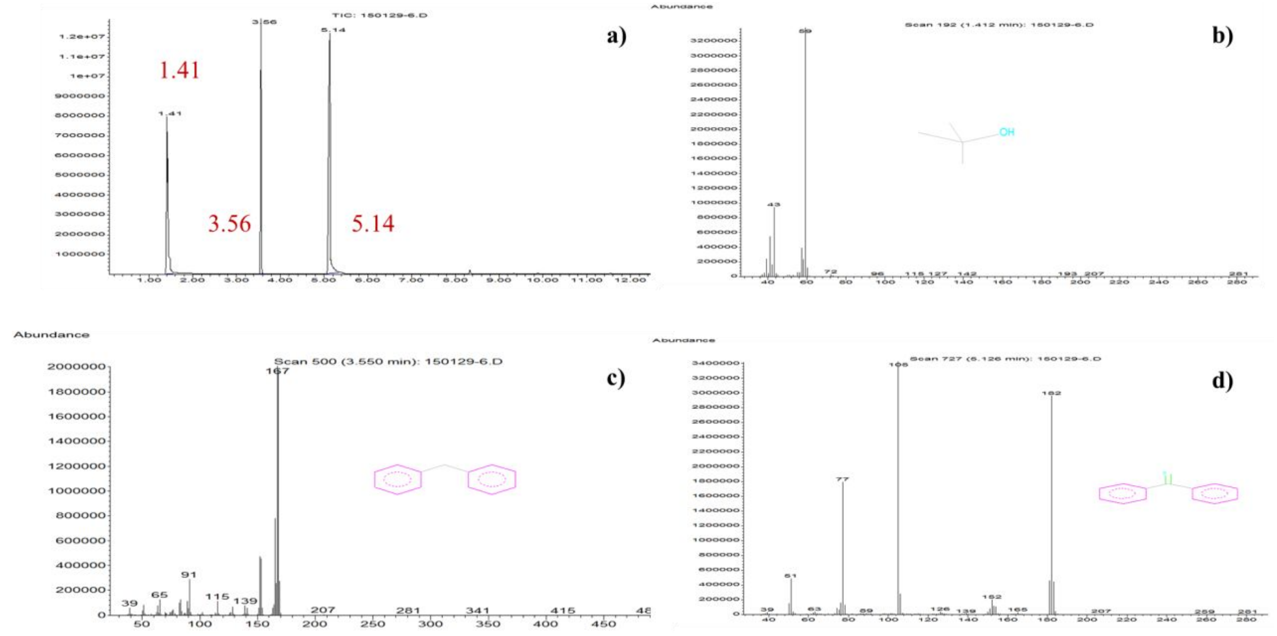

Figure S25. GC trace of the catalytic results for the selective oxidation of diphenylmethane catalyzed by HLJU-1 for 12 h (a); MS spectra for the products at 1.41 min (b), 3.56 min (c), 5.14 min (d).

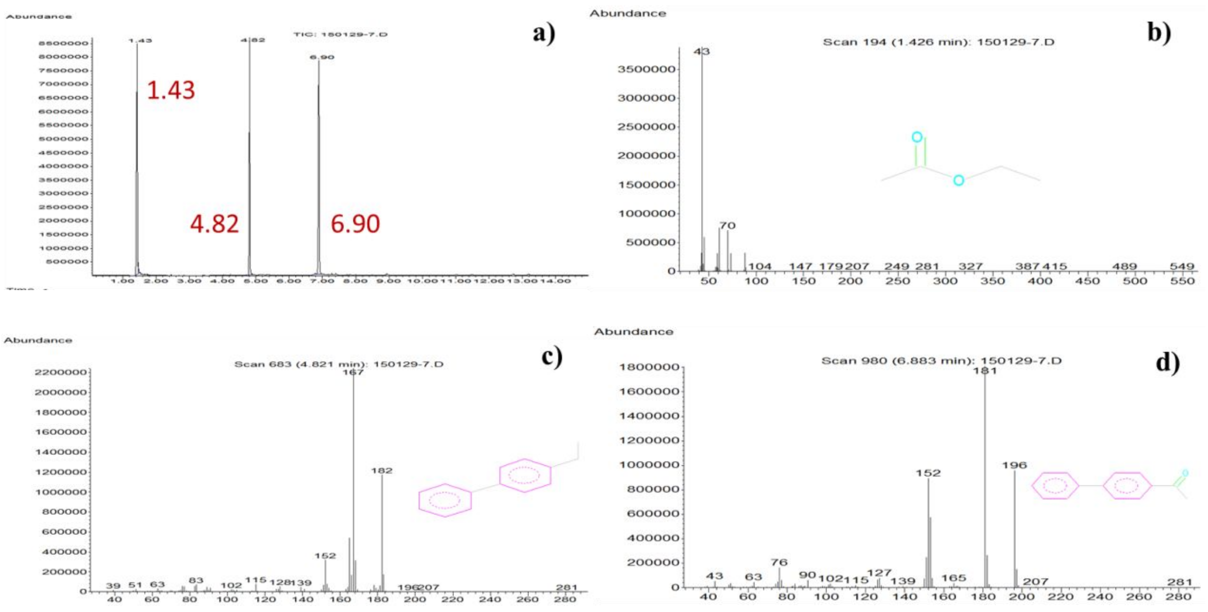

Figure S26. GC trace of the catalytic results for the selective oxidation of 4-ethyl-1,1'-biphenyl catalyzed by HLJU-1 for 12 h (a); MS spectra for the products at 1.43 min (b), 4.82 min (c), 6.90 min (d).

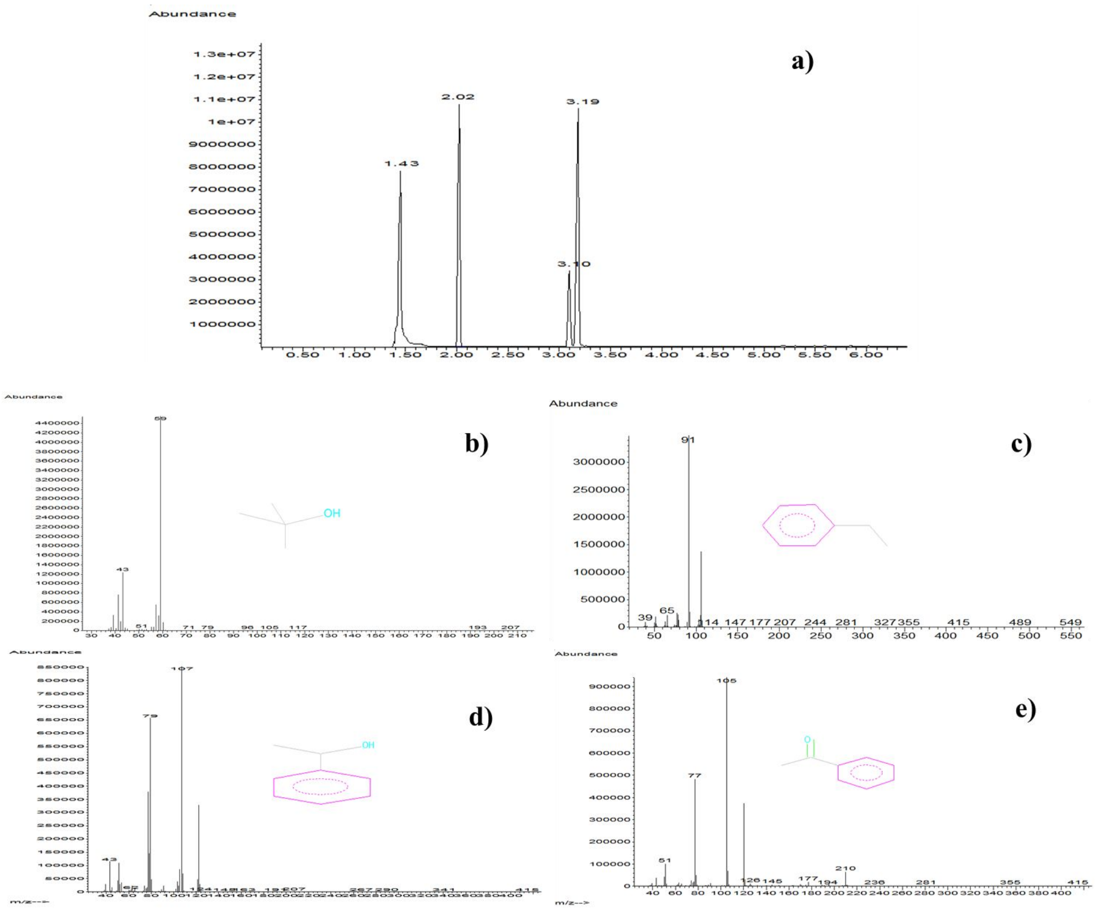

Figure S27. GC trace of the catalytic results for the selective oxidation of ethylbenzene catalyzed by HLJU-1 at fifth cycle(a); MS spectra for the products at 1.43 min (b), 2.02 min (c), 3.10 min (d) , 3.19 min (e).

^{1}H NMR experiments: A sample of HLJU-1 was heated under vacuum at 100 °C for 12 h. The sample was subsequently submersed in $CDCl_3$ for 12 h. The ^{1}H NMR analysis of the supernatant liquid suggests that the whole frameworks is stable (see Figure S28a). After a solvent-free sample HLJU-1 were immersed in ethylbenzene and TBHP for 12 h at room temperature, respectively, the solid were separated by filtration, and thoroughly washed with ethyl ether to remove surface adsorbed molecules. The solid was subsequently submersed in $CDCl_3$ for 12 h. The supernatant liquid was used for ^{1}H NMR study. The ethylbenzene and TBHP peaks are clearly visible from the ^{1}H NMR spectrum (see Figure S28b and c).

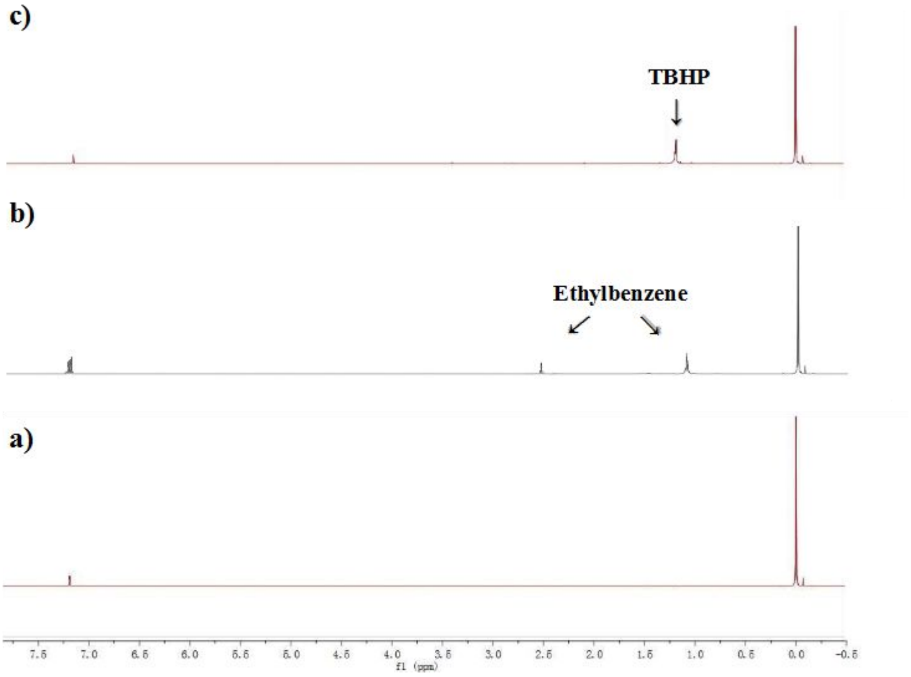

Figure S28. ^{1}H NMR spectra for the samples of HLJU-1: a) dried sample; b) after exposure to ethylbenzene; c) after exposure to TBHP.

Determination of adsorbed amount of ethylbenzene and TBHP by GC-MS: We have performed additional experiments to confirm that the catalytic reactions do occur in the void space (in addition to the solid surface). The adsorbed amount of ethylbenzene and TBHP were determined by GC-MS. After a solvent free sample HLJU-1 were immersed in ethylbenzene and TBHP for 6 h at room temperature, respectively, the solid were filtered and washed thoroughly with ethyl ether to remove surface adsorbed molecules. The solid were digested by dilute aqueous hydrochloric acid, which was then extracted with hexane solvent. The GC-MS results, by use of an external standard method, suggest that about 11.7 ethylbenzene molecules and 15 TBHP were adsorbed per formula unit of **1**.

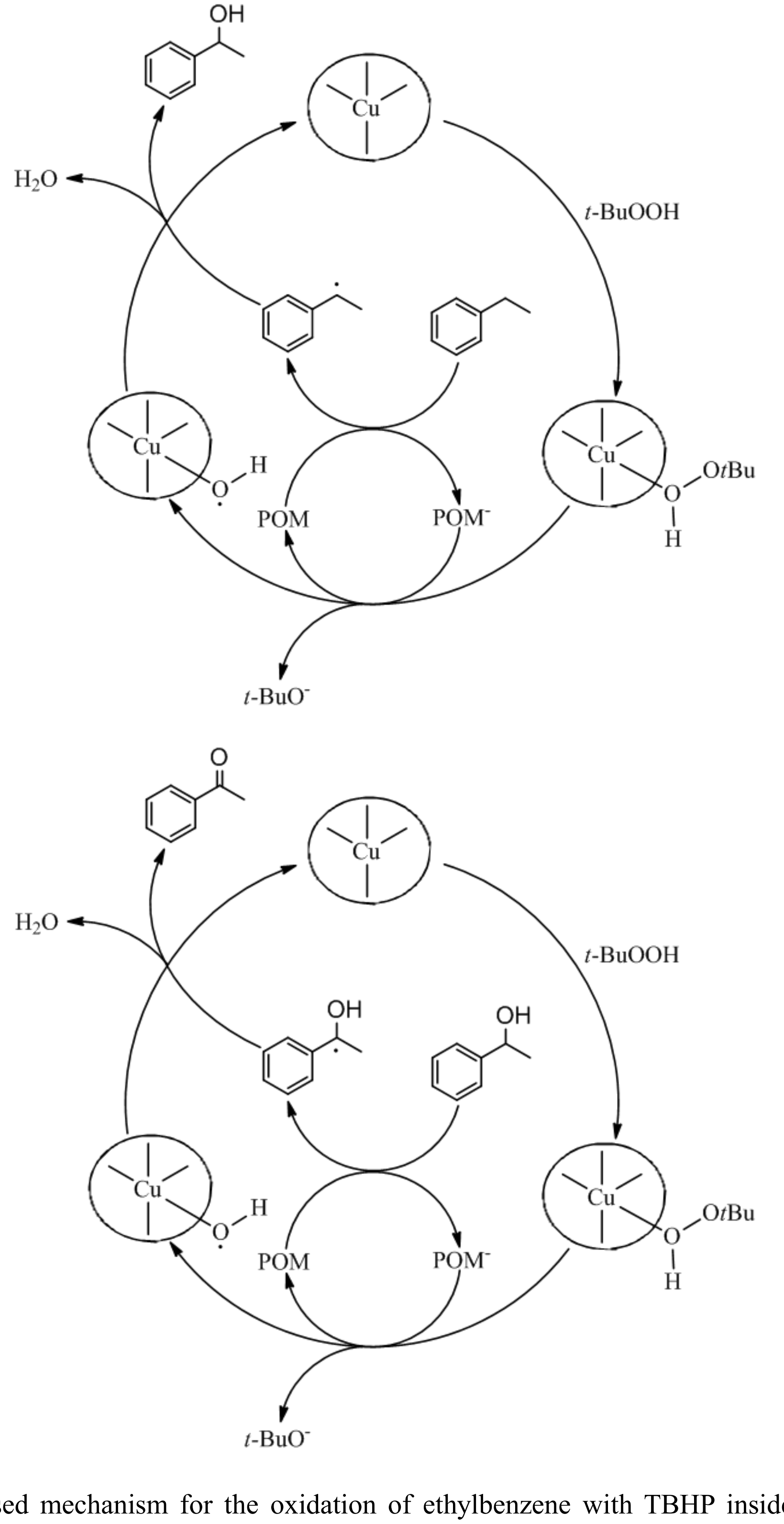

Scheme S1. Proposed mechanism for the oxidation of ethylbenzene with TBHP inside pores of HLJU-1 catalyst.

X-Ray Structures Analysis. Crystal dates of complexes HLJU 1–3 were selected on an Oxford Xcalibur Gemini Ultra diffractometer using graphite-monochromated Mo-Kα radiation (λ = 0.71073 Å) at room temperature. Structures of HLJU 1−3 were solved using Patterson methods (SHELXS-97), expanded using Fourier methods and refined using SHELXL-97 (full-matrix least-squares on F^2) and WinGX v1.70.01 programs packages.[1]All non-hydrogen atoms were refined anisotropically in HLJU-1 and HLJU-2. Due to the crystal quality of HLJU-3 is poor. The data refined by anisotropic for HLJU-3 is not stable. Therefore, the light atoms are refined by isotropic. Empirical absorption corrections based on equivalent reflections were applied. The contribution of HIGHLY disordered anions/solvent molecules were treated as diffuse using the Squeeze procedure implemented in the Platon program.[2] The resulting new files were used to further refine the structures. The Squeeze results are consistent with TG and elemental analysis, which indicate that there are 32 water molecules as free guests. These water molecules are added in the molecular formula. The HLJU-1 and HLJU-3 are electronegative, thus one water molecule is assigned as a hydroxyl to balance the charge. The crystal data and structure refinements of complexes HLJU 1–3 are summarized in Table S1. Selected bond lengths and angles for complexes HLJU 1–3 are listed in Tables S2–4.

Table S1 Crystal Data and Structure Refinements for HLJU 1−3

	HLJU-1	HLJU-2	HLJU-3
Empirical formula	$C_{108}H_{171}N_{48}Cu_{24}PMo_{12}O_{160}$	$C_{108}H_{172}N_{48}Cu_{24}SiMo_{12}O_{160}$	$C_{108}H_{147}N_{48}Cu_{24}PW_{12}O_{148}$
CCDC	1058671	1058599	1058600
Formula weight	7409.10	7407.22	8247.72
Crystal system	cubic	cubic	cubic
space group	$Fm\bar{3}m$	$Fm\bar{3}m$	$Fm\bar{3}m$
Unit cell	a = 44.599(3) Å<br>α = 90°	a = 44.614(5) Å<br>α = 90°	a = 4.4742(7) Å<br>α = 90°
Volume	88712(18) Å^3	88800(30) Å^3	87968(4) Å^3
Z	8	8	8
Density (calcd)	1.013 g·cm^{-3}	1.011 g·cm^{-3}	1.166 g·cm^{-3}
Temperature	293(2) K	293(2) K	293(2) K
Wavelength	0.71073 Å	0.71073 Å	0.71073 Å
Reflections collected	18345	16911	13509
μ	1.507 mm^{-1}	1.504 mm^{-1}	4.312 mm^{-1}
F(000)	26072.0	26064.0	28760.0
final$R1^a$, $wR2^b$ [$I > 2\sigma(I)$]	0.0875, 0.2584	0.0710, 0.2017	0.0994, 0.2314
final$R1^a$, $wR2^b$ (all data)	0.0997, 0.2696	0.0967, 0.2188	0.1302, 0.2547
GOF on F^2	1.041	0.990	0.853

$^aR_1 = \Sigma||F_0|-|F_c||/\Sigma|F_0|$. $^b wR_2 = \Sigma[w(F_0^2 - F_c^2)^2]/\ \Sigma[w(F_0^2)^2]^{1/2}$.

The unit cell of complexes HLJU 1-3 contain 32 water molecules which has been treated as a diffuse contribution to the overall scattering without specific atom positions by SQUEEZE/PLATON, respectively. The SQUEEZE results consistent with TG and elemental analysis. These water molecules have been included in the formula for the calculation of intensive properties. During the refinement, the oxygen atoms of HLJU-3 were refined isotropy due to the poor data of crystal. Large anisotropic displacement parameters (ADP's) for Cu3 and Mo(W) atoms can be explained bibration perpendicular to plane formed by these atoms. The charges of the frameworks of HLJU-1 and HLJU-3 are positive (+1). Therefore one water molecule is deprotonated to balance the charge of the POM@MOFs.

Table S2. Selected bond lengths [Å] and angles [deg] for HLJU-1

Cu(1)-O(2)#1	1.958(4)	Cu(3)-N(2)#4	1.959(5)
Cu(1)-O(2)	1.958(4)	Cu(3)-N(2)	1.959(5)
Cu(1)-O(2)#2	1.958(4)	Cu(3)-O(9B)	1.97(3)
Cu(1)-O(2)#3	1.958(4)	Cu(3)-O(3)	2.000(4)
Cu(1)-O(1W)	2.153(8)	Cu(3)-O(9A)	2.14(3)
Cu(2)-O(1)	1.957(4)	Cu(3)-O(10B)	2.26(4)
Cu(2)-O(1)#1	1.957(4)	O(3)-Cu(3)#5	2.000(4)
Cu(2)-O(1)#3	1.957(4)	O(3)-Cu(3)#6	2.000(4)
Cu(2)-O(1)#2	1.957(4)	Cu(2)-O(2W)	2.166(9)
O(2)-Cu(1)-O(2)#2	167.6(2)	O(2)#1-Cu(1)-O(2)	89.9(3)
O(2)#1-Cu(1)-O(2)#3	167.6(2)	O(2)#1-Cu(1)-O(2)#2	88.8(3)
O(2)-Cu(1)-O(2)#3	88.8(3)	O(2)#1-Cu(1)-O(1W)	96.21(11)
O(2)#2-Cu(1)-O(2)#3	89.9(3)	O(2)-Cu(1)-O(1W)	96.21(11)
O(2)#2-Cu(1)-O(1W)	96.21(11)	N(2)#4-Cu(3)-O(10B)	91.8(3)
O(2)#3-Cu(1)-O(1W)	96.21(11)	N(2)-Cu(3)-O(10B)	91.8(3)
O(2)#1-Cu(1)-Cu(2)	83.79(11)	O(9B)-Cu(3)-O(10B)	70.9(13)
O(2)-Cu(1)-Cu(2)	83.79(11)	O(3)-Cu(3)-O(10B)	114.9(12)
O(2)#2-Cu(1)-Cu(2)	83.79(11)	O(9A)-Cu(3)-O(10B)	107.8(13)
O(2)#3-Cu(1)-Cu(2)	83.79(11)	O(1)-Cu(2)-O(1)#1	90.2(3)
O(1W)-Cu(1)-Cu(2)	180.0(2)	O(1)-Cu(2)-O(1)#3	88.6(3)
O(1)-Cu(2)-O(1)#2	168.3(2)	N(2)#4-Cu(3)-O(10B)	91.8(3)
O(1)#1-Cu(2)-O(1)#2	88.6(3)	N(2)-Cu(3)-O(10B)	91.8(3)
O(1)#3-Cu(2)-O(1)#2	90.2(3)	N(2)-Cu(3)-O(3)	87.71(17)
O(1)-Cu(2)-O(2W)	95.86(11)	O(9B)-Cu(3)-O(3)	174.2(10)
O(1)#1-Cu(2)-O(2W)	95.86(11)	N(2)#4-Cu(3)-O(9A)	91.1(2)
O(1)#3-Cu(2)-O(2W)	95.86(11)	N(2)-Cu(3)-O(9A)	91.1(2)
O(1)#2-Cu(2)-O(2W)	95.86(11)	O(9B)-Cu(3)-O(9A)	36.9(10)
O(1)-Cu(2)-Cu(1)	84.14(11)	O(3)-Cu(3)-O(9A)	137.3(8)
O(1)#1-Cu(2)-Cu(1)	84.14(11)	N(2)#4-Cu(3)-N(2)	175.1(4)
O(1)#3-Cu(2)-Cu(1)	84.14(11)	N(2)#4-Cu(3)-O(9B)	92.18(16)
O(1)#2-Cu(2)-Cu(1)	84.14(11)	N(2)-Cu(3)-O(9B)	92.18(16)
O(2W)-Cu(2)-Cu(1)	180.0(2)	N(2)#4-Cu(3)-O(3)	87.71(17)

Symmetry transformations used to generate equivalent atoms: #1 x, y, -z; #2 y, x, -z; #3 y, x, z; #4 -z+1/2, y, -x+1/2; #5 -y+1/2, z, -x+1/2; #6 -z+1/2, -x+1/2, y.

Table S3. Selected bond lengths [Å] and angles [deg] for HLJU-2

Cu(2)-O(5)#1	1.953(4)	Cu(1)-O(3)#3	1.952(4)
Cu(2)-O(5)	1.953(4)	Cu(1)-O(3)#2	1.952(4)
Cu(2)-O(5)#2	1.953(4)	Cu(1)-O(2)	2.142(9)
Cu(2)-O(5)#3	1.953(4)	Cu(3)-N(1)#4	1.967(5)
Cu(2)-O(1)	2.171(8)	Cu(3)-N(1)	1.967(5)
Cu(2)-Cu(1)	2.6445(15)	Cu(3)-O(6)	1.978(13)
Cu(1)-O(3)#1	1.952(4)	Cu(3)-O(12)	2.012(4)
Cu(1)-O(3)	1.952(4)	Cu(3)-O(7)	2.20(2)
O(5)#1-Cu(2)-O(5)	89.7(2)	O(5)#2-Cu(2)-O(1)	95.95(11)
O(5)#1-Cu(2)-O(5)#2	168.1(2)	O(5)#3-Cu(2)-O(1)	95.95(11)
O(5)-Cu(2)-O(5)#2	89.0(2)	O(5)#1-Cu(2)-Cu(1)	84.05(11)
O(5)#1-Cu(2)-O(5)#3	89.0(2)	O(5)-Cu(2)-Cu(1)	84.05(11)
O(5)-Cu(2)-O(5)#3	168.1(2)	O(5)#2-Cu(2)-Cu(1)	84.05(11)
O(5)#2-Cu(2)-O(5)#3	89.7(2)	O(5)#3-Cu(2)-Cu(1)	84.05(11)
O(5)#1-Cu(2)-O(1)	95.95(11)	O(1)-Cu(2)-Cu(1)	180.0(2)
O(5)-Cu(2)-O(1)	95.95(11)	O(3)#3-Cu(1)-O(2)	95.95(11)
O(3)#1-Cu(1)-O(3)	89.7(2)	O(3)#2-Cu(1)-O(2)	95.95(11)
O(3)#1-Cu(1)-O(3)#3	89.1(2)	O(3)#1-Cu(1)-Cu(2)	84.05(11)
O(3)-Cu(1)-O(3)#3	168.1(2)	O(3)-Cu(1)-Cu(2)	84.05(11)
O(3)#1-Cu(1)-O(3)#2	168.1(2)	O(3)#3-Cu(1)-Cu(2)	84.05(11)
O(3)-Cu(1)-O(3)#2	89.1(2)	O(3)#2-Cu(1)-Cu(2)	84.05(11)
O(3)#3-Cu(1)-O(3)#2	89.7(2)	O(2)-Cu(1)-Cu(2)	180.0(2)
O(3)#1-Cu(1)-O(2)	95.95(11)	O(3)-Cu(1)-O(2)	95.95(11)
N(1)#4-Cu(3)-N(1)	174.9(3)	N(1)#4-Cu(3)-O(7)	91.6(2)
N(1)#4-Cu(3)-O(6)	91.60(15)	N(1)-Cu(3)-O(7)	91.6(2)
N(1)-Cu(3)-O(6)	91.60(15)	O(6)-Cu(3)-O(7)	102.1(10)
N(1)#4-Cu(3)-O(12)	87.63(14)	O(12)-Cu(3)-O(7)	107.0(9)
N(1)-Cu(3)-O(12)	87.63(15)	O(6)-Cu(3)-O(12)	150.9(9)

Symmetry transformations used to generate equivalent atoms: #1 x, y, -z; #2 y, x, z; #3 y, x, -z; #4 -y+1/2, -x+1/2, z.

Table S4. Selected bond lengths [Å] and angles [deg] for HLJU-3

Cu(1)-O(5)#1	1.943(7)	Cu(2)-O(4)#3	1.957(7)
Cu(1)-O(5)	1.943(7)	Cu(2)-O(4)#1	1.957(7)
Cu(1)-O(5)#2	1.943(7)	Cu(2)-O(4)#2	1.957(7)
Cu(1)-O(5)#3	1.943(7)	Cu(2)-O(2)	2.20(4)
Cu(1)-O(3)	2.146(18)	Cu(3)-O(30)	1.70(6)
Cu(1)-Cu(2)	2.649(3)	Cu(3)-O(11)	1.931(4)
Cu(2)-O(4)	1.957(7)	Cu(3)-N(4)	1.942(13)
Cu(3)-N(4)#4	1.942(13)	O(2)-Cu(2)-Cu(1)	180.0(10)
O(30)-Cu(3)-O(11)	165(2)	O(11)-Cu(3)-N(4)	87.8(4)
O(30)-Cu(3)-N(4)	91.2(4)	O(30)-Cu(3)-N(4)#4	91.2(4)
O(5)#1-Cu(1)-O(5)	89.0(4)	O(11)-Cu(3)-N(4)#4	87.8(4)
O(5)#1-Cu(1)-O(5)#2	89.8(4)	N(4)-Cu(3)-N(4)#4	172.1(9)
O(5)-Cu(1)-O(5)#2	168.1(4)	O(4)#3-Cu(2)-O(4)#2	88.5(4)
O(5)#1-Cu(1)-O(5)#3	168.1(4)	O(4)#1-Cu(2)-O(4)#2	90.3(4)
O(5)-Cu(1)-O(5)#3	89.8(4)	O(4)-Cu(2)-O(2)	95.72(19)
O(5)#2-Cu(1)-O(5)#3	89.0(4)	O(4)#3-Cu(2)-O(2)	95.72(19)
O(5)#1-Cu(1)-O(3)	96.0(2)	O(4)#1-Cu(2)-O(2)	95.72(19)
O(5)-Cu(1)-O(3)	96.0(2)	O(4)#2-Cu(2)-O(2)	95.72(19)
O(5)#2-Cu(1)-O(3)	96.0(2)	O(4)-Cu(2)-Cu(1)	84.28(19)
O(5)#3-Cu(1)-O(3)	96.0(2)	O(4)#3-Cu(2)-Cu(1)	84.28(19)
O(5)#1-Cu(1)-Cu(2)	84.0(2)	O(4)#1-Cu(2)-Cu(1)	84.28(19)
O(5)-Cu(1)-Cu(2)	84.0(2)	O(4)#2-Cu(2)-Cu(1)	84.28(19)
O(5)#2-Cu(1)-Cu(2)	84.0(2)	O(4)-Cu(2)-O(4)#1	88.5(4)
O(5)#3-Cu(1)-Cu(2)	84.0(2)	O(4)#3-Cu(2)-O(4)#1	168.6(4)
O(3)-Cu(1)-Cu(2)	180.0(5)	O(4)-Cu(2)-O(4)#2	168.6(4)

Symmetry transformations used to generate equivalent atoms: #1 x, y, -z; #2 y, x, -z; #3 y, x, z; #4 -y+1/2, -x+1/2, z.

References

[1] a) G. M. Sheldrick, *Program for Structure Refinement*: University of Göttingen, Germany, **1997**; b) L. Farrugia, *J. Appl. Cryst.* **1999**, *32*, 837-838; c) G. M. Sheldrick, *SHELXL-97* (**1997**) Program for the Refinement of Crystal; d) G. M. Sheldrick, *Acta Cryst.* **1990**, *A46*, 467-473; e) G. M. Sheldrick, *Acta Cryst.* **2008**, *A64*, 112-122.

[2] H. Furukawa, T. Nakamura, H. Inagaki, E. Nishikawa, C. Imai, M. Misono, *Chem. Lett.* **1988**, 877-880.

checkCIF/PLATON report

You have not supplied any structure factors. As a result the full set of tests cannot be run.

THIS REPORT IS FOR GUIDANCE ONLY. IF USED AS PART OF A REVIEW PROCEDURE FOR PUBLICATION, IT SHOULD NOT REPLACE THE EXPERTISE OF AN EXPERIENCED CRYSTALLOGRAPHIC REFEREE.

No syntax errors found. CIF dictionary Interpreting this report

Datablock: HLJU-1

Bond precision:	C-C = 0.0098 A		Wavelength=0.71073
Cell:	a=44.5993(3)	b=44.5993(3)	c=44.5993(3)
	alpha=90	beta=90	gamma=90
Temperature:	293 K		

	Calculated	Reported
Volume	88712.4(18)	88712.4(18)
Space group	F m -3 m	F m -3 m
Hall group	-F 4 2 3	-F 4 2 3
Moiety formula	4(C27 H9 Cu6 N12 O22), Mo12 O40 P	4(C27 H9 Cu6 N12 O22), Mo12 O40 P
Sum formula	C108 H36 Cu24 Mo12 N48 O128 P	C108 H36 Cu24 Mo12 N48 O128 P
Mr	6761.30	6761.30
Dx,g cm-3	1.013	1.013
Z	8	8
Mu (mm-1)	1.507	1.507
F000	26072.0	26072.0
F000’	25980.38	
h,k,lmax	53,53,53	52,43,46
Nref	3810	3810
Tmin,Tmax	0.747,0.740	0.740,0.747
Tmin’	0.732	

Correction method= # Reported T Limits: Tmin=0.740 Tmax=0.747
AbsCorr = MULTI-SCAN

Data completeness= 1.000 Theta(max)= 24.990

R(reflections)= 0.0875(3026) wR2(reflections)= 0.2696(3810)

S = 1.041 Npar= 148

```
The following ALERTS were generated. Each ALERT has the format
       test-name_ALERT_alert-type_alert-level.
Click on the hyperlinks for more details of the test.
```

Alert level B

```
PLAT031_ALERT_4_B Refined Extinction Parameter within Range ......      2.222 Sigma
PLAT213_ALERT_2_B Atom O2W             has ADP max/min Ratio .....        4.8 prolat
PLAT220_ALERT_2_B Large Non-Solvent  O     Ueq(max)/Ueq(min) Range        6.1 Ratio
PLAT230_ALERT_2_B Hirshfeld Test Diff for    P1     --  O4      ..        7.6 su
PLAT242_ALERT_2_B Low       Ueq as Compared to Neighbors for .....        Cu2 Check
```

Alert level C

```
RFACR01_ALERT_3_C  The value of the weighted R factor is > 0.25
            Weighted R factor given   0.270
PLAT084_ALERT_3_C High wR2 Value (i.e. > 0.25) ...................       0.27 Report
PLAT213_ALERT_2_C Atom Cu3             has ADP max/min Ratio .....        3.1 prolat
PLAT213_ALERT_2_C Atom O1W             has ADP max/min Ratio .....        3.1 oblate
PLAT220_ALERT_2_C Large Non-Solvent Cu     Ueq(max)/Ueq(min) Range        4.3 Ratio
PLAT241_ALERT_2_C High      Ueq as Compared to Neighbors for .....         O4 Check
PLAT242_ALERT_2_C Low       Ueq as Compared to Neighbors for .....        Cu1 Check
PLAT242_ALERT_2_C Low       Ueq as Compared to Neighbors for .....        Cu3 Check
PLAT250_ALERT_2_C Large U3/U1 Ratio for Average U(i,j) Tensor ....        4.0 Note
PLAT342_ALERT_3_C Low Bond Precision on  C-C Bonds ...............     0.0098 Ang.
PLAT732_ALERT_1_C Angle   Calc    120.3(7), Rep    120.3(3) ......       2.33 su-Rat
              C3   -C2   -C1      1.555   1.555   1.555      #         56
```

Alert level G

```
PLAT004_ALERT_5_G Polymeric Structure Found with Maximum Dimension          3 Info
PLAT005_ALERT_5_G No _iucr_refine_instructions_details  in the CIF     Please Do !
PLAT072_ALERT_2_G SHELXL First  Parameter in WGHT Unusually Large.       0.18 Report
PLAT083_ALERT_2_G SHELXL Second Parameter in WGHT Unusually Large.     687.03 Why ?
PLAT199_ALERT_1_G Reported _cell_measurement_temperature ..... (K)        293 Check
PLAT200_ALERT_1_G Reported   _diffrn_ambient_temperature ..... (K)        293 Check
PLAT300_ALERT_4_G Atom Site Occupancy of *O9A    is Constrained at      0.500 Check
PLAT300_ALERT_4_G Atom Site Occupancy of *O9B    is Constrained at      0.500 Check
PLAT300_ALERT_4_G Atom Site Occupancy of *O10A   is Constrained at      0.500 Check
PLAT300_ALERT_4_G Atom Site Occupancy of *O10B   is Constrained at      0.500 Check
PLAT300_ALERT_4_G Atom Site Occupancy of *O9A_x  is Constrained at      0.500 Check
PLAT300_ALERT_4_G Atom Site Occupancy of *O9B_x  is Constrained at      0.500 Check
PLAT300_ALERT_4_G Atom Site Occupancy of *O10A_x is Constrained at      0.500 Check
PLAT300_ALERT_4_G Atom Site Occupancy of *O10B_x is Constrained at      0.500 Check
PLAT300_ALERT_4_G Atom Site Occupancy of *O9A_y  is Constrained at      0.500 Check
PLAT300_ALERT_4_G Atom Site Occupancy of *O9B_y  is Constrained at      0.500 Check
PLAT300_ALERT_4_G Atom Site Occupancy of *O10A_y is Constrained at      0.500 Check
PLAT300_ALERT_4_G Atom Site Occupancy of *O10B_y is Constrained at      0.500 Check
PLAT300_ALERT_4_G Atom Site Occupancy of *O9A_z  is Constrained at      0.500 Check
PLAT300_ALERT_4_G Atom Site Occupancy of *O9B_z  is Constrained at      0.500 Check
PLAT300_ALERT_4_G Atom Site Occupancy of *O10A_z is Constrained at      0.500 Check
PLAT300_ALERT_4_G Atom Site Occupancy of *O10B_z is Constrained at      0.500 Check
PLAT300_ALERT_4_G Atom Site Occupancy of *O9A_*  is Constrained at      0.500 Check
PLAT300_ALERT_4_G Atom Site Occupancy of *O9B_*  is Constrained at      0.500 Check
PLAT300_ALERT_4_G Atom Site Occupancy of *O10A_* is Constrained at      0.500 Check
PLAT300_ALERT_4_G Atom Site Occupancy of *O10B_* is Constrained at      0.500 Check
PLAT300_ALERT_4_G Atom Site Occupancy of *O9A_*  is Constrained at      0.500 Check
PLAT300_ALERT_4_G Atom Site Occupancy of *O9B_*  is Constrained at      0.500 Check
PLAT300_ALERT_4_G Atom Site Occupancy of *O10A_* is Constrained at      0.500 Check
PLAT300_ALERT_4_G Atom Site Occupancy of *O10B_* is Constrained at      0.500 Check
PLAT300_ALERT_4_G Atom Site Occupancy of *O9A_*  is Constrained at      0.500 Check
```

PLAT300_ALERT_4_G Atom Site Occupancy of *O9B_* is Constrained at   0.500 Check
PLAT300_ALERT_4_G Atom Site Occupancy of *O10A_* is Constrained at   0.500 Check
PLAT300_ALERT_4_G Atom Site Occupancy of *O10B_* is Constrained at   0.500 Check
PLAT300_ALERT_4_G Atom Site Occupancy of *O9A_* is Constrained at   0.500 Check
PLAT300_ALERT_4_G Atom Site Occupancy of *O9B_* is Constrained at   0.500 Check
PLAT300_ALERT_4_G Atom Site Occupancy of *O10A_* is Constrained at   0.500 Check
PLAT300_ALERT_4_G Atom Site Occupancy of *O10B_* is Constrained at   0.500 Check
PLAT300_ALERT_4_G Atom Site Occupancy of *O9A_* is Constrained at   0.500 Check
PLAT300_ALERT_4_G Atom Site Occupancy of *O9B_* is Constrained at   0.500 Check
PLAT300_ALERT_4_G Atom Site Occupancy of *O10A_* is Constrained at   0.500 Check
PLAT300_ALERT_4_G Atom Site Occupancy of *O10B_* is Constrained at   0.500 Check
PLAT300_ALERT_4_G Atom Site Occupancy of *O9A_* is Constrained at   0.500 Check
PLAT300_ALERT_4_G Atom Site Occupancy of *O9B_* is Constrained at   0.500 Check
PLAT300_ALERT_4_G Atom Site Occupancy of *O10A_* is Constrained at   0.500 Check
PLAT300_ALERT_4_G Atom Site Occupancy of *O10B_* is Constrained at   0.500 Check
PLAT300_ALERT_4_G Atom Site Occupancy of *O9A_* is Constrained at   0.500 Check
PLAT300_ALERT_4_G Atom Site Occupancy of *O9B_* is Constrained at   0.500 Check
PLAT300_ALERT_4_G Atom Site Occupancy of *O10A_* is Constrained at   0.500 Check
PLAT300_ALERT_4_G Atom Site Occupancy of *O10B_* is Constrained at   0.500 Check
PLAT300_ALERT_4_G Atom Site Occupancy of *O9A_* is Constrained at   0.500 Check
PLAT300_ALERT_4_G Atom Site Occupancy of *O9B_* is Constrained at   0.500 Check
PLAT300_ALERT_4_G Atom Site Occupancy of *O10A_* is Constrained at   0.500 Check
PLAT300_ALERT_4_G Atom Site Occupancy of *O10B_* is Constrained at   0.500 Check
PLAT300_ALERT_4_G Atom Site Occupancy of *O9A_* is Constrained at   0.500 Check
PLAT300_ALERT_4_G Atom Site Occupancy of *O9B_* is Constrained at   0.500 Check
PLAT300_ALERT_4_G Atom Site Occupancy of *O10A_* is Constrained at   0.500 Check
PLAT300_ALERT_4_G Atom Site Occupancy of *O10B_* is Constrained at   0.500 Check
PLAT300_ALERT_4_G Atom Site Occupancy of *O9A_* is Constrained at   0.500 Check
PLAT300_ALERT_4_G Atom Site Occupancy of *O9B_* is Constrained at   0.500 Check
PLAT300_ALERT_4_G Atom Site Occupancy of *O10A_* is Constrained at   0.500 Check
PLAT300_ALERT_4_G Atom Site Occupancy of *O10B_* is Constrained at   0.500 Check
PLAT300_ALERT_4_G Atom Site Occupancy of *O9A_* is Constrained at   0.500 Check
PLAT300_ALERT_4_G Atom Site Occupancy of *O9B_* is Constrained at   0.500 Check
PLAT300_ALERT_4_G Atom Site Occupancy of *O10A_* is Constrained at   0.500 Check
PLAT300_ALERT_4_G Atom Site Occupancy of *O10B_* is Constrained at   0.500 Check
PLAT300_ALERT_4_G Atom Site Occupancy of *O9A_* is Constrained at   0.500 Check
PLAT300_ALERT_4_G Atom Site Occupancy of *O9B_* is Constrained at   0.500 Check
PLAT300_ALERT_4_G Atom Site Occupancy of *O10A_* is Constrained at   0.500 Check
PLAT300_ALERT_4_G Atom Site Occupancy of *O10B_* is Constrained at   0.500 Check
PLAT300_ALERT_4_G Atom Site Occupancy of *O9A_* is Constrained at   0.500 Check
PLAT300_ALERT_4_G Atom Site Occupancy of *O9B_* is Constrained at   0.500 Check
PLAT300_ALERT_4_G Atom Site Occupancy of *O10A_* is Constrained at   0.500 Check
PLAT300_ALERT_4_G Atom Site Occupancy of *O10B_* is Constrained at   0.500 Check
PLAT300_ALERT_4_G Atom Site Occupancy of *O9A_* is Constrained at   0.500 Check
PLAT300_ALERT_4_G Atom Site Occupancy of *O9B_* is Constrained at   0.500 Check
PLAT300_ALERT_4_G Atom Site Occupancy of *O10A_* is Constrained at   0.500 Check
PLAT300_ALERT_4_G Atom Site Occupancy of *O10B_* is Constrained at   0.500 Check
PLAT300_ALERT_4_G Atom Site Occupancy of *O9A_* is Constrained at   0.500 Check
PLAT300_ALERT_4_G Atom Site Occupancy of *O9B_* is Constrained at   0.500 Check
PLAT300_ALERT_4_G Atom Site Occupancy of *O10A_* is Constrained at   0.500 Check
PLAT300_ALERT_4_G Atom Site Occupancy of *O10B_* is Constrained at   0.500 Check
PLAT300_ALERT_4_G Atom Site Occupancy of *O9A_* is Constrained at   0.500 Check
PLAT300_ALERT_4_G Atom Site Occupancy of *O9B_* is Constrained at   0.500 Check
PLAT300_ALERT_4_G Atom Site Occupancy of *O10A_* is Constrained at   0.500 Check
PLAT300_ALERT_4_G Atom Site Occupancy of *O10B_* is Constrained at   0.500 Check
PLAT300_ALERT_4_G Atom Site Occupancy of *O9A_* is Constrained at   0.500 Check
PLAT300_ALERT_4_G Atom Site Occupancy of *O9B_* is Constrained at   0.500 Check
PLAT300_ALERT_4_G Atom Site Occupancy of *O10A_* is Constrained at   0.500 Check
PLAT300_ALERT_4_G Atom Site Occupancy of *O10B_* is Constrained at   0.500 Check
PLAT300_ALERT_4_G Atom Site Occupancy of *O9A_* is Constrained at   0.500 Check
PLAT300_ALERT_4_G Atom Site Occupancy of *O9B_* is Constrained at   0.500 Check
PLAT300_ALERT_4_G Atom Site Occupancy of *O10A_* is Constrained at   0.500 Check

PLAT300_ALERT_4_G Atom Site Occupancy of *O10B_* is Constrained at 0.500 Check
PLAT300_ALERT_4_G Atom Site Occupancy of *O9A_* is Constrained at 0.500 Check
PLAT300_ALERT_4_G Atom Site Occupancy of *O9B_* is Constrained at 0.500 Check
PLAT300_ALERT_4_G Atom Site Occupancy of *O10A_* is Constrained at 0.500 Check
PLAT300_ALERT_4_G Atom Site Occupancy of *O10B_* is Constrained at 0.500 Check
PLAT300_ALERT_4_G Atom Site Occupancy of *O9A_* is Constrained at 0.500 Check
PLAT300_ALERT_4_G Atom Site Occupancy of *O9B_* is Constrained at 0.500 Check
PLAT300_ALERT_4_G Atom Site Occupancy of *O10A_* is Constrained at 0.500 Check
PLAT300_ALERT_4_G Atom Site Occupancy of *O10B_* is Constrained at 0.500 Check
PLAT300_ALERT_4_G Atom Site Occupancy of *O9A_* is Constrained at 0.500 Check
PLAT300_ALERT_4_G Atom Site Occupancy of *O9B_* is Constrained at 0.500 Check
PLAT300_ALERT_4_G Atom Site Occupancy of *O10A_* is Constrained at 0.500 Check
PLAT300_ALERT_4_G Atom Site Occupancy of *O10B_* is Constrained at 0.500 Check
PLAT300_ALERT_4_G Atom Site Occupancy of *O9A_* is Constrained at 0.500 Check
PLAT300_ALERT_4_G Atom Site Occupancy of *O9B_* is Constrained at 0.500 Check
PLAT300_ALERT_4_G Atom Site Occupancy of *O10A_* is Constrained at 0.500 Check
PLAT300_ALERT_4_G Atom Site Occupancy of *O10B_* is Constrained at 0.500 Check
PLAT300_ALERT_4_G Atom Site Occupancy of *O9A_* is Constrained at 0.500 Check
PLAT300_ALERT_4_G Atom Site Occupancy of *O9B_* is Constrained at 0.500 Check
PLAT300_ALERT_4_G Atom Site Occupancy of *O10A_* is Constrained at 0.500 Check
PLAT300_ALERT_4_G Atom Site Occupancy of *O10B_* is Constrained at 0.500 Check
PLAT300_ALERT_4_G Atom Site Occupancy of *O9A_* is Constrained at 0.500 Check
PLAT300_ALERT_4_G Atom Site Occupancy of *O9B_* is Constrained at 0.500 Check
PLAT300_ALERT_4_G Atom Site Occupancy of *O10A_* is Constrained at 0.500 Check
PLAT300_ALERT_4_G Atom Site Occupancy of *O10B_* is Constrained at 0.500 Check
PLAT300_ALERT_4_G Atom Site Occupancy of *O9A_* is Constrained at 0.500 Check
PLAT300_ALERT_4_G Atom Site Occupancy of *O9B_* is Constrained at 0.500 Check
PLAT300_ALERT_4_G Atom Site Occupancy of *O10A_* is Constrained at 0.500 Check
PLAT300_ALERT_4_G Atom Site Occupancy of *O10B_* is Constrained at 0.500 Check
PLAT300_ALERT_4_G Atom Site Occupancy of *O9A_* is Constrained at 0.500 Check
PLAT300_ALERT_4_G Atom Site Occupancy of *O9B_* is Constrained at 0.500 Check
PLAT300_ALERT_4_G Atom Site Occupancy of *O10A_* is Constrained at 0.500 Check
PLAT300_ALERT_4_G Atom Site Occupancy of *O10B_* is Constrained at 0.500 Check
PLAT300_ALERT_4_G Atom Site Occupancy of *O9A_* is Constrained at 0.500 Check
PLAT300_ALERT_4_G Atom Site Occupancy of *O9B_* is Constrained at 0.500 Check
PLAT300_ALERT_4_G Atom Site Occupancy of *O10A_* is Constrained at 0.500 Check
PLAT300_ALERT_4_G Atom Site Occupancy of *O10B_* is Constrained at 0.500 Check
PLAT300_ALERT_4_G Atom Site Occupancy of *O9A_* is Constrained at 0.500 Check
PLAT300_ALERT_4_G Atom Site Occupancy of *O9B_* is Constrained at 0.500 Check
PLAT300_ALERT_4_G Atom Site Occupancy of *O10A_* is Constrained at 0.500 Check
PLAT300_ALERT_4_G Atom Site Occupancy of *O10B_* is Constrained at 0.500 Check
PLAT300_ALERT_4_G Atom Site Occupancy of *O9A_* is Constrained at 0.500 Check
PLAT300_ALERT_4_G Atom Site Occupancy of *O9B_* is Constrained at 0.500 Check
PLAT300_ALERT_4_G Atom Site Occupancy of *O10A_* is Constrained at 0.500 Check
PLAT300_ALERT_4_G Atom Site Occupancy of *O10B_* is Constrained at 0.500 Check
PLAT300_ALERT_4_G Atom Site Occupancy of *O9A_* is Constrained at 0.500 Check
PLAT300_ALERT_4_G Atom Site Occupancy of *O9B_* is Constrained at 0.500 Check
PLAT300_ALERT_4_G Atom Site Occupancy of *O10A_* is Constrained at 0.500 Check
PLAT300_ALERT_4_G Atom Site Occupancy of *O10B_* is Constrained at 0.500 Check
PLAT300_ALERT_4_G Atom Site Occupancy of *O9A_* is Constrained at 0.500 Check
PLAT300_ALERT_4_G Atom Site Occupancy of *O9B_* is Constrained at 0.500 Check
PLAT300_ALERT_4_G Atom Site Occupancy of *O10A_* is Constrained at 0.500 Check
PLAT300_ALERT_4_G Atom Site Occupancy of *O10B_* is Constrained at 0.500 Check
PLAT300_ALERT_4_G Atom Site Occupancy of *O9A_* is Constrained at 0.500 Check
PLAT300_ALERT_4_G Atom Site Occupancy of *O9B_* is Constrained at 0.500 Check
PLAT300_ALERT_4_G Atom Site Occupancy of *O10A_* is Constrained at 0.500 Check
PLAT300_ALERT_4_G Atom Site Occupancy of *O10B_* is Constrained at 0.500 Check
PLAT300_ALERT_4_G Atom Site Occupancy of *O9A_* is Constrained at 0.500 Check
PLAT300_ALERT_4_G Atom Site Occupancy of *O9B_* is Constrained at 0.500 Check
PLAT300_ALERT_4_G Atom Site Occupancy of *O10A_* is Constrained at 0.500 Check
PLAT300_ALERT_4_G Atom Site Occupancy of *O10B_* is Constrained at 0.500 Check
PLAT300_ALERT_4_G Atom Site Occupancy of *O9A_* is Constrained at 0.500 Check

PLAT300_ALERT_4_G Atom Site Occupancy of *O9B_* is Constrained at 0.500 Check
PLAT300_ALERT_4_G Atom Site Occupancy of *O10A_* is Constrained at 0.500 Check
PLAT300_ALERT_4_G Atom Site Occupancy of *O10B_* is Constrained at 0.500 Check
PLAT300_ALERT_4_G Atom Site Occupancy of *O9A_* is Constrained at 0.500 Check
PLAT300_ALERT_4_G Atom Site Occupancy of *O9B_* is Constrained at 0.500 Check
PLAT300_ALERT_4_G Atom Site Occupancy of *O10A_* is Constrained at 0.500 Check
PLAT300_ALERT_4_G Atom Site Occupancy of *O10B_* is Constrained at 0.500 Check
PLAT300_ALERT_4_G Atom Site Occupancy of *O9A_* is Constrained at 0.500 Check
PLAT300_ALERT_4_G Atom Site Occupancy of *O9B_* is Constrained at 0.500 Check
PLAT300_ALERT_4_G Atom Site Occupancy of *O10A_* is Constrained at 0.500 Check
PLAT300_ALERT_4_G Atom Site Occupancy of *O10B_* is Constrained at 0.500 Check
PLAT300_ALERT_4_G Atom Site Occupancy of *O9A_* is Constrained at 0.500 Check
PLAT300_ALERT_4_G Atom Site Occupancy of *O9B_* is Constrained at 0.500 Check
PLAT300_ALERT_4_G Atom Site Occupancy of *O10A_* is Constrained at 0.500 Check
PLAT300_ALERT_4_G Atom Site Occupancy of *O10B_* is Constrained at 0.500 Check
PLAT300_ALERT_4_G Atom Site Occupancy of *O9A_* is Constrained at 0.500 Check
PLAT300_ALERT_4_G Atom Site Occupancy of *O9B_* is Constrained at 0.500 Check
PLAT300_ALERT_4_G Atom Site Occupancy of *O10A_* is Constrained at 0.500 Check
PLAT300_ALERT_4_G Atom Site Occupancy of *O10B_* is Constrained at 0.500 Check
PLAT300_ALERT_4_G Atom Site Occupancy of *O9A_* is Constrained at 0.500 Check
PLAT300_ALERT_4_G Atom Site Occupancy of *O9B_* is Constrained at 0.500 Check
PLAT300_ALERT_4_G Atom Site Occupancy of *O10A_* is Constrained at 0.500 Check
PLAT300_ALERT_4_G Atom Site Occupancy of *O10B_* is Constrained at 0.500 Check
PLAT300_ALERT_4_G Atom Site Occupancy of *O9A_* is Constrained at 0.500 Check
PLAT300_ALERT_4_G Atom Site Occupancy of *O9B_* is Constrained at 0.500 Check
PLAT300_ALERT_4_G Atom Site Occupancy of *O10A_* is Constrained at 0.500 Check
PLAT300_ALERT_4_G Atom Site Occupancy of *O10B_* is Constrained at 0.500 Check
PLAT300_ALERT_4_G Atom Site Occupancy of *O9A_* is Constrained at 0.500 Check
PLAT300_ALERT_4_G Atom Site Occupancy of *O9B_* is Constrained at 0.500 Check
PLAT300_ALERT_4_G Atom Site Occupancy of *O10A_* is Constrained at 0.500 Check
PLAT300_ALERT_4_G Atom Site Occupancy of *O10B_* is Constrained at 0.500 Check
PLAT300_ALERT_4_G Atom Site Occupancy of *O9A_* is Constrained at 0.500 Check
PLAT300_ALERT_4_G Atom Site Occupancy of *O9B_* is Constrained at 0.500 Check
PLAT300_ALERT_4_G Atom Site Occupancy of *O10A_* is Constrained at 0.500 Check
PLAT300_ALERT_4_G Atom Site Occupancy of *O10B_* is Constrained at 0.500 Check
PLAT300_ALERT_4_G Atom Site Occupancy of *O9A_* is Constrained at 0.500 Check
PLAT300_ALERT_4_G Atom Site Occupancy of *O9B_* is Constrained at 0.500 Check
PLAT300_ALERT_4_G Atom Site Occupancy of *O10A_* is Constrained at 0.500 Check
PLAT300_ALERT_4_G Atom Site Occupancy of *O10B_* is Constrained at 0.500 Check
PLAT300_ALERT_4_G Atom Site Occupancy of *O9A_* is Constrained at 0.500 Check
PLAT300_ALERT_4_G Atom Site Occupancy of *O9B_* is Constrained at 0.500 Check
PLAT300_ALERT_4_G Atom Site Occupancy of *O10A_* is Constrained at 0.500 Check
PLAT300_ALERT_4_G Atom Site Occupancy of *O10B_* is Constrained at 0.500 Check
PLAT300_ALERT_4_G Atom Site Occupancy of *O9A_* is Constrained at 0.500 Check
PLAT300_ALERT_4_G Atom Site Occupancy of *O9B_* is Constrained at 0.500 Check
PLAT300_ALERT_4_G Atom Site Occupancy of *O10A_* is Constrained at 0.500 Check
PLAT300_ALERT_4_G Atom Site Occupancy of *O10B_* is Constrained at 0.500 Check
PLAT300_ALERT_4_G Atom Site Occupancy of *O9A_* is Constrained at 0.500 Check
PLAT300_ALERT_4_G Atom Site Occupancy of *O9B_* is Constrained at 0.500 Check
PLAT300_ALERT_4_G Atom Site Occupancy of *O10A_* is Constrained at 0.500 Check
PLAT300_ALERT_4_G Atom Site Occupancy of *O10B_* is Constrained at 0.500 Check
PLAT300_ALERT_4_G Atom Site Occupancy of *O9A_* is Constrained at 0.500 Check
PLAT300_ALERT_4_G Atom Site Occupancy of *O9B_* is Constrained at 0.500 Check
PLAT300_ALERT_4_G Atom Site Occupancy of *O10A_* is Constrained at 0.500 Check
PLAT300_ALERT_4_G Atom Site Occupancy of *O10B_* is Constrained at 0.500 Check
PLAT300_ALERT_4_G Atom Site Occupancy of *O9A_* is Constrained at 0.500 Check
PLAT300_ALERT_4_G Atom Site Occupancy of *O9B_* is Constrained at 0.500 Check
PLAT300_ALERT_4_G Atom Site Occupancy of *O10A_* is Constrained at 0.500 Check
PLAT300_ALERT_4_G Atom Site Occupancy of *O10B_* is Constrained at 0.500 Check
PLAT300_ALERT_4_G Atom Site Occupancy of *O9A_* is Constrained at 0.500 Check
PLAT300_ALERT_4_G Atom Site Occupancy of *O9B_* is Constrained at 0.500 Check
PLAT300_ALERT_4_G Atom Site Occupancy of *O10A_* is Constrained at 0.500 Check

PLAT300_ALERT_4_G Atom Site Occupancy of *O10B_* is Constrained at 0.500 Check
PLAT300_ALERT_4_G Atom Site Occupancy of *O9A_* is Constrained at 0.500 Check
PLAT300_ALERT_4_G Atom Site Occupancy of *O9B_* is Constrained at 0.500 Check
PLAT300_ALERT_4_G Atom Site Occupancy of *O10A_* is Constrained at 0.500 Check
PLAT300_ALERT_4_G Atom Site Occupancy of *O10B_* is Constrained at 0.500 Check
PLAT300_ALERT_4_G Atom Site Occupancy of *O9A_* is Constrained at 0.500 Check
PLAT300_ALERT_4_G Atom Site Occupancy of *O9B_* is Constrained at 0.500 Check
PLAT300_ALERT_4_G Atom Site Occupancy of *O10A_* is Constrained at 0.500 Check
PLAT300_ALERT_4_G Atom Site Occupancy of *O10B_* is Constrained at 0.500 Check
PLAT300_ALERT_4_G Atom Site Occupancy of *O9A_* is Constrained at 0.500 Check
PLAT300_ALERT_4_G Atom Site Occupancy of *O9B_* is Constrained at 0.500 Check
PLAT300_ALERT_4_G Atom Site Occupancy of *O10A_* is Constrained at 0.500 Check
PLAT300_ALERT_4_G Atom Site Occupancy of *O10B_* is Constrained at 0.500 Check
PLAT300_ALERT_4_G Atom Site Occupancy of *O9A_* is Constrained at 0.500 Check
PLAT300_ALERT_4_G Atom Site Occupancy of *O9B_* is Constrained at 0.500 Check
PLAT300_ALERT_4_G Atom Site Occupancy of *O10A_* is Constrained at 0.500 Check
PLAT300_ALERT_4_G Atom Site Occupancy of *O10B_* is Constrained at 0.500 Check
PLAT300_ALERT_4_G Atom Site Occupancy of *O9A_* is Constrained at 0.500 Check
PLAT300_ALERT_4_G Atom Site Occupancy of *O9B_* is Constrained at 0.500 Check
PLAT300_ALERT_4_G Atom Site Occupancy of *O10A_* is Constrained at 0.500 Check
PLAT300_ALERT_4_G Atom Site Occupancy of *O10B_* is Constrained at 0.500 Check
PLAT300_ALERT_4_G Atom Site Occupancy of *O9A_* is Constrained at 0.500 Check
PLAT300_ALERT_4_G Atom Site Occupancy of *O9B_* is Constrained at 0.500 Check
PLAT300_ALERT_4_G Atom Site Occupancy of *O10A_* is Constrained at 0.500 Check
PLAT300_ALERT_4_G Atom Site Occupancy of *O10B_* is Constrained at 0.500 Check
PLAT300_ALERT_4_G Atom Site Occupancy of *O9A_* is Constrained at 0.500 Check
PLAT300_ALERT_4_G Atom Site Occupancy of *O9B_* is Constrained at 0.500 Check
PLAT300_ALERT_4_G Atom Site Occupancy of *O10A_* is Constrained at 0.500 Check
PLAT300_ALERT_4_G Atom Site Occupancy of *O10B_* is Constrained at 0.500 Check
PLAT300_ALERT_4_G Atom Site Occupancy of *O9A_* is Constrained at 0.500 Check
PLAT300_ALERT_4_G Atom Site Occupancy of *O9B_* is Constrained at 0.500 Check
PLAT300_ALERT_4_G Atom Site Occupancy of *O10A_* is Constrained at 0.500 Check
PLAT300_ALERT_4_G Atom Site Occupancy of *O10B_* is Constrained at 0.500 Check
PLAT300_ALERT_4_G Atom Site Occupancy of *O9A_* is Constrained at 0.500 Check
PLAT300_ALERT_4_G Atom Site Occupancy of *O9B_* is Constrained at 0.500 Check
PLAT300_ALERT_4_G Atom Site Occupancy of *O10A_* is Constrained at 0.500 Check
PLAT300_ALERT_4_G Atom Site Occupancy of *O10B_* is Constrained at 0.500 Check
PLAT300_ALERT_4_G Atom Site Occupancy of *O9A_* is Constrained at 0.500 Check
PLAT300_ALERT_4_G Atom Site Occupancy of *O9B_* is Constrained at 0.500 Check
PLAT300_ALERT_4_G Atom Site Occupancy of *O10A_* is Constrained at 0.500 Check
PLAT300_ALERT_4_G Atom Site Occupancy of *O10B_* is Constrained at 0.500 Check
PLAT300_ALERT_4_G Atom Site Occupancy of *O9A_* is Constrained at 0.500 Check
PLAT300_ALERT_4_G Atom Site Occupancy of *O9B_* is Constrained at 0.500 Check
PLAT300_ALERT_4_G Atom Site Occupancy of *O10A_* is Constrained at 0.500 Check
PLAT300_ALERT_4_G Atom Site Occupancy of *O10B_* is Constrained at 0.500 Check
PLAT300_ALERT_4_G Atom Site Occupancy of *O9A_* is Constrained at 0.500 Check
PLAT300_ALERT_4_G Atom Site Occupancy of *O9B_* is Constrained at 0.500 Check
PLAT300_ALERT_4_G Atom Site Occupancy of *O10A_* is Constrained at 0.500 Check
PLAT300_ALERT_4_G Atom Site Occupancy of *O10B_* is Constrained at 0.500 Check
PLAT300_ALERT_4_G Atom Site Occupancy of *O9A_* is Constrained at 0.500 Check
PLAT300_ALERT_4_G Atom Site Occupancy of *O9B_* is Constrained at 0.500 Check
PLAT300_ALERT_4_G Atom Site Occupancy of *O10A_* is Constrained at 0.500 Check
PLAT300_ALERT_4_G Atom Site Occupancy of *O10B_* is Constrained at 0.500 Check
PLAT300_ALERT_4_G Atom Site Occupancy of *O9A_* is Constrained at 0.500 Check
PLAT300_ALERT_4_G Atom Site Occupancy of *O9B_* is Constrained at 0.500 Check
PLAT300_ALERT_4_G Atom Site Occupancy of *O10A_* is Constrained at 0.500 Check
PLAT300_ALERT_4_G Atom Site Occupancy of *O10B_* is Constrained at 0.500 Check
PLAT300_ALERT_4_G Atom Site Occupancy of *O9A_* is Constrained at 0.500 Check
PLAT300_ALERT_4_G Atom Site Occupancy of *O9B_* is Constrained at 0.500 Check
PLAT300_ALERT_4_G Atom Site Occupancy of *O10A_* is Constrained at 0.500 Check
PLAT300_ALERT_4_G Atom Site Occupancy of *O10B_* is Constrained at 0.500 Check
PLAT300_ALERT_4_G Atom Site Occupancy of *O9A_* is Constrained at 0.500 Check

PLAT300_ALERT_4_G Atom Site Occupancy of *O9B_* is Constrained at 0.500 Check
PLAT300_ALERT_4_G Atom Site Occupancy of *O10A_* is Constrained at 0.500 Check
PLAT300_ALERT_4_G Atom Site Occupancy of *O10B_* is Constrained at 0.500 Check
PLAT300_ALERT_4_G Atom Site Occupancy of *O9A_* is Constrained at 0.500 Check
PLAT300_ALERT_4_G Atom Site Occupancy of *O9B_* is Constrained at 0.500 Check
PLAT300_ALERT_4_G Atom Site Occupancy of *O10A_* is Constrained at 0.500 Check
PLAT300_ALERT_4_G Atom Site Occupancy of *O10B_* is Constrained at 0.500 Check
PLAT300_ALERT_4_G Atom Site Occupancy of *O9A_* is Constrained at 0.500 Check
PLAT300_ALERT_4_G Atom Site Occupancy of *O9B_* is Constrained at 0.500 Check
PLAT300_ALERT_4_G Atom Site Occupancy of *O10A_* is Constrained at 0.500 Check
PLAT300_ALERT_4_G Atom Site Occupancy of *O10B_* is Constrained at 0.500 Check
PLAT300_ALERT_4_G Atom Site Occupancy of *O9A_* is Constrained at 0.500 Check
PLAT300_ALERT_4_G Atom Site Occupancy of *O9B_* is Constrained at 0.500 Check
PLAT300_ALERT_4_G Atom Site Occupancy of *O10A_* is Constrained at 0.500 Check
PLAT300_ALERT_4_G Atom Site Occupancy of *O10B_* is Constrained at 0.500 Check
PLAT300_ALERT_4_G Atom Site Occupancy of *O9A_* is Constrained at 0.500 Check
PLAT300_ALERT_4_G Atom Site Occupancy of *O9B_* is Constrained at 0.500 Check
PLAT300_ALERT_4_G Atom Site Occupancy of *O10A_* is Constrained at 0.500 Check
PLAT300_ALERT_4_G Atom Site Occupancy of *O10B_* is Constrained at 0.500 Check
PLAT300_ALERT_4_G Atom Site Occupancy of *O9A_* is Constrained at 0.500 Check
PLAT300_ALERT_4_G Atom Site Occupancy of *O9B_* is Constrained at 0.500 Check
PLAT300_ALERT_4_G Atom Site Occupancy of *O10A_* is Constrained at 0.500 Check
PLAT300_ALERT_4_G Atom Site Occupancy of *O10B_* is Constrained at 0.500 Check
PLAT300_ALERT_4_G Atom Site Occupancy of *O9A_* is Constrained at 0.500 Check
PLAT300_ALERT_4_G Atom Site Occupancy of *O9B_* is Constrained at 0.500 Check
PLAT300_ALERT_4_G Atom Site Occupancy of *O10A_* is Constrained at 0.500 Check
PLAT300_ALERT_4_G Atom Site Occupancy of *O10B_* is Constrained at 0.500 Check
PLAT300_ALERT_4_G Atom Site Occupancy of *O9A_* is Constrained at 0.500 Check
PLAT300_ALERT_4_G Atom Site Occupancy of *O9B_* is Constrained at 0.500 Check
PLAT300_ALERT_4_G Atom Site Occupancy of *O10A_* is Constrained at 0.500 Check
PLAT300_ALERT_4_G Atom Site Occupancy of *O10B_* is Constrained at 0.500 Check
PLAT300_ALERT_4_G Atom Site Occupancy of *O9A_* is Constrained at 0.500 Check
PLAT300_ALERT_4_G Atom Site Occupancy of *O9B_* is Constrained at 0.500 Check
PLAT300_ALERT_4_G Atom Site Occupancy of *O10A_* is Constrained at 0.500 Check
PLAT300_ALERT_4_G Atom Site Occupancy of *O10B_* is Constrained at 0.500 Check
PLAT300_ALERT_4_G Atom Site Occupancy of *O9A_* is Constrained at 0.500 Check
PLAT300_ALERT_4_G Atom Site Occupancy of *O9B_* is Constrained at 0.500 Check
PLAT300_ALERT_4_G Atom Site Occupancy of *O10A_* is Constrained at 0.500 Check
PLAT300_ALERT_4_G Atom Site Occupancy of *O10B_* is Constrained at 0.500 Check
PLAT300_ALERT_4_G Atom Site Occupancy of *O9A_* is Constrained at 0.500 Check
PLAT300_ALERT_4_G Atom Site Occupancy of *O9B_* is Constrained at 0.500 Check
PLAT300_ALERT_4_G Atom Site Occupancy of *O10A_* is Constrained at 0.500 Check
PLAT300_ALERT_4_G Atom Site Occupancy of *O10B_* is Constrained at 0.500 Check
PLAT300_ALERT_4_G Atom Site Occupancy of *O9A_* is Constrained at 0.500 Check
PLAT300_ALERT_4_G Atom Site Occupancy of *O9B_* is Constrained at 0.500 Check
PLAT300_ALERT_4_G Atom Site Occupancy of *O10A_* is Constrained at 0.500 Check
PLAT300_ALERT_4_G Atom Site Occupancy of *O10B_* is Constrained at 0.500 Check
PLAT300_ALERT_4_G Atom Site Occupancy of *O9A_* is Constrained at 0.500 Check
PLAT300_ALERT_4_G Atom Site Occupancy of *O9B_* is Constrained at 0.500 Check
PLAT300_ALERT_4_G Atom Site Occupancy of *O10A_* is Constrained at 0.500 Check
PLAT300_ALERT_4_G Atom Site Occupancy of *O10B_* is Constrained at 0.500 Check
PLAT300_ALERT_4_G Atom Site Occupancy of *O9A_* is Constrained at 0.500 Check
PLAT300_ALERT_4_G Atom Site Occupancy of *O9B_* is Constrained at 0.500 Check
PLAT300_ALERT_4_G Atom Site Occupancy of *O10A_* is Constrained at 0.500 Check
PLAT300_ALERT_4_G Atom Site Occupancy of *O10B_* is Constrained at 0.500 Check
PLAT300_ALERT_4_G Atom Site Occupancy of *O9A_* is Constrained at 0.500 Check
PLAT300_ALERT_4_G Atom Site Occupancy of *O9B_* is Constrained at 0.500 Check
PLAT300_ALERT_4_G Atom Site Occupancy of *O10A_* is Constrained at 0.500 Check
PLAT300_ALERT_4_G Atom Site Occupancy of *O10B_* is Constrained at 0.500 Check
PLAT300_ALERT_4_G Atom Site Occupancy of *O9A_* is Constrained at 0.500 Check
PLAT300_ALERT_4_G Atom Site Occupancy of *O9B_* is Constrained at 0.500 Check
PLAT300_ALERT_4_G Atom Site Occupancy of *O10A_* is Constrained at 0.500 Check

```
PLAT300_ALERT_4_G Atom Site Occupancy of *O10B_* is Constrained at      0.500 Check
PLAT300_ALERT_4_G Atom Site Occupancy of *O9A_*  is Constrained at      0.500 Check
PLAT300_ALERT_4_G Atom Site Occupancy of *O9B_*  is Constrained at      0.500 Check
PLAT300_ALERT_4_G Atom Site Occupancy of *O10A_* is Constrained at      0.500 Check
PLAT300_ALERT_4_G Atom Site Occupancy of *O10B_* is Constrained at      0.500 Check
PLAT300_ALERT_4_G Atom Site Occupancy of *O9A_*  is Constrained at      0.500 Check
PLAT300_ALERT_4_G Atom Site Occupancy of *O9B_*  is Constrained at      0.500 Check
PLAT300_ALERT_4_G Atom Site Occupancy of *O10A_* is Constrained at      0.500 Check
PLAT300_ALERT_4_G Atom Site Occupancy of *O10B_* is Constrained at      0.500 Check
PLAT300_ALERT_4_G Atom Site Occupancy of *O9A_*  is Constrained at      0.500 Check
PLAT300_ALERT_4_G Atom Site Occupancy of *O9B_*  is Constrained at      0.500 Check
PLAT300_ALERT_4_G Atom Site Occupancy of *O10A_* is Constrained at      0.500 Check
PLAT300_ALERT_4_G Atom Site Occupancy of *O10B_* is Constrained at      0.500 Check
PLAT300_ALERT_4_G Atom Site Occupancy of *O9A_*  is Constrained at      0.500 Check
PLAT300_ALERT_4_G Atom Site Occupancy of *O9B_*  is Constrained at      0.500 Check
PLAT300_ALERT_4_G Atom Site Occupancy of *O10A_* is Constrained at      0.500 Check
PLAT300_ALERT_4_G Atom Site Occupancy of *O10B_* is Constrained at      0.500 Check
PLAT300_ALERT_4_G Atom Site Occupancy of *O9A_*  is Constrained at      0.500 Check
PLAT300_ALERT_4_G Atom Site Occupancy of *O9B_*  is Constrained at      0.500 Check
PLAT300_ALERT_4_G Atom Site Occupancy of *O10A_* is Constrained at      0.500 Check
PLAT300_ALERT_4_G Atom Site Occupancy of *O10B_* is Constrained at      0.500 Check
PLAT300_ALERT_4_G Atom Site Occupancy of *O9A_*  is Constrained at      0.500 Check
PLAT300_ALERT_4_G Atom Site Occupancy of *O9B_*  is Constrained at      0.500 Check
PLAT300_ALERT_4_G Atom Site Occupancy of *O10A_* is Constrained at      0.500 Check
PLAT300_ALERT_4_G Atom Site Occupancy of *O10B_* is Constrained at      0.500 Check
PLAT300_ALERT_4_G Atom Site Occupancy of *O9A_*  is Constrained at      0.500 Check
PLAT300_ALERT_4_G Atom Site Occupancy of *O9B_*  is Constrained at      0.500 Check
PLAT300_ALERT_4_G Atom Site Occupancy of *O10A_* is Constrained at      0.500 Check
PLAT300_ALERT_4_G Atom Site Occupancy of *O10B_* is Constrained at      0.500 Check
PLAT300_ALERT_4_G Atom Site Occupancy of *O9A_*  is Constrained at      0.500 Check
PLAT300_ALERT_4_G Atom Site Occupancy of *O9B_*  is Constrained at      0.500 Check
PLAT300_ALERT_4_G Atom Site Occupancy of *O10A_* is Constrained at      0.500 Check
PLAT300_ALERT_4_G Atom Site Occupancy of *O10B_* is Constrained at      0.500 Check
PLAT300_ALERT_4_G Atom Site Occupancy of *O9A_*  is Constrained at      0.500 Check
PLAT300_ALERT_4_G Atom Site Occupancy of *O9B_*  is Constrained at      0.500 Check
PLAT300_ALERT_4_G Atom Site Occupancy of *O10A_* is Constrained at      0.500 Check
PLAT300_ALERT_4_G Atom Site Occupancy of *O10B_* is Constrained at      0.500 Check
PLAT301_ALERT_3_G Main Residue  Disorder ............ Percentage =         12 Note
PLAT606_ALERT_4_G VERY LARGE Solvent Accessible VOID(S) in Structure        ! Info
PLAT764_ALERT_4_G Overcomplete CIF Bond List Detected (Rep/Expd) .       1.19 Ratio
PLAT793_ALERT_4_G The Model has Chirality at P1      (Centro SPGR)          R Verify
PLAT794_ALERT_5_G Tentative Bond Valency for Mo1     (VI)    .....       5.68 Note
PLAT869_ALERT_4_G ALERTS Related to the use of SQUEEZE Suppressed           ! Info
PLAT899_ALERT_4_G SHELXL97   is Deprecated and Succeeded by SHELXL       2014 Note
PLAT951_ALERT_5_G Calculated (ThMax) and CIF-Reported Kmax Differ          10 Units
PLAT952_ALERT_5_G Calculated (ThMax) and CIF-Reported Lmax Differ           7 Units
```

---

0 **ALERT level A** = Most likely a serious problem - resolve or explain
5 **ALERT level B** = A potentially serious problem, consider carefully
11 **ALERT level C** = Check. Ensure it is not caused by an omission or oversight
387 **ALERT level G** = General information/check it is not something unexpected

3 ALERT type 1 CIF construction/syntax error, inconsistent or missing data
13 ALERT type 2 Indicator that the structure model may be wrong or deficient
4 ALERT type 3 Indicator that the structure quality may be low
378 ALERT type 4 Improvement, methodology, query or suggestion
5 ALERT type 5 Informative message, check

It is advisable to attempt to resolve as many as possible of the alerts in all categories. Often the minor alerts point to easily fixed oversights, errors and omissions in your CIF or refinement strategy, so attention to these fine details can be worthwhile. In order to resolve some of the more serious problems it may be necessary to carry out additional measurements or structure refinements. However, the purpose of your study may justify the reported deviations and the more serious of these should normally be commented upon in the discussion or experimental section of a paper or in the "special_details" fields of the CIF. checkCIF was carefully designed to identify outliers and unusual parameters, but every test has its limitations and alerts that are not important in a particular case may appear. Conversely, the absence of alerts does not guarantee there are no aspects of the results needing attention. It is up to the individual to critically assess their own results and, if necessary, seek expert advice.

Publication of your CIF in IUCr journals

A basic structural check has been run on your CIF. These basic checks will be run on all CIFs submitted for publication in IUCr journals (*Acta Crystallographica*, *Journal of Applied Crystallography*, *Journal of Synchrotron Radiation*); however, if you intend to submit to *Acta Crystallographica Section C* or *E*, you should make sure that full publication checks are run on the final version of your CIF prior to submission.

Publication of your CIF in other journals

Please refer to the *Notes for Authors* of the relevant journal for any special instructions relating to CIF submission.

PLATON version of 29/01/2015; check.def file version of 29/01/2015

Datablock HLJU-1 - ellipsoid plot

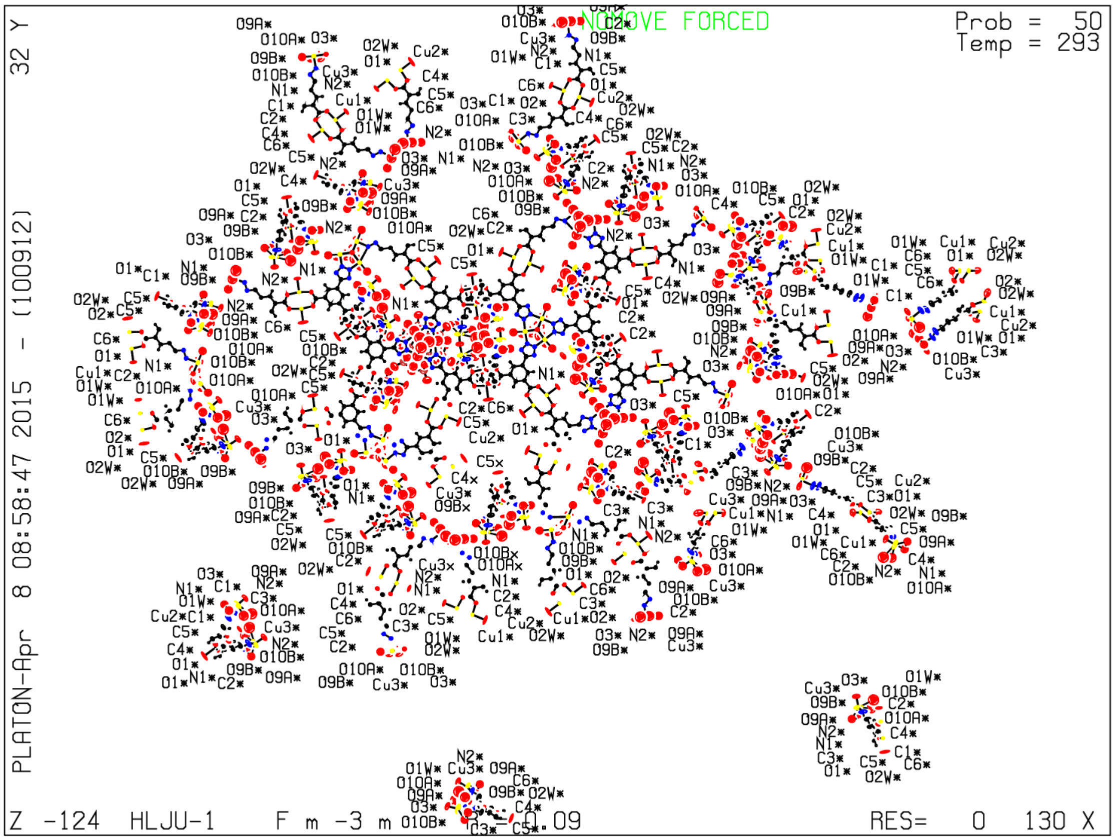

checkCIF/PLATON report

You have not supplied any structure factors. As a result the full set of tests cannot be run.

THIS REPORT IS FOR GUIDANCE ONLY. IF USED AS PART OF A REVIEW PROCEDURE FOR PUBLICATION, IT SHOULD NOT REPLACE THE EXPERTISE OF AN EXPERIENCED CRYSTALLOGRAPHIC REFEREE.

No syntax errors found. CIF dictionary Interpreting this report

Datablock: HLJU-2

```
Bond precision:  C-C = 0.0085 A          Wavelength=0.71069

Cell:              a=44.614(5)      b=44.614(5)      c=44.614(5)
                   alpha=90         beta=90          gamma=90
Temperature:       293 K
```

	Calculated	Reported
Volume	88800(30)	88800(30)
Space group	F m -3 m	F m -3 m
Hall group	-F 4 2 3	-F 4 2 3
Moiety formula	4(C27 H9 Cu6 N12 O22), Mo12 O40 Si	4(C27 H9 Cu6 N12 O22), Mo12 O40 Si
Sum formula	C108 H36 Cu24 Mo12 N48 O128 Si	C108 H36 Cu24 Mo12 N48 O128 Si
Mr	6758.42	6758.42
Dx,g cm-3	1.011	1.011
Z	8	8
Mu (mm-1)	1.504	1.504
F000	26064.0	26064.0
F000’	25972.20	
h,k,lmax	53,53,53	36,53,48
Nref	3810	3810
Tmin,Tmax	0.748,0.740	0.740,0.748
Tmin’	0.733	

```
Correction method= # Reported T Limits: Tmin=0.740 Tmax=0.748
AbsCorr = MULTI-SCAN

Data completeness= 1.000          Theta(max)= 24.980

R(reflections)= 0.0710( 2531)     wR2(reflections)= 0.2188( 3810)

S = 0.990                   Npar= 147
```

The following ALERTS were generated. Each ALERT has the format
test-name_ALERT_alert-type_alert-level.
Click on the hyperlinks for more details of the test.

Alert level B

```
PLAT220_ALERT_2_B Large Non-Solvent  O     Ueq(max)/Ueq(min) Range        8.3 Ratio
PLAT242_ALERT_2_B Low       Ueq as Compared to Neighbors for .....        Cu1 Check
PLAT242_ALERT_2_B Low       Ueq as Compared to Neighbors for .....        Cu3 Check
PLAT250_ALERT_2_B Large U3/U1 Ratio for Average U(i,j) Tensor ....        4.9 Note
```

Alert level C

```
PLAT147_ALERT_1_C su on Symmetry Constrained Cell Angle(s) .......     Please Check
PLAT213_ALERT_2_C Atom O2              has ADP max/min Ratio .....        3.6 prolat
PLAT213_ALERT_2_C Atom O6              has ADP max/min Ratio .....        3.8 prolat
PLAT213_ALERT_2_C Atom O7              has ADP max/min Ratio .....        3.9 prolat
PLAT220_ALERT_2_C Large Non-Solvent Cu     Ueq(max)/Ueq(min) Range        3.2 Ratio
PLAT230_ALERT_2_C Hirshfeld Test Diff for    Si1    --  O10      ..       6.4 su
PLAT232_ALERT_2_C Hirshfeld Test Diff (M-X)  Cu3    --  O7       ..       9.5 su
PLAT232_ALERT_2_C Hirshfeld Test Diff (M-X)  Mo1    --  O9       ..       6.2 su
PLAT232_ALERT_2_C Hirshfeld Test Diff (M-X)  Mo1    --  O11      ..       6.8 su
PLAT234_ALERT_4_C Large Hirshfeld Difference Cu3    --  O6       ..      0.22 Ang.
PLAT242_ALERT_2_C Low       Ueq as Compared to Neighbors for .....        Cu2 Check
PLAT342_ALERT_3_C Low Bond Precision on  C-C Bonds ...............     0.0085 Ang.
PLAT731_ALERT_1_C Bond    Calc    1.908(7), Rep    1.908(2) ......          4 su-Rat
              MO1  -O11     1.555   1.555                #         18
PLAT731_ALERT_1_C Bond    Calc    1.908(7), Rep    1.908(2) ......          4 su-Rat
              MO1  -O11     1.555  12.555                #         19
PLAT731_ALERT_1_C Bond    Calc    1.908(7), Rep    1.908(2) ......          4 su-Rat
              O11  -MO1     1.555  11.555                #         33
PLAT732_ALERT_1_C Angle   Calc     91.6(5), Rep   91.60(15) ......       3.33 su-Rat
              N1   -CU3  -O6      1.555   1.555   1.555      #        33
PLAT732_ALERT_1_C Angle   Calc     91.6(5), Rep     91.6(2) ......       2.50 su-Rat
              N1   -CU3  -O7      1.555   1.555   1.555      #        38
```

Alert level G

```
PLAT004_ALERT_5_G Polymeric Structure Found with Maximum Dimension          3 Info
PLAT005_ALERT_5_G No _iucr_refine_instructions_details  in the CIF     Please Do !
PLAT072_ALERT_2_G SHELXL First  Parameter in WGHT Unusually Large.       0.13 Report
PLAT199_ALERT_1_G Reported _cell_measurement_temperature ..... (K)        293 Check
PLAT200_ALERT_1_G Reported   _diffrn_ambient_temperature ..... (K)        293 Check
PLAT606_ALERT_4_G VERY LARGE Solvent Accessible VOID(S) in Structure        ! Info
PLAT764_ALERT_4_G Overcomplete CIF Bond List Detected (Rep/Expd) .       1.25 Ratio
PLAT793_ALERT_4_G The Model has Chirality at Si1     (Centro SPGR)          S Verify
PLAT794_ALERT_5_G Tentative Bond Valency for Mo1     (VI)    .....       6.08 Note
PLAT869_ALERT_4_G ALERTS Related to the use of SQUEEZE Suppressed           ! Info
PLAT899_ALERT_4_G SHELXL97   is Deprecated and Succeeded by SHELXL       2014 Note
PLAT950_ALERT_5_G Calculated (ThMax) and CIF-Reported Hmax Differ          17 Units
PLAT952_ALERT_5_G Calculated (ThMax) and CIF-Reported Lmax Differ           5 Units
```

---

0 **ALERT level A** = Most likely a serious problem - resolve or explain
4 **ALERT level B** = A potentially serious problem, consider carefully
17 **ALERT level C** = Check. Ensure it is not caused by an omission or oversight
13 **ALERT level G** = General information/check it is not something unexpected

8 ALERT type 1 CIF construction/syntax error, inconsistent or missing data

```
14 ALERT type 2 Indicator that the structure model may be wrong or deficient
 1 ALERT type 3 Indicator that the structure quality may be low
 6 ALERT type 4 Improvement, methodology, query or suggestion
 5 ALERT type 5 Informative message, check
```

It is advisable to attempt to resolve as many as possible of the alerts in all categories. Often the minor alerts point to easily fixed oversights, errors and omissions in your CIF or refinement strategy, so attention to these fine details can be worthwhile. In order to resolve some of the more serious problems it may be necessary to carry out additional measurements or structure refinements. However, the purpose of your study may justify the reported deviations and the more serious of these should normally be commented upon in the discussion or experimental section of a paper or in the "special_details" fields of the CIF. checkCIF was carefully designed to identify outliers and unusual parameters, but every test has its limitations and alerts that are not important in a particular case may appear. Conversely, the absence of alerts does not guarantee there are no aspects of the results needing attention. It is up to the individual to critically assess their own results and, if necessary, seek expert advice.

Publication of your CIF in IUCr journals

A basic structural check has been run on your CIF. These basic checks will be run on all CIFs submitted for publication in IUCr journals (*Acta Crystallographica*, *Journal of Applied Crystallography*, *Journal of Synchrotron Radiation*); however, if you intend to submit to *Acta Crystallographica Section C* or *E*, you should make sure that full publication checks are run on the final version of your CIF prior to submission.

Publication of your CIF in other journals

Please refer to the *Notes for Authors* of the relevant journal for any special instructions relating to CIF submission.

PLATON version of 29/01/2015; check.def file version of 29/01/2015

Datablock HLJU-2 - ellipsoid plot

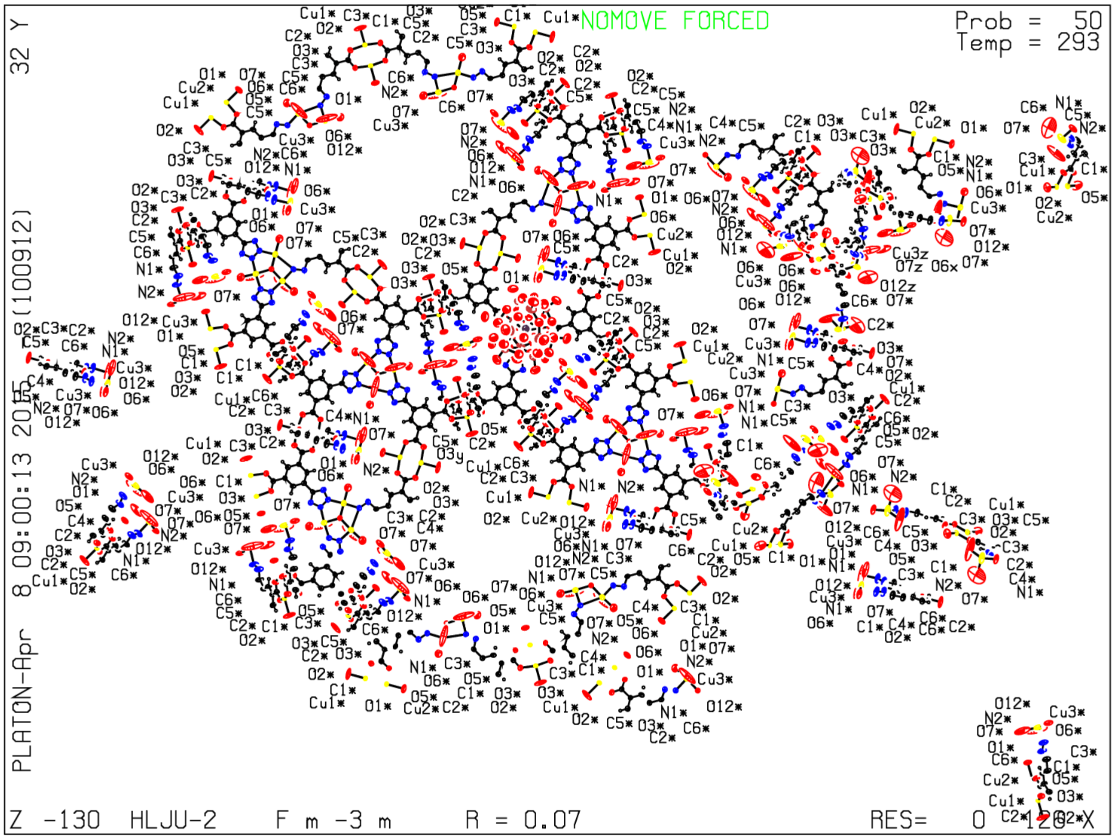

checkCIF/PLATON report

You have not supplied any structure factors. As a result the full set of tests cannot be run.

THIS REPORT IS FOR GUIDANCE ONLY. IF USED AS PART OF A REVIEW PROCEDURE FOR PUBLICATION, IT SHOULD NOT REPLACE THE EXPERTISE OF AN EXPERIENCED CRYSTALLOGRAPHIC REFEREE.

No syntax errors found.   CIF dictionary   Interpreting this report

Datablock: hlju-3

```
Bond precision:  C-C = 0.0098 A          Wavelength=0.71073

Cell:            a=44.4742(7)     b=44.4742(7)     c=44.4742(7)
                 alpha=90         beta=90          gamma=90
Temperature:     293 K
```

	Calculated	Reported
Volume	87968(4)	87968(4)
Space group	F m -3 m	F m -3 m
Hall group	-F 4 2 3	-F 4 2 3
Moiety formula	2(C54 H18 Cu12 N24 O41), O40 P W12	2(C54 H18 Cu12 N24 O41), O40 P W12
Sum formula	C108 H36 Cu24 N48 O122 P W12	C108 H36 Cu24 N48 O122 P W12
Mr	7720.10	7720.10
Dx,g cm-3	1.166	1.166
Z	8	8
Mu (mm-1)	4.312	4.312
F000	28760.0	28760.0
F000’	28760.09	
h,k,lmax	52,52,52	30,52,37
Nref	3777	3777
Tmin,Tmax	0.439,0.422	0.422,0.439
Tmin’	0.406	

```
Correction method= # Reported T Limits: Tmin=0.422 Tmax=0.439
AbsCorr = MULTI-SCAN

Data completeness= 1.000          Theta(max)= 24.990

R(reflections)= 0.0994( 1872)     wR2(reflections)= 0.2547( 3777)

S = 0.853                  Npar= 79
```

The following ALERTS were generated. Each ALERT has the format
test-name_ALERT_alert-type_alert-level.
Click on the hyperlinks for more details of the test.

Alert level A

```
PLAT075_ALERT_1_A Occupancy      2.000 greater than 1.0 for ......         O2
PLAT201_ALERT_2_A Isotropic non-H Atoms in Main Residue(s) .......         18 Report
PLAT241_ALERT_2_A High   'MainMol' Ueq as Compared to Neighbors of         W1 Check
PLAT242_ALERT_2_A Low    'MainMol' Ueq as Compared to Neighbors of        Cu2 Check
```

Alert level B

```
PLAT213_ALERT_2_B Atom Cu3             has ADP max/min Ratio .....        4.6 prolat
PLAT220_ALERT_2_B Large Non-Solvent Cu     Ueq(max)/Ueq(min) Range        6.6 Ratio
PLAT220_ALERT_2_B Large Non-Solvent  O     Ueq(max)/Ueq(min) Range        7.2 Ratio
PLAT780_ALERT_1_B Coordinates do not Form a Properly Connected Set     Please Do !
```

Alert level C

```
RFACR01_ALERT_3_C  The value of the weighted R factor is > 0.25
            Weighted R factor given   0.255
RINTA01_ALERT_3_C  The value of Rint is greater than 0.12
            Rint given   0.124
PLAT020_ALERT_3_C The value of Rint is greater than 0.12 .........      0.124 Report
PLAT029_ALERT_3_C _diffrn_measured_fraction_theta_full Low .......      0.975 Note
PLAT241_ALERT_2_C High   'MainMol' Ueq as Compared to Neighbors of        Cu3 Check
PLAT242_ALERT_2_C Low    'MainMol' Ueq as Compared to Neighbors of        Cu1 Check
PLAT242_ALERT_2_C Low    'MainMol' Ueq as Compared to Neighbors of         N4 Check
PLAT342_ALERT_3_C Low Bond Precision on  C-C Bonds ...............    0.00983 Ang.
PLAT369_ALERT_2_C Long    C(sp2)-C(sp2) Bond  C1      -   C2      ..       1.55 Ang.
PLAT369_ALERT_2_C Long    C(sp2)-C(sp2) Bond  C7      -   C10     ..       1.53 Ang.
PLAT732_ALERT_1_C Angle   Calc     91.7(5), Rep     91.7(2) ......       2.50 s.u.-R
              N4   -CU3  -O30     1.555   1.555   1.555      #         35
```

Alert level G

```
PLAT002_ALERT_2_G Number of Distance or Angle Restraints on AtSite          6 Note
PLAT004_ALERT_5_G Polymeric Structure Found with Maximum Dimension          3 Info
PLAT005_ALERT_5_G No Embedded Refinement Details found in the CIF      Please Do !
PLAT072_ALERT_2_G SHELXL First  Parameter in WGHT  Unusually Large       0.16 Report
PLAT199_ALERT_1_G Reported _cell_measurement_temperature ..... (K)        293 Check
PLAT200_ALERT_1_G Reported   _diffrn_ambient_temperature ..... (K)        293 Check
PLAT300_ALERT_4_G Atom Site Occupancy of <P1     is Constrained at       0.25 Check
PLAT301_ALERT_3_G Main Residue  Disorder ............ Percentage =          0 Note
PLAT432_ALERT_2_G Short Inter X...Y Contact  O78     ..  C1      ..       2.75 Ang.
PLAT606_ALERT_4_G VERY LARGE Solvent Accessible VOID(S) in Structure        ! Info
PLAT779_ALERT_4_G Suspect or Irrelevant (Bond) Angle in CIF .... #        113 Check
              P1   -P1   -O96    59.555   1.555   8.555             40.10 Deg.
PLAT779_ALERT_4_G Suspect or Irrelevant (Bond) Angle in CIF .... #        115 Check
              P1   -P1   -O96    63.555   1.555   8.555             40.10 Deg.
PLAT779_ALERT_4_G Suspect or Irrelevant (Bond) Angle in CIF .... #        117 Check
              P1   -P1   -O96    64.555   1.555 154.555             40.10 Deg.
PLAT779_ALERT_4_G Suspect or Irrelevant (Bond) Angle in CIF .... #        118 Check
              P1   -P1   -O96    59.555   1.555 108.555             40.10 Deg.
PLAT779_ALERT_4_G Suspect or Irrelevant (Bond) Angle in CIF .... #        119 Check
              P1   -P1   -O96    63.555   1.555 154.555             40.10 Deg.
PLAT779_ALERT_4_G Suspect or Irrelevant (Bond) Angle in CIF .... #        120 Check
              P1   -P1   -O96    64.555   1.555 108.555             40.10 Deg.
PLAT860_ALERT_3_G Number of Least-Squares Restraints .............          7 Note
PLAT869_ALERT_4_G ALERTS Related to the use of SQUEEZE Suppressed           ! Info
```

```
PLAT982_ALERT_1_G The Cu-f'=     0.341 Deviates from the  IT-value      0.320 Check
PLAT982_ALERT_1_G The  P-f'=     0.104 Deviates from the  IT-value      0.102 Check
PLAT982_ALERT_1_G The  W-f'=    -0.668 Deviates from the  IT-value     -0.849 Check
PLAT983_ALERT_1_G The Cu-f"=     1.289 Deviates from the  IT-Value      1.265 Check
PLAT983_ALERT_1_G The  W-f"=     6.765 Deviates from the  IT-Value      6.872 Check
```

```
   4 ALERT level A = Most likely a serious problem - resolve or explain
   4 ALERT level B = A potentially serious problem, consider carefully
  11 ALERT level C = Check. Ensure it is not caused by an omission or oversight
  23 ALERT level G = General information/check it is not something unexpected

  10 ALERT type 1 CIF construction/syntax error, inconsistent or missing data
  14 ALERT type 2 Indicator that the structure model may be wrong or deficient
   7 ALERT type 3 Indicator that the structure quality may be low
   9 ALERT type 4 Improvement, methodology, query or suggestion
   2 ALERT type 5 Informative message, check
```

It is advisable to attempt to resolve as many as possible of the alerts in all categories. Often the minor alerts point to easily fixed oversights, errors and omissions in your CIF or refinement strategy, so attention to these fine details can be worthwhile. In order to resolve some of the more serious problems it may be necessary to carry out additional measurements or structure refinements. However, the purpose of your study may justify the reported deviations and the more serious of these should normally be commented upon in the discussion or experimental section of a paper or in the "special_details" fields of the CIF. checkCIF was carefully designed to identify outliers and unusual parameters, but every test has its limitations and alerts that are not important in a particular case may appear. Conversely, the absence of alerts does not guarantee there are no aspects of the results needing attention. It is up to the individual to critically assess their own results and, if necessary, seek expert advice.

Publication of your CIF in IUCr journals

A basic structural check has been run on your CIF. These basic checks will be run on all CIFs submitted for publication in IUCr journals (*Acta Crystallographica*, *Journal of Applied Crystallography*, *Journal of Synchrotron Radiation*); however, if you intend to submit to *Acta Crystallographica Section C* or *E*, you should make sure that full publication checks are run on the final version of your CIF prior to submission.

Publication of your CIF in other journals

Please refer to the *Notes for Authors* of the relevant journal for any special instructions relating to CIF submission.

PLATON version of 19/11/2015; check.def file version of 17/11/2015

Datablock hlju-3 - ellipsoid plot

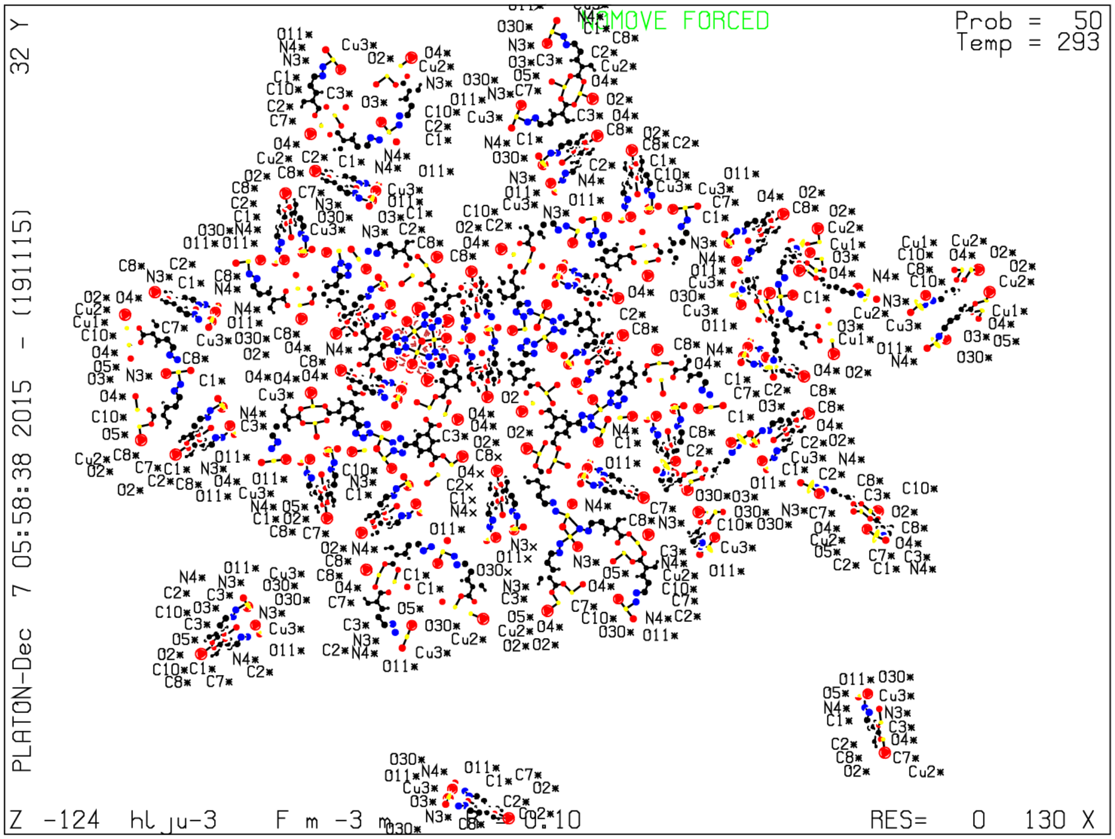

```
data_HLJU-1

_audit_creation_method            SHELXL-97
_chemical_name_systematic
;
 ?
;
_chemical_name_common             ?
_chemical_melting_point           ?
_chemical_formula_moiety          ' 4(C27 H9 Cu6 N12 O22), Mo12 O40 P '
_chemical_formula_sum
 'C108 H36 Cu24 Mo12 N48 O128 P '
_chemical_formula_weight          6761.30

loop_
 _atom_type_symbol
 _atom_type_description
 _atom_type_scat_dispersion_real
 _atom_type_scat_dispersion_imag
 _atom_type_scat_source
 'C'  'C'   0.0033   0.0016
 'International Tables Vol C Tables 4.2.6.8 and 6.1.1.4'
 'H'  'H'   0.0000   0.0000
 'International Tables Vol C Tables 4.2.6.8 and 6.1.1.4'
 'N'  'N'   0.0061   0.0033
 'International Tables Vol C Tables 4.2.6.8 and 6.1.1.4'
 'O'  'O'   0.0106   0.0060
 'International Tables Vol C Tables 4.2.6.8 and 6.1.1.4'
 'P'  'P'   0.1023   0.0942
 'International Tables Vol C Tables 4.2.6.8 and 6.1.1.4'
 'Cl'  'Cl'   0.1484   0.1585
 'International Tables Vol C Tables 4.2.6.8 and 6.1.1.4'
 'Cu'  'Cu'   0.3201   1.2651
 'International Tables Vol C Tables 4.2.6.8 and 6.1.1.4'
 'Mo'  'Mo'  -1.6832   0.6857
 'International Tables Vol C Tables 4.2.6.8 and 6.1.1.4'

_symmetry_cell_setting            cubic
_symmetry_space_group_name_H-M    'F m -3 m '
_symmetry_space_group_name_Hall   '-F 4 2 3   '

loop_
 _symmetry_equiv_pos_as_xyz
```

'x, y, z'
'-x, -y, z'
'-x, y, -z'
'x, -y, -z'
'z, x, y'
'z, -x, -y'
'-z, -x, y'
'-z, x, -y'
'y, z, x'
'-y, z, -x'
'y, -z, -x'
'-y, -z, x'
'y, x, -z'
'-y, -x, -z'
'y, -x, z'
'-y, x, z'
'x, z, -y'
'-x, z, y'
'-x, -z, -y'
'x, -z, y'
'z, y, -x'
'z, -y, x'
'-z, y, x'
'-z, -y, -x'
'x, y+1/2, z+1/2'
'-x, -y+1/2, z+1/2'
'-x, y+1/2, -z+1/2'
'x, -y+1/2, -z+1/2'
'z, x+1/2, y+1/2'
'z, -x+1/2, -y+1/2'
'-z, -x+1/2, y+1/2'
'-z, x+1/2, -y+1/2'
'y, z+1/2, x+1/2'
'-y, z+1/2, -x+1/2'
'y, -z+1/2, -x+1/2'
'-y, -z+1/2, x+1/2'
'y, x+1/2, -z+1/2'
'-y, -x+1/2, -z+1/2'
'y, -x+1/2, z+1/2'
'-y, x+1/2, z+1/2'
'x, z+1/2, -y+1/2'
'-x, z+1/2, y+1/2'
'-x, -z+1/2, -y+1/2'
'x, -z+1/2, y+1/2'

'z, y+1/2, -x+1/2'
'z, -y+1/2, x+1/2'
'-z, y+1/2, x+1/2'
'-z, -y+1/2, -x+1/2'
'x+1/2, y, z+1/2'
'-x+1/2, -y, z+1/2'
'-x+1/2, y, -z+1/2'
'x+1/2, -y, -z+1/2'
'z+1/2, x, y+1/2'
'z+1/2, -x, -y+1/2'
'-z+1/2, -x, y+1/2'
'-z+1/2, x, -y+1/2'
'y+1/2, z, x+1/2'
'-y+1/2, z, -x+1/2'
'y+1/2, -z, -x+1/2'
'-y+1/2, -z, x+1/2'
'y+1/2, x, -z+1/2'
'-y+1/2, -x, -z+1/2'
'y+1/2, -x, z+1/2'
'-y+1/2, x, z+1/2'
'x+1/2, z, -y+1/2'
'-x+1/2, z, y+1/2'
'-x+1/2, -z, -y+1/2'
'x+1/2, -z, y+1/2'
'z+1/2, y, -x+1/2'
'z+1/2, -y, x+1/2'
'-z+1/2, y, x+1/2'
'-z+1/2, -y, -x+1/2'
'x+1/2, y+1/2, z'
'-x+1/2, -y+1/2, z'
'-x+1/2, y+1/2, -z'
'x+1/2, -y+1/2, -z'
'z+1/2, x+1/2, y'
'z+1/2, -x+1/2, -y'
'-z+1/2, -x+1/2, y'
'-z+1/2, x+1/2, -y'
'y+1/2, z+1/2, x'
'-y+1/2, z+1/2, -x'
'y+1/2, -z+1/2, -x'
'-y+1/2, -z+1/2, x'
'y+1/2, x+1/2, -z'
'-y+1/2, -x+1/2, -z'
'y+1/2, -x+1/2, z'
'-y+1/2, x+1/2, z'

'x+1/2, z+1/2, -y'
'-x+1/2, z+1/2, y'
'-x+1/2, -z+1/2, -y'
'x+1/2, -z+1/2, y'
'z+1/2, y+1/2, -x'
'z+1/2, -y+1/2, x'
'-z+1/2, y+1/2, x'
'-z+1/2, -y+1/2, -x'
'-x, -y, -z'
'x, y, -z'
'x, -y, z'
'-x, y, z'
'-z, -x, -y'
'-z, x, y'
'z, x, -y'
'z, -x, y'
'-y, -z, -x'
'y, -z, x'
'-y, z, x'
'y, z, -x'
'-y, -x, z'
'y, x, z'
'-y, x, -z'
'y, -x, -z'
'-x, -z, y'
'x, -z, -y'
'x, z, y'
'-x, z, -y'
'-z, -y, x'
'-z, y, -x'
'z, -y, -x'
'z, y, x'
'-x, -y+1/2, -z+1/2'
'x, y+1/2, -z+1/2'
'x, -y+1/2, z+1/2'
'-x, y+1/2, z+1/2'
'-z, -x+1/2, -y+1/2'
'-z, x+1/2, y+1/2'
'z, x+1/2, -y+1/2'
'z, -x+1/2, y+1/2'
'-y, -z+1/2, -x+1/2'
'y, -z+1/2, x+1/2'
'-y, z+1/2, x+1/2'
'y, z+1/2, -x+1/2'

'-y, -x+1/2, z+1/2'

'y, x+1/2, z+1/2'

'-y, x+1/2, -z+1/2'

'y, -x+1/2, -z+1/2'

'-x, -z+1/2, y+1/2'

'x, -z+1/2, -y+1/2'

'x, z+1/2, y+1/2'

'-x, z+1/2, -y+1/2'

'-z, -y+1/2, x+1/2'

'-z, y+1/2, -x+1/2'

'z, -y+1/2, -x+1/2'

'z, y+1/2, x+1/2'

'-x+1/2, -y, -z+1/2'

'x+1/2, y, -z+1/2'

'x+1/2, -y, z+1/2'

'-x+1/2, y, z+1/2'

'-z+1/2, -x, -y+1/2'

'-z+1/2, x, y+1/2'

'z+1/2, x, -y+1/2'

'z+1/2, -x, y+1/2'

'-y+1/2, -z, -x+1/2'

'y+1/2, -z, x+1/2'

'-y+1/2, z, x+1/2'

'y+1/2, z, -x+1/2'

'-y+1/2, -x, z+1/2'

'y+1/2, x, z+1/2'

'-y+1/2, x, -z+1/2'

'y+1/2, -x, -z+1/2'

'-x+1/2, -z, y+1/2'

'x+1/2, -z, -y+1/2'

'x+1/2, z, y+1/2'

'-x+1/2, z, -y+1/2'

'-z+1/2, -y, x+1/2'

'-z+1/2, y, -x+1/2'

'z+1/2, -y, -x+1/2'

'z+1/2, y, x+1/2'

'-x+1/2, -y+1/2, -z'

'x+1/2, y+1/2, -z'

'x+1/2, -y+1/2, z'

'-x+1/2, y+1/2, z'

'-z+1/2, -x+1/2, -y'

'-z+1/2, x+1/2, y'

'z+1/2, x+1/2, -y'

'z+1/2, -x+1/2, y'

'-y+1/2, -z+1/2, -x'
'y+1/2, -z+1/2, x'
'-y+1/2, z+1/2, x'
'y+1/2, z+1/2, -x'
'-y+1/2, -x+1/2, z'
'y+1/2, x+1/2, z'
'-y+1/2, x+1/2, -z'
'y+1/2, -x+1/2, -z'
'-x+1/2, -z+1/2, y'
'x+1/2, -z+1/2, -y'
'x+1/2, z+1/2, y'
'-x+1/2, z+1/2, -y'
'-z+1/2, -y+1/2, x'
'-z+1/2, y+1/2, -x'
'z+1/2, -y+1/2, -x'
'z+1/2, y+1/2, x'

_cell_length_a 44.5993(3)
_cell_length_b 44.5993(3)
_cell_length_c 44.5993(3)
_cell_angle_alpha 90.00
_cell_angle_beta 90.00
_cell_angle_gamma 90.00
_cell_volume 88712.4(18)
_cell_formula_units_Z 8
_cell_measurement_temperature 293(2)
_cell_measurement_reflns_used ?
_cell_measurement_theta_min ?
_cell_measurement_theta_max ?

_exptl_crystal_description polyhedra
_exptl_crystal_colour green
_exptl_crystal_size_max 0.20
_exptl_crystal_size_mid 0.20
_exptl_crystal_size_min 0.20
_exptl_crystal_density_meas ?
_exptl_crystal_density_diffrn 1.013
_exptl_crystal_density_method 'not measured'
_exptl_crystal_F_000 26072
_exptl_absorpt_coefficient_mu 1.507
_exptl_absorpt_correction_type multi-scan
_exptl_absorpt_correction_T_min 0.740
_exptl_absorpt_correction_T_max 0.747
_exptl_absorpt_process_details 'SADABS; (Sheldrick, 2003)'

```
_exptl_special_details
;
 ?
;

_diffrn_ambient_temperature           293(2)
_diffrn_radiation_wavelength          0.71073
_diffrn_radiation_type                MoK\a
_diffrn_radiation_source              'fine-focus sealed tube'
_diffrn_radiation_monochromator       graphite
_diffrn_measurement_device_type       ?
_diffrn_measurement_method            ?
_diffrn_detector_area_resol_mean      ?
_diffrn_standards_number              ?
_diffrn_standards_interval_count      ?
_diffrn_standards_interval_time       ?
_diffrn_standards_decay_%             ?
_diffrn_reflns_number                 18345
_diffrn_reflns_av_R_equivalents       0.0661
_diffrn_reflns_av_sigmaI/netI         0.0433
_diffrn_reflns_limit_h_min            -32
_diffrn_reflns_limit_h_max            52
_diffrn_reflns_limit_k_min            -43
_diffrn_reflns_limit_k_max            9
_diffrn_reflns_limit_l_min            -46
_diffrn_reflns_limit_l_max            33
_diffrn_reflns_theta_min              3.00
_diffrn_reflns_theta_max              24.99
_reflns_number_total                  3810
_reflns_number_gt                     3026
_reflns_threshold_expression          >2sigma(I)

_computing_data_collection            'Bruker FRAMBO'
_computing_cell_refinement            'Bruker FRAMBO'
_computing_data_reduction             'Bruker SHELXTL'
_computing_structure_solution         'Bruker SHELXTL'
_computing_structure_refinement       'SHELXL-97 (Sheldrick, 1997)'
_computing_molecular_graphics         'Bruker SHELXTL'
_computing_publication_material       'Bruker SHELXTL'

_refine_special_details
;
 Refinement of F^2^ against ALL reflections.  The weighted R-factor wR and
```

goodness of fit S are based on F^2^, conventional R-factors R are based
on F, with F set to zero for negative F^2^. The threshold expression of
F^2^ > 2sigma(F^2^) is used only for calculating R-factors(gt) etc. and is
not relevant to the choice of reflections for refinement. R-factors based
on F^2^ are statistically about twice as large as those based on F, and R-
factors based on ALL data will be even larger.
;

```
_refine_ls_structure_factor_coef    Fsqd
_refine_ls_matrix_type              full
_refine_ls_weighting_scheme         calc
_refine_ls_weighting_details
 'calc w=1/[\s^2^(Fo^2^)+(0.1804P)^2^+687.0303P] where P=(Fo^2^+2Fc^2^)/3'
_atom_sites_solution_primary        direct
_atom_sites_solution_secondary      difmap
_atom_sites_solution_hydrogens      geom
_refine_ls_hydrogen_treatment       constr
_refine_ls_extinction_method        SHELXL
_refine_ls_extinction_coef          0.000020(9)
_refine_ls_extinction_expression
 'Fc^*^=kFc[1+0.001xFc^2^\l^3^/sin(2\q)]^-1/4^'
_refine_ls_number_reflns            3810
_refine_ls_number_parameters        148
_refine_ls_number_restraints        0
_refine_ls_R_factor_all             0.0997
_refine_ls_R_factor_gt              0.0875
_refine_ls_wR_factor_ref            0.2696
_refine_ls_wR_factor_gt             0.2584
_refine_ls_goodness_of_fit_ref      1.041
_refine_ls_restrained_S_all         1.041
_refine_ls_shift/su_max             0.000
_refine_ls_shift/su_mean            0.000

loop_
 _atom_site_label
 _atom_site_type_symbol
 _atom_site_fract_x
 _atom_site_fract_y
 _atom_site_fract_z
 _atom_site_U_iso_or_equiv
 _atom_site_adp_type
 _atom_site_occupancy
 _atom_site_symmetry_multiplicity
 _atom_site_calc_flag
```

```
  _atom_site_refinement_flags
  _atom_site_disorder_assembly
  _atom_site_disorder_group
Cu1 Cu 0.168543(16) 0.168543(16) 0.0000 0.0239(4) Uani 1 4 d S . .
Cu2 Cu 0.126518(16) 0.126518(16) 0.0000 0.0243(4) Uani 1 4 d S . .
Cu3 Cu 0.32380(4) 0.12316(4) 0.17620(4) 0.1033(9) Uani 1 2 d S . .
O1 O 0.15135(9) 0.10802(9) 0.03108(9) 0.0439(10) Uani 1 1 d . . .
O2 O 0.18690(9) 0.14347(9) 0.03101(9) 0.0422(10) Uani 1 1 d . . .
O3 O 0.3348(2) 0.1652(2) 0.1652(2) 0.076(4) Uani 1 6 d S . .
C1 C 0.2571(3) 0.1168(2) 0.1168(2) 0.064(3) Uani 1 2 d S . .
C2 C 0.22913(19) 0.10368(16) 0.10368(16) 0.049(2) Uani 1 2 d S . .
C3 C 0.21623(14) 0.11636(13) 0.07836(14) 0.0470(15) Uani 1 1 d . . .
H3 H 0.2253 0.1327 0.0692 0.056 Uiso 1 1 calc R . .
C4 C 0.19003(12) 0.10476(12) 0.06677(12) 0.0379(13) Uani 1 1 d . . .
C5 C 0.17726(17) 0.07999(12) 0.07999(12) 0.0371(16) Uani 1 2 d S . .
H5 H 0.1597 0.0721 0.0721 0.044 Uiso 1 2 calc SR . .
C6 C 0.17511(12) 0.12013(12) 0.04084(12) 0.0351(12) Uani 1 1 d . . .
N1 N 0.27022(15) 0.10595(15) 0.14066(16) 0.084(3) Uani 1 1 d . . .
N2 N 0.29273(15) 0.12504(15) 0.14520(16) 0.083(2) Uani 1 1 d . . .
O1W O 0.20268(13) 0.20268(13) 0.0000 0.076(3) Uani 1 4 d S . .
O2W O 0.09217(14) 0.09217(14) 0.0000 0.133(7) Uani 1 4 d S . .
Mo1 Mo 0.30621(3) 0.24794(4) 0.19379(3) 0.1298(8) Uani 1 2 d S . .
P1 P 0.2500 0.2500 0.2500 0.088(3) Uani 1 24 d S . .
O4 O 0.2696(3) 0.2304(3) 0.2304(3) 0.131(7) Uani 1 6 d S . .
O5 O 0.3154(3) 0.2745(2) 0.2255(2) 0.129(4) Uani 1 2 d S . .
O6 O 0.3323(2) 0.2561(4) 0.1677(2) 0.149(5) Uani 1 2 d S . .
O7 O 0.2830(2) 0.2170(2) 0.1732(3) 0.133(4) Uani 1 2 d S . .
O9A O 0.2935(4) 0.1014(6) 0.2065(4) 0.123(7) Uiso 0.50 2 d SP . .
O9B O 0.3100(5) 0.0835(6) 0.1900(5) 0.139(9) Uiso 0.50 2 d SP . .
O10A O 0.3655(7) 0.1098(10) 0.1345(7) 0.26(2) Uiso 0.50 2 d SP . .
O10B O 0.3490(6) 0.0872(10) 0.1510(6) 0.198(14) Uiso 0.50 2 d SP . .

loop_
  _atom_site_aniso_label
  _atom_site_aniso_U_11
  _atom_site_aniso_U_22
  _atom_site_aniso_U_33
  _atom_site_aniso_U_23
  _atom_site_aniso_U_13
  _atom_site_aniso_U_12
Cu1 0.0264(5) 0.0264(5) 0.0188(6) 0.000 0.000 -0.0094(4)
Cu2 0.0268(5) 0.0268(5) 0.0191(6) 0.000 0.000 -0.0097(4)
Cu3 0.1068(10) 0.0964(12) 0.1068(10) 0.0667(9) -0.0813(11) -0.0667(9)
O1 0.049(2) 0.043(2) 0.040(2) 0.0126(17) -0.0178(18) -0.0106(17)
```

O2 0.042(2) 0.049(2) 0.036(2) 0.0203(17) -0.0122(16) -0.0136(18)
O3 0.076(4) 0.076(4) 0.076(4) 0.039(5) -0.039(5) -0.039(5)
C1 0.072(7) 0.060(4) 0.060(4) 0.036(5) -0.029(4) -0.029(4)
C2 0.044(5) 0.051(3) 0.051(3) 0.028(4) -0.022(3) -0.022(3)
C3 0.048(3) 0.043(3) 0.051(3) 0.019(3) -0.012(3) -0.026(3)
C4 0.036(3) 0.045(3) 0.033(3) 0.011(2) -0.012(2) -0.007(2)
C5 0.041(4) 0.035(2) 0.035(2) 0.007(3) -0.009(2) -0.009(2)
C6 0.035(3) 0.041(3) 0.029(3) 0.005(2) -0.003(2) -0.004(2)
N1 0.078(4) 0.081(4) 0.093(5) 0.058(4) -0.057(4) -0.050(4)
N2 0.083(4) 0.079(4) 0.088(5) 0.051(4) -0.058(4) -0.051(4)
O1W 0.065(4) 0.065(4) 0.096(8) 0.000 0.000 -0.053(5)
O2W 0.049(4) 0.049(4) 0.30(2) 0.000 0.000 -0.036(5)
Mo1 0.1350(10) 0.1195(13) 0.1350(10) 0.0047(7) 0.0162(11) -0.0047(7)
P1 0.088(3) 0.088(3) 0.088(3) 0.000 0.000 0.000
O4 0.131(7) 0.131(7) 0.131(7) 0.009(8) -0.009(8) -0.009(8)
O5 0.137(10) 0.124(6) 0.124(6) 0.004(8) 0.015(5) -0.015(5)
O6 0.136(7) 0.174(14) 0.136(7) 0.000(6) 0.037(9) 0.000(6)
O7 0.138(7) 0.138(7) 0.122(9) -0.008(5) 0.008(5) 0.009(8)

_geom_special_details
;
All esds (except the esd in the dihedral angle between two l.s. planes)
are estimated using the full covariance matrix. The cell esds are taken
into account individually in the estimation of esds in distances, angles
and torsion angles; correlations between esds in cell parameters are only
used when they are defined by crystal symmetry. An approximate (isotropic)
treatment of cell esds is used for estimating esds involving l.s. planes.
;

loop_
_geom_bond_atom_site_label_1
_geom_bond_atom_site_label_2
_geom_bond_distance
_geom_bond_site_symmetry_2
_geom_bond_publ_flag
Cu1 O2 1.958(4) 98 ?
Cu1 O2 1.958(4) . ?
Cu1 O2 1.958(4) 13 ?
Cu1 O2 1.958(4) 110 ?
Cu1 O1W 2.153(8) . ?
Cu1 Cu2 2.6506(15) . ?
Cu2 O1 1.957(4) . ?
Cu2 O1 1.957(4) 98 ?
Cu2 O1 1.957(4) 110 ?

Cu2 O1 1.957(4) 13 ?
Cu2 O2W 2.166(9) . ?
Cu3 N2 1.959(5) 166 ?
Cu3 N2 1.959(5) . ?
Cu3 O9B 1.97(3) . ?
Cu3 O3 2.000(4) . ?
Cu3 O9A 2.14(3) . ?
Cu3 O10B 2.26(4) . ?
O1 C6 1.266(7) . ?
O2 C6 1.246(6) . ?
O3 Cu3 2.000(4) 58 ?
O3 Cu3 2.000(4) 79 ?
C1 N1 1.309(7) . ?
C1 N1 1.309(7) 115 ?
C1 C2 1.495(12) . ?
C2 C3 1.388(7) 115 ?
C2 C3 1.388(7) . ?
C3 C4 1.379(7) . ?
C4 C5 1.376(6) . ?
C4 C6 1.500(7) . ?
C5 C4 1.376(6) 115 ?
N1 N2 1.332(7) . ?
N2 N2 1.272(11) 115 ?
Mo1 O6 1.683(13) . ?
Mo1 O5 1.890(4) . ?
Mo1 O5 1.890(4) 35 ?
Mo1 O7 1.954(7) 79 ?
Mo1 O7 1.954(7) . ?
Mo1 O4 2.437(15) . ?
P1 O4 1.51(3) 28 ?
P1 O4 1.51(3) . ?
P1 O4 1.51(3) 74 ?
P1 O4 1.51(3) 51 ?
O4 Mo1 2.437(15) 79 ?
O4 Mo1 2.437(15) 58 ?
O5 Mo1 1.890(4) 56 ?
O7 Mo1 1.954(7) 58 ?
O9A O9B 1.31(3) . ?
O10A O10B 1.45(5) . ?
O10A O10A 1.56(7) 58 ?
O10A O10A 1.56(7) 79 ?

loop_
_geom_angle_atom_site_label_1

_geom_angle_atom_site_label_2
_geom_angle_atom_site_label_3
_geom_angle
_geom_angle_site_symmetry_1
_geom_angle_site_symmetry_3
_geom_angle_publ_flag
O2 Cu1 O2 89.9(3) 98 . ?
O2 Cu1 O2 88.8(3) 98 13 ?
O2 Cu1 O2 167.6(2) . 13 ?
O2 Cu1 O2 167.6(2) 98 110 ?
O2 Cu1 O2 88.8(3) . 110 ?
O2 Cu1 O2 89.9(3) 13 110 ?
O2 Cu1 O1W 96.21(11) 98 . ?
O2 Cu1 O1W 96.21(11) . . ?
O2 Cu1 O1W 96.21(11) 13 . ?
O2 Cu1 O1W 96.21(11) 110 . ?
O2 Cu1 Cu2 83.79(11) 98 . ?
O2 Cu1 Cu2 83.79(11) . . ?
O2 Cu1 Cu2 83.79(11) 13 . ?
O2 Cu1 Cu2 83.79(11) 110 . ?
O1W Cu1 Cu2 180.0(2) . . ?
O1 Cu2 O1 90.2(3) . 98 ?
O1 Cu2 O1 88.6(3) . 110 ?
O1 Cu2 O1 168.3(2) 98 110 ?
O1 Cu2 O1 168.3(2) . 13 ?
O1 Cu2 O1 88.6(3) 98 13 ?
O1 Cu2 O1 90.2(3) 110 13 ?
O1 Cu2 O2W 95.86(11) . . ?
O1 Cu2 O2W 95.86(11) 98 . ?
O1 Cu2 O2W 95.86(11) 110 . ?
O1 Cu2 O2W 95.86(11) 13 . ?
O1 Cu2 Cu1 84.14(11) . . ?
O1 Cu2 Cu1 84.14(11) 98 . ?
O1 Cu2 Cu1 84.14(11) 110 . ?
O1 Cu2 Cu1 84.14(11) 13 . ?
O2W Cu2 Cu1 180.0(2) . . ?
N2 Cu3 N2 175.1(4) 166 . ?
N2 Cu3 O9B 92.18(16) 166 . ?
N2 Cu3 O9B 92.18(16) . . ?
N2 Cu3 O3 87.71(17) 166 . ?
N2 Cu3 O3 87.71(17) . . ?
O9B Cu3 O3 174.2(10) . . ?
N2 Cu3 O9A 91.1(2) 166 . ?
N2 Cu3 O9A 91.1(2) . . ?

O9B Cu3 O9A 36.9(10) . . ?
O3 Cu3 O9A 137.3(8) . . ?
N2 Cu3 O10B 91.8(3) 166 . ?
N2 Cu3 O10B 91.8(3) . . ?
O9B Cu3 O10B 70.9(13) . . ?
O3 Cu3 O10B 114.9(12) . . ?
O9A Cu3 O10B 107.8(13) . . ?
C6 O1 Cu2 122.5(3) . . ?
C6 O2 Cu1 123.3(3) . . ?
Cu3 O3 Cu3 113.6(4) 58 . ?
Cu3 O3 Cu3 113.6(4) 58 79 ?
Cu3 O3 Cu3 113.6(4) . 79 ?
N1 C1 N1 113.5(8) . 115 ?
N1 C1 C2 123.2(4) . . ?
N1 C1 C2 123.2(4) 115 . ?
C3 C2 C3 119.5(7) 115 . ?
C3 C2 C1 120.3(3) 115 . ?
C3 C2 C1 120.3(3) . . ?
C4 C3 C2 120.2(5) . . ?
C5 C4 C3 119.5(5) . . ?
C5 C4 C6 120.9(5) . . ?
C3 C4 C6 119.6(5) . . ?
C4 C5 C4 121.1(7) 115 . ?
O2 C6 O1 126.1(5) . . ?
O2 C6 C4 117.8(5) . . ?
O1 C6 C4 116.2(4) . . ?
C1 N1 N2 103.1(5) . . ?
N2 N2 N1 110.1(3) 115 . ?
N2 N2 Cu3 121.96(16) 115 . ?
N1 N2 Cu3 127.8(4) . . ?
O6 Mo1 O5 103.4(6) . . ?
O6 Mo1 O5 103.4(6) . 35 ?
O5 Mo1 O5 86.2(8) . 35 ?
O6 Mo1 O7 101.1(5) . 79 ?
O5 Mo1 O7 86.7(6) . 79 ?
O5 Mo1 O7 155.4(5) 35 79 ?
O6 Mo1 O7 101.1(5) . . ?
O5 Mo1 O7 155.4(5) . . ?
O5 Mo1 O7 86.7(6) 35 . ?
O7 Mo1 O7 90.0(8) 79 . ?
O6 Mo1 O4 173.7(8) . . ?
O5 Mo1 O4 81.1(5) . . ?
O5 Mo1 O4 81.1(5) 35 . ?
O7 Mo1 O4 74.5(5) 79 . ?

O7 Mo1 O4 74.5(5) . . ?
O4 P1 O4 109.471(8) 28 . ?
O4 P1 O4 109.471(3) 28 74 ?
O4 P1 O4 109.471(1) . 74 ?
O4 P1 O4 109.5 28 51 ?
O4 P1 O4 109.471(3) . 51 ?
O4 P1 O4 109.471(5) 74 51 ?
P1 O4 Mo1 126.0(5) . 79 ?
P1 O4 Mo1 126.0(5) . 58 ?
Mo1 O4 Mo1 88.9(7) 79 58 ?
P1 O4 Mo1 126.0(5) . . ?
Mo1 O4 Mo1 88.9(7) 79 . ?
Mo1 O4 Mo1 88.9(7) 58 . ?
Mo1 O5 Mo1 152.9(8) . 56 ?
Mo1 O7 Mo1 121.8(7) . 58 ?
O9B O9A Cu3 64.4(17) . . ?
O9A O9B Cu3 78.7(18) . . ?
O10B O10A O10A 148.4(8) . 58 ?
O10B O10A O10A 148.4(8) . 79 ?
O10A O10A O10A 60.000(13) 58 79 ?
O10A O10B Cu3 91(3) . . ?

```
_diffrn_measured_fraction_theta_max        0.990
_diffrn_reflns_theta_full                  24.99
_diffrn_measured_fraction_theta_full      0.990
_refine_diff_density_max        1.411
_refine_diff_density_min      -1.256
_refine_diff_density_rms        0.132
# SQUEEZE RESULTS (APPEND TO CIF)
loop_
   _platon_squeeze_void_nr
   _platon_squeeze_void_average_x
   _platon_squeeze_void_average_y
   _platon_squeeze_void_average_z
   _platon_squeeze_void_volume
   _platon_squeeze_void_count_electrons
         1     -0.005     -0.012     -0.005    56810.6     332.5
_platon_squeeze_details
; ?
;
```

```
data_HLJU-2

_audit_creation_method            SHELXL-97
_chemical_name_systematic
;
 ?
;
_chemical_name_common              ?
_chemical_melting_point           ?
_chemical_formula_moiety          ' 4(C27 H9 Cu6 N12 O22), Mo12 O40 Si '
_chemical_formula_sum
 ' C108 H36 Cu24 Mo12 N48 O128 Si '
_chemical_formula_weight          6758.42

loop_
 _atom_type_symbol
 _atom_type_description
 _atom_type_scat_dispersion_real
 _atom_type_scat_dispersion_imag
 _atom_type_scat_source
 'C'  'C'   0.0033   0.0016
 'International Tables Vol C Tables 4.2.6.8 and 6.1.1.4'
 'H'  'H'   0.0000   0.0000
 'International Tables Vol C Tables 4.2.6.8 and 6.1.1.4'
 'O'  'O'   0.0106   0.0060
 'International Tables Vol C Tables 4.2.6.8 and 6.1.1.4'
 'Si'  'Si'   0.0817   0.0704
 'International Tables Vol C Tables 4.2.6.8 and 6.1.1.4'
 'Cu'  'Cu'   0.3201   1.2651
 'International Tables Vol C Tables 4.2.6.8 and 6.1.1.4'
 'Mo'  'Mo'  -1.6832   0.6857
 'International Tables Vol C Tables 4.2.6.8 and 6.1.1.4'
 'N'  'N'   0.0061   0.0033
 'International Tables Vol C Tables 4.2.6.8 and 6.1.1.4'

_symmetry_cell_setting            cubic
_symmetry_space_group_name_H-M    'F m -3 m '
_symmetry_space_group_name_Hall   '-F 4 2 3  '

loop_
 _symmetry_equiv_pos_as_xyz
 'x, y, z'
```

'-x, -y, z'
'x, -y, -z'
'-x, y, -z'
'z, x, y'
'y, z, x'
'-z, -x, y'
'-y, z, -x'
'z, -x, -y'
'-y, -z, x'
'-z, x, -y'
'y, -z, -x'
'-y, -x, -z'
'y, x, -z'
'y, -x, z'
'-y, x, z'
'-x, -z, -y'
'-z, -y, -x'
'x, z, -y'
'-z, y, x'
'x, -z, y'
'z, y, -x'
'-x, z, y'
'z, -y, x'
'x, y+1/2, z+1/2'
'-x, -y+1/2, z+1/2'
'x, -y+1/2, -z+1/2'
'-x, y+1/2, -z+1/2'
'z, x+1/2, y+1/2'
'y, z+1/2, x+1/2'
'-z, -x+1/2, y+1/2'
'-y, z+1/2, -x+1/2'
'z, -x+1/2, -y+1/2'
'-y, -z+1/2, x+1/2'
'-z, x+1/2, -y+1/2'
'y, -z+1/2, -x+1/2'
'-y, -x+1/2, -z+1/2'
'y, x+1/2, -z+1/2'
'y, -x+1/2, z+1/2'
'-y, x+1/2, z+1/2'
'-x, -z+1/2, -y+1/2'
'-z, -y+1/2, -x+1/2'
'x, z+1/2, -y+1/2'
'-z, y+1/2, x+1/2'
'x, -z+1/2, y+1/2'

'z, y+1/2, -x+1/2'
'-x, z+1/2, y+1/2'
'z, -y+1/2, x+1/2'
'x+1/2, y, z+1/2'
'-x+1/2, -y, z+1/2'
'x+1/2, -y, -z+1/2'
'-x+1/2, y, -z+1/2'
'z+1/2, x, y+1/2'
'y+1/2, z, x+1/2'
'-z+1/2, -x, y+1/2'
'-y+1/2, z, -x+1/2'
'z+1/2, -x, -y+1/2'
'-y+1/2, -z, x+1/2'
'-z+1/2, x, -y+1/2'
'y+1/2, -z, -x+1/2'
'-y+1/2, -x, -z+1/2'
'y+1/2, x, -z+1/2'
'y+1/2, -x, z+1/2'
'-y+1/2, x, z+1/2'
'-x+1/2, -z, -y+1/2'
'-z+1/2, -y, -x+1/2'
'x+1/2, z, -y+1/2'
'-z+1/2, y, x+1/2'
'x+1/2, -z, y+1/2'
'z+1/2, y, -x+1/2'
'-x+1/2, z, y+1/2'
'z+1/2, -y, x+1/2'
'x+1/2, y+1/2, z'
'-x+1/2, -y+1/2, z'
'x+1/2, -y+1/2, -z'
'-x+1/2, y+1/2, -z'
'z+1/2, x+1/2, y'
'y+1/2, z+1/2, x'
'-z+1/2, -x+1/2, y'
'-y+1/2, z+1/2, -x'
'z+1/2, -x+1/2, -y'
'-y+1/2, -z+1/2, x'
'-z+1/2, x+1/2, -y'
'y+1/2, -z+1/2, -x'
'-y+1/2, -x+1/2, -z'
'y+1/2, x+1/2, -z'
'y+1/2, -x+1/2, z'
'-y+1/2, x+1/2, z'
'-x+1/2, -z+1/2, -y'

'-z+1/2, -y+1/2, -x'
'x+1/2, z+1/2, -y'
'-z+1/2, y+1/2, x'
'x+1/2, -z+1/2, y'
'z+1/2, y+1/2, -x'
'-x+1/2, z+1/2, y'
'z+1/2, -y+1/2, x'
'-x, -y, -z'
'x, y, -z'
'-x, y, z'
'x, -y, z'
'-z, -x, -y'
'-y, -z, -x'
'z, x, -y'
'y, -z, x'
'-z, x, y'
'y, z, -x'
'z, -x, y'
'-y, z, x'
'y, x, z'
'-y, -x, z'
'-y, x, -z'
'y, -x, -z'
'x, z, y'
'z, y, x'
'-x, -z, y'
'z, -y, -x'
'-x, z, -y'
'-z, -y, x'
'x, -z, -y'
'-z, y, -x'
'-x, -y+1/2, -z+1/2'
'x, y+1/2, -z+1/2'
'-x, y+1/2, z+1/2'
'x, -y+1/2, z+1/2'
'-z, -x+1/2, -y+1/2'
'-y, -z+1/2, -x+1/2'
'z, x+1/2, -y+1/2'
'y, -z+1/2, x+1/2'
'-z, x+1/2, y+1/2'
'y, z+1/2, -x+1/2'
'z, -x+1/2, y+1/2'
'-y, z+1/2, x+1/2'
'y, x+1/2, z+1/2'

'-y, -x+1/2, z+1/2'
'-y, x+1/2, -z+1/2'
'y, -x+1/2, -z+1/2'
'x, z+1/2, y+1/2'
'z, y+1/2, x+1/2'
'-x, -z+1/2, y+1/2'
'z, -y+1/2, -x+1/2'
'-x, z+1/2, -y+1/2'
'-z, -y+1/2, x+1/2'
'x, -z+1/2, -y+1/2'
'-z, y+1/2, -x+1/2'
'-x+1/2, -y, -z+1/2'
'x+1/2, y, -z+1/2'
'-x+1/2, y, z+1/2'
'x+1/2, -y, z+1/2'
'-z+1/2, -x, -y+1/2'
'-y+1/2, -z, -x+1/2'
'z+1/2, x, -y+1/2'
'y+1/2, -z, x+1/2'
'-z+1/2, x, y+1/2'
'y+1/2, z, -x+1/2'
'z+1/2, -x, y+1/2'
'-y+1/2, z, x+1/2'
'y+1/2, x, z+1/2'
'-y+1/2, -x, z+1/2'
'-y+1/2, x, -z+1/2'
'y+1/2, -x, -z+1/2'
'x+1/2, z, y+1/2'
'z+1/2, y, x+1/2'
'-x+1/2, -z, y+1/2'
'z+1/2, -y, -x+1/2'
'-x+1/2, z, -y+1/2'
'-z+1/2, -y, x+1/2'
'x+1/2, -z, -y+1/2'
'-z+1/2, y, -x+1/2'
'-x+1/2, -y+1/2, -z'
'x+1/2, y+1/2, -z'
'-x+1/2, y+1/2, z'
'x+1/2, -y+1/2, z'
'-z+1/2, -x+1/2, -y'
'-y+1/2, -z+1/2, -x'
'z+1/2, x+1/2, -y'
'y+1/2, -z+1/2, x'
'-z+1/2, x+1/2, y'

'y+1/2, z+1/2, -x'
'z+1/2, -x+1/2, y'
'-y+1/2, z+1/2, x'
'y+1/2, x+1/2, z'
'-y+1/2, -x+1/2, z'
'-y+1/2, x+1/2, -z'
'y+1/2, -x+1/2, -z'
'x+1/2, z+1/2, y'
'z+1/2, y+1/2, x'
'-x+1/2, -z+1/2, y'
'z+1/2, -y+1/2, -x'
'-x+1/2, z+1/2, -y'
'-z+1/2, -y+1/2, x'
'x+1/2, -z+1/2, -y'
'-z+1/2, y+1/2, -x'

_cell_length_a 44.614(5)
_cell_length_b 44.614(5)
_cell_length_c 44.614(5)
_cell_angle_alpha 90.000(5)
_cell_angle_beta 90.000(5)
_cell_angle_gamma 90.000(5)
_cell_volume 88800(30)
_cell_formula_units_Z 8
_cell_measurement_temperature 293(2)
_cell_measurement_reflns_used ?
_cell_measurement_theta_min ?
_cell_measurement_theta_max ?

_exptl_crystal_description polyhedra
_exptl_crystal_colour green
_exptl_crystal_size_max 0.20
_exptl_crystal_size_mid 0.20
_exptl_crystal_size_min 0.20
_exptl_crystal_density_meas ?
_exptl_crystal_density_diffrn 1.011
_exptl_crystal_density_method 'not measured'
_exptl_crystal_F_000 26064
_exptl_absorpt_coefficient_mu 1.504
_exptl_absorpt_correction_type multi-scan
_exptl_absorpt_correction_T_min 0.740
_exptl_absorpt_correction_T_max 0.748
_exptl_absorpt_process_details 'SADABS; (Sheldrick, 2003)'

```
_exptl_special_details
;
 ?
;

_diffrn_ambient_temperature           293(2)
_diffrn_radiation_wavelength          0.71069
_diffrn_radiation_type                MoK\a
_diffrn_radiation_source              'fine-focus sealed tube'
_diffrn_radiation_monochromator       graphite
_diffrn_measurement_device_type       ?
_diffrn_measurement_method            ?
_diffrn_detector_area_resol_mean      ?
_diffrn_standards_number              ?
_diffrn_standards_interval_count      ?
_diffrn_standards_interval_time       ?
_diffrn_standards_decay_%             ?
_diffrn_reflns_number                 16911
_diffrn_reflns_av_R_equivalents       0.0867
_diffrn_reflns_av_sigmaI/netI         0.0675
_diffrn_reflns_limit_h_min            -36
_diffrn_reflns_limit_h_max            12
_diffrn_reflns_limit_k_min            -15
_diffrn_reflns_limit_k_max            53
_diffrn_reflns_limit_l_min            -48
_diffrn_reflns_limit_l_max            23
_diffrn_reflns_theta_min              2.99
_diffrn_reflns_theta_max              24.98
_reflns_number_total                  3810
_reflns_number_gt                     2531
_reflns_threshold_expression          >2sigma(I)

_computing_data_collection            'Bruker FRAMBO'
_computing_cell_refinement            'Bruker FRAMBO'
_computing_data_reduction             'Bruker SHELXTL'
_computing_structure_solution         'Bruker SHELXTL'
_computing_structure_refinement       'SHELXL-97 (Sheldrick, 1997)'
_computing_molecular_graphics         'Bruker SHELXTL'
_computing_publication_material       'Bruker SHELXTL'

_refine_special_details
;
 Refinement of F^2^ against ALL reflections.  The weighted R-factor wR and
 goodness of fit S are based on F^2^, conventional R-factors R are based
```

```
  on F, with F set to zero for negative F^2^. The threshold expression of
  F^2^ > 2sigma(F^2^) is used only for calculating R-factors(gt) etc. and is
  not relevant to the choice of reflections for refinement.   R-factors based
  on F^2^ are statistically about twice as large as those based on F, and R-
  factors based on ALL data will be even larger.
;

_refine_ls_structure_factor_coef   Fsqd
_refine_ls_matrix_type             full
_refine_ls_weighting_scheme        calc
_refine_ls_weighting_details
 'calc w=1/[\s^2^(Fo^2^)+(0.1311P)^2^+0.0000P] where P=(Fo^2^+2Fc^2^)/3'
_atom_sites_solution_primary       direct
_atom_sites_solution_secondary     difmap
_atom_sites_solution_hydrogens     geom
_refine_ls_hydrogen_treatment      CONSTR
_refine_ls_extinction_method       none
_refine_ls_extinction_coef         ?
_refine_ls_number_reflns           3810
_refine_ls_number_parameters       147
_refine_ls_number_restraints       0
_refine_ls_R_factor_all            0.0967
_refine_ls_R_factor_gt             0.0710
_refine_ls_wR_factor_ref           0.2188
_refine_ls_wR_factor_gt            0.2017
_refine_ls_goodness_of_fit_ref     0.990
_refine_ls_restrained_S_all        0.990
_refine_ls_shift/su_max            0.000
_refine_ls_shift/su_mean           0.000

loop_
 _atom_site_label
 _atom_site_type_symbol
 _atom_site_fract_x
 _atom_site_fract_y
 _atom_site_fract_z
 _atom_site_U_iso_or_equiv
 _atom_site_adp_type
 _atom_site_occupancy
 _atom_site_symmetry_multiplicity
 _atom_site_calc_flag
 _atom_site_refinement_flags
 _atom_site_disorder_assembly
 _atom_site_disorder_group
```

Cu2 Cu 0.168383(17) 0.168383(17) 0.0000 0.0263(3) Uani 1 4 d S . .
Cu1 Cu 0.126469(17) 0.126469(17) 0.0000 0.0272(3) Uani 1 4 d S . .
Cu3 Cu 0.17617(3) 0.32383(3) -0.12286(3) 0.0845(6) Uani 1 2 d S . .
Mo1 Mo 0.19440(2) 0.25295(3) -0.19440(2) 0.1119(6) Uani 1 2 d S . .
O5 O 0.14347(8) 0.18687(8) -0.03088(9) 0.0447(10) Uani 1 1 d . . .
O3 O 0.10798(8) 0.15137(9) -0.03085(8) 0.0467(10) Uani 1 1 d . . .
O1 O 0.20279(13) 0.20279(13) 0.0000 0.077(3) Uani 1 4 d S . .
N1 N 0.14507(13) 0.29264(12) -0.12482(12) 0.0699(18) Uani 1 1 d . . .
C1 C 0.11995(12) 0.17485(12) -0.04087(11) 0.0360(12) Uani 1 1 d . . .
N2 N 0.14086(14) 0.27017(12) -0.10615(12) 0.0704(18) Uani 1 1 d . . .
O2 O 0.09252(14) 0.09252(14) 0.0000 0.131(6) Uani 1 4 d S . .
O12 O 0.16519(17) 0.33481(17) -0.16519(17) 0.065(3) Uani 1 6 d S . .
C3 C 0.10498(11) 0.18976(11) -0.06683(11) 0.0359(12) Uani 1 1 d . . .
O11 O 0.18420(18) 0.22594(15) -0.22594(15) 0.099(3) Uani 1 2 d S . .
O9 O 0.1735(2) 0.28364(15) -0.21636(15) 0.106(3) Uani 1 2 d S . .
O10 O 0.22919(18) 0.27081(18) -0.22919(18) 0.085(4) Uani 1 6 d S . .
O6 O 0.1999(4) 0.3001(4) -0.0939(4) 0.340(15) Uani 1 2 d S . .
C4 C 0.11638(12) 0.21607(13) -0.07867(13) 0.0448(14) Uani 1 1 d . . .
H4 H 0.1326 0.2254 -0.0694 0.054 Uiso 1 1 calc R . .
C5 C 0.10395(14) 0.22840(18) -0.10395(14) 0.049(2) Uani 1 2 d S . .
C2 C 0.08002(12) 0.17697(18) -0.08002(12) 0.0421(19) Uani 1 2 d S . .
H2 H 0.0720 0.1595 -0.0720 0.051 Uiso 1 2 calc SR . .
O8 O 0.16842(14) 0.2450(2) -0.16842(14) 0.123(3) Uani 1 2 d S . .
C6 C 0.11683(16) 0.2568(2) -0.11683(16) 0.056(2) Uani 1 2 d S . .
O7 O 0.1483(4) 0.3517(4) -0.0930(6) 0.368(14) Uani 1 2 d S . .
Si1 Si 0.2500 0.2500 -0.2500 0.067(3) Uani 1 24 d S . .

loop_
 _atom_site_aniso_label
 _atom_site_aniso_U_11
 _atom_site_aniso_U_22
 _atom_site_aniso_U_33
 _atom_site_aniso_U_23
 _atom_site_aniso_U_13
 _atom_site_aniso_U_12
Cu2 0.0281(4) 0.0281(4) 0.0227(6) 0.000 0.000 -0.0092(5)
Cu1 0.0293(4) 0.0293(4) 0.0228(6) 0.000 0.000 -0.0091(5)
Cu3 0.0881(7) 0.0881(7) 0.0774(9) 0.0430(7) -0.0430(7) -0.0581(8)
Mo1 0.1207(8) 0.0942(9) 0.1207(8) -0.0010(5) 0.0373(9) 0.0010(5)
O5 0.047(2) 0.042(2) 0.045(2) 0.0136(17) -0.0199(18) -0.0122(18)
O3 0.046(2) 0.049(2) 0.045(2) 0.0167(19) -0.0079(18) -0.0085(19)
O1 0.075(4) 0.075(4) 0.081(7) 0.000 0.000 -0.049(5)
N1 0.075(4) 0.064(3) 0.070(4) 0.034(3) -0.036(3) -0.040(3)
C1 0.044(3) 0.037(3) 0.027(3) 0.003(2) -0.002(2) -0.008(2)

N2 0.083(4) 0.065(4) 0.064(3) 0.032(3) -0.034(3) -0.039(3)
O2 0.061(4) 0.061(4) 0.272(19) 0.000 0.000 -0.040(6)
O12 0.065(3) 0.065(3) 0.065(3) 0.036(4) -0.036(4) -0.036(4)
C3 0.038(3) 0.036(3) 0.034(3) 0.010(2) -0.011(2) -0.009(2)
O11 0.077(5) 0.110(4) 0.110(4) -0.013(6) 0.002(3) -0.002(3)
O9 0.106(7) 0.106(4) 0.106(4) 0.016(5) -0.004(4) -0.004(4)
O10 0.085(4) 0.085(4) 0.085(4) 0.007(5) -0.007(5) -0.007(5)
O6 0.331(16) 0.331(16) 0.36(2) 0.280(18) -0.280(18) -0.268(18)
C4 0.033(3) 0.049(3) 0.052(3) 0.008(3) -0.011(3) -0.018(3)
C5 0.051(3) 0.045(5) 0.051(3) 0.017(3) -0.016(4) -0.017(3)
C2 0.039(3) 0.047(5) 0.039(3) 0.009(3) -0.001(3) -0.009(3)
O8 0.114(5) 0.139(8) 0.114(5) -0.010(4) 0.042(6) 0.010(4)
C6 0.053(3) 0.062(6) 0.053(3) 0.026(4) -0.032(4) -0.026(4)
O7 0.293(17) 0.293(17) 0.52(3) -0.12(2) 0.12(2) -0.248(19)
Si1 0.067(3) 0.067(3) 0.067(3) 0.000 0.000 0.000

_geom_special_details
;
All esds (except the esd in the dihedral angle between two l.s. planes)
are estimated using the full covariance matrix. The cell esds are taken
into account individually in the estimation of esds in distances, angles
and torsion angles; correlations between esds in cell parameters are only
used when they are defined by crystal symmetry. An approximate (isotropic)
treatment of cell esds is used for estimating esds involving l.s. planes.
;

loop_
_geom_bond_atom_site_label_1
_geom_bond_atom_site_label_2
_geom_bond_distance
_geom_bond_site_symmetry_2
_geom_bond_publ_flag
Cu2 O5 1.953(4) 98 ?
Cu2 O5 1.953(4) . ?
Cu2 O5 1.953(4) 109 ?
Cu2 O5 1.953(4) 14 ?
Cu2 O1 2.171(8) . ?
Cu2 Cu1 2.6445(15) . ?
Cu1 O3 1.952(4) 98 ?
Cu1 O3 1.952(4) . ?
Cu1 O3 1.952(4) 14 ?
Cu1 O3 1.952(4) 109 ?
Cu1 O2 2.142(9) . ?
Cu3 N1 1.967(5) 182 ?

Cu3 N1 1.967(5) . ?
Cu3 O6 1.978(13) . ?
Cu3 O12 2.012(4) . ?
Cu3 O7 2.20(2) . ?
Mo1 O8 1.677(9) . ?
Mo1 O11 1.908(2) . ?
Mo1 O11 1.908(2) 12 ?
Mo1 O9 1.924(5) 80 ?
Mo1 O9 1.924(5) . ?
Mo1 O10 2.335(8) . ?
O5 C1 1.260(6) . ?
O3 C1 1.258(6) . ?
N1 N1 1.278(9) 120 ?
N1 N2 1.317(6) . ?
C1 C3 1.493(7) . ?
N2 C6 1.315(7) . ?
O12 Cu3 2.012(4) 80 ?
O12 Cu3 2.012(4) 31_554 ?
C3 C2 1.383(6) . ?
C3 C4 1.384(7) . ?
O11 Mo1 1.908(2) 11 ?
O9 Mo1 1.924(5) 31_554 ?
O10 Si1 1.608(14) . ?
O10 Mo1 2.335(8) 80 ?
O10 Mo1 2.335(8) 31_554 ?
C4 C5 1.372(7) . ?
C5 C4 1.372(7) 120 ?
C5 C6 1.507(11) . ?
C2 C3 1.383(6) 120 ?
C6 N2 1.315(7) 120 ?
Si1 O10 1.608(14) 52_554 ?
Si1 O10 1.608(14) 27_554 ?
Si1 O10 1.608(14) 74 ?

loop_
 _geom_angle_atom_site_label_1
 _geom_angle_atom_site_label_2
 _geom_angle_atom_site_label_3
 _geom_angle
 _geom_angle_site_symmetry_1
 _geom_angle_site_symmetry_3
 _geom_angle_publ_flag
O5 Cu2 O5 89.7(2) 98 . ?
O5 Cu2 O5 168.1(2) 98 109 ?

O5 Cu2 O5 89.0(2) . 109 ?
O5 Cu2 O5 89.0(2) 98 14 ?
O5 Cu2 O5 168.1(2) . 14 ?
O5 Cu2 O5 89.7(2) 109 14 ?
O5 Cu2 O1 95.95(11) 98 . ?
O5 Cu2 O1 95.95(11) . . ?
O5 Cu2 O1 95.95(11) 109 . ?
O5 Cu2 O1 95.95(11) 14 . ?
O5 Cu2 Cu1 84.05(11) 98 . ?
O5 Cu2 Cu1 84.05(11) . . ?
O5 Cu2 Cu1 84.05(11) 109 . ?
O5 Cu2 Cu1 84.05(11) 14 . ?
O1 Cu2 Cu1 180.0(2) . . ?
O3 Cu1 O3 89.7(2) 98 . ?
O3 Cu1 O3 89.1(2) 98 14 ?
O3 Cu1 O3 168.1(2) . 14 ?
O3 Cu1 O3 168.1(2) 98 109 ?
O3 Cu1 O3 89.1(2) . 109 ?
O3 Cu1 O3 89.7(2) 14 109 ?
O3 Cu1 O2 95.95(11) 98 . ?
O3 Cu1 O2 95.95(11) . . ?
O3 Cu1 O2 95.95(11) 14 . ?
O3 Cu1 O2 95.95(11) 109 . ?
O3 Cu1 Cu2 84.05(11) 98 . ?
O3 Cu1 Cu2 84.05(11) . . ?
O3 Cu1 Cu2 84.05(11) 14 . ?
O3 Cu1 Cu2 84.05(11) 109 . ?
O2 Cu1 Cu2 180.0(2) . . ?
N1 Cu3 N1 174.9(3) 182 . ?
N1 Cu3 O6 91.60(15) 182 . ?
N1 Cu3 O6 91.60(15) . . ?
N1 Cu3 O12 87.63(14) 182 . ?
N1 Cu3 O12 87.63(15) . . ?
O6 Cu3 O12 150.9(9) . . ?
N1 Cu3 O7 91.6(2) 182 . ?
N1 Cu3 O7 91.6(2) . . ?
O6 Cu3 O7 102.1(10) . . ?
O12 Cu3 O7 107.0(9) . . ?
O8 Mo1 O11 102.2(4) . . ?
O8 Mo1 O11 102.2(4) . 12 ?
O11 Mo1 O11 87.3(5) . 12 ?
O8 Mo1 O9 99.7(4) . 80 ?
O11 Mo1 O9 158.1(4) . 80 ?
O11 Mo1 O9 87.6(4) 12 80 ?

O8 Mo1 O9 99.7(4) . . ?
O11 Mo1 O9 87.6(4) . . ?
O11 Mo1 O9 158.1(4) 12 . ?
O9 Mo1 O9 89.2(6) 80 . ?
O8 Mo1 O10 172.3(5) . . ?
O11 Mo1 O10 83.3(3) . . ?
O11 Mo1 O10 83.3(3) 12 . ?
O9 Mo1 O10 75.0(3) 80 . ?
O9 Mo1 O10 75.0(3) . . ?
C1 O5 Cu2 122.9(3) . . ?
C1 O3 Cu1 123.0(3) . . ?
N1 N1 N2 110.3(3) 120 . ?
N1 N1 Cu3 122.02(14) 120 . ?
N2 N1 Cu3 127.7(4) . . ?
O3 C1 O5 125.6(5) . . ?
O3 C1 C3 117.2(4) . . ?
O5 C1 C3 117.2(4) . . ?
C6 N2 N1 103.4(5) . . ?
Cu3 O12 Cu3 113.5(3) 80 31_554 ?
Cu3 O12 Cu3 113.5(3) 80 . ?
Cu3 O12 Cu3 113.5(3) 31_554 . ?
C2 C3 C4 118.9(5) . . ?
C2 C3 C1 120.4(5) . . ?
C4 C3 C1 120.6(4) . . ?
Mo1 O11 Mo1 151.0(5) . 11 ?
Mo1 O9 Mo1 119.4(5) 31_554 . ?
Si1 O10 Mo1 124.8(3) . 80 ?
Si1 O10 Mo1 124.8(3) . 31_554 ?
Mo1 O10 Mo1 90.7(4) 80 31_554 ?
Si1 O10 Mo1 124.8(3) . . ?
Mo1 O10 Mo1 90.7(4) 80 . ?
Mo1 O10 Mo1 90.7(4) 31_554 . ?
C5 C4 C3 120.3(5) . . ?
C4 C5 C4 120.3(7) . 120 ?
C4 C5 C6 119.8(4) . . ?
C4 C5 C6 119.8(4) 120 . ?
C3 C2 C3 121.0(7) 120 . ?
N2 C6 N2 112.7(7) 120 . ?
N2 C6 C5 123.6(4) 120 . ?
N2 C6 C5 123.6(4) . . ?
O10 Si1 O10 109.471(1) 52_554 27_554 ?
O10 Si1 O10 109.471(1) 52_554 . ?
O10 Si1 O10 109.471(1) 27_554 . ?
O10 Si1 O10 109.471(1) 52_554 74 ?

O10 Si1 O10 109.471(2) 27_554 74 ?
O10 Si1 O10 109.471(1) . 74 ?

_diffrn_measured_fraction_theta_max 0.995
_diffrn_reflns_theta_full 24.98
_diffrn_measured_fraction_theta_full 0.995
_refine_diff_density_max 0.695
_refine_diff_density_min -1.231
_refine_diff_density_rms 0.105
SQUEEZE RESULTS (APPEND TO CIF)
loop_
  _platon_squeeze_void_nr
  _platon_squeeze_void_average_x
  _platon_squeeze_void_average_y
  _platon_squeeze_void_average_z
  _platon_squeeze_void_volume
  _platon_squeeze_void_count_electrons
  1 -0.005 -0.010 -0.005 57934.1 2936.9
_platon_squeeze_details
; ?
;

```
data_hlju-3
_audit_creation_date              2015-12-07
_audit_creation_method
;
Olex2 1.2
(compiled 2015.09.30 svn.r3233 for OlexSys, GUI svn.r5103)
;
_publ_contact_author_address      ?
_publ_contact_author_email        ?
_publ_contact_author_name         ''
_publ_contact_author_phone        ?
_publ_section_references
;
Bourhis, L.J., Dolomanov, O.V., Gildea, R.J., Howard, J.A.K., Puschmann, H.
 (2013). in preparation.

Bourhis, L.J., Dolomanov, O.V., Gildea, R.J., Howard, J.A.K., Puschmann, H.
 (2015). Acta Cryst. A71, 59-75.

Dolomanov, O.V., Bourhis, L.J., Gildea, R.J, Howard, J.A.K. & Puschmann, H.
 (2009), J. Appl. Cryst. 42, 339-341.
;
_chemical_formula_moiety          '2(C54 H18 Cu12 N24 O41), O40 P W12'
_chemical_formula_sum             'C108 H36 Cu24 N48 O122 P W12'
_chemical_formula_weight          7720.10
loop_
  _atom_type_symbol
  _atom_type_scat_dispersion_real
  _atom_type_scat_dispersion_imag
  _atom_type_scat_Cromer_Mann_a1
  _atom_type_scat_Cromer_Mann_a2
  _atom_type_scat_Cromer_Mann_a3
  _atom_type_scat_Cromer_Mann_a4
  _atom_type_scat_Cromer_Mann_b1
  _atom_type_scat_Cromer_Mann_b2
  _atom_type_scat_Cromer_Mann_b3
  _atom_type_scat_Cromer_Mann_b4
  _atom_type_scat_Cromer_Mann_c
  _atom_type_scat_source
  _atom_type_scat_dispersion_source
 C 0.00347 0.00161 2.31000 1.02000 1.58860 0.86500 20.84390 10.20750 0.56870
 51.65120 0.215599998832
 'International Tables Volume C Table 6.1.1.4 (pp. 500-502)'
```

```
 'Henke, Gullikson and Davis, At. Data and Nucl. Data Tables, 1993, 54, 2'
 H 0.00000 0.00000 0.49300 0.32291 0.14019 0.04081 10.51090 26.12570 3.14236
 57.79970 0.0030380000826
 'International Tables Volume C Table 6.1.1.4 (pp. 500-502)'
 'Henke, Gullikson and Davis, At. Data and Nucl. Data Tables, 1993, 54, 2'
 O 0.01158 0.00611 3.04850 2.28680 1.54630 0.86700 13.27710 5.70110 0.32390
 32.90890 0.250800013542
 'International Tables Volume C Table 6.1.1.4 (pp. 500-502)'
 'Henke, Gullikson and Davis, At. Data and Nucl. Data Tables, 1993, 54, 2'
 N 0.00653 0.00323 12.21260 3.13220 2.01250 1.16630 0.00570 9.89330 28.99750
 0.58260 -11.5290002823
 'International Tables Volume C Table 6.1.1.4 (pp. 500-502)'
 'Henke, Gullikson and Davis, At. Data and Nucl. Data Tables, 1993, 54, 2'
 P 0.10434 0.09671 6.43450 4.17910 1.78000 1.49080 1.90670 27.15700 0.52600
 68.16450 1.11489999294
 'International Tables Volume C Table 6.1.1.4 (pp. 500-502)'
 'Henke, Gullikson and Davis, At. Data and Nucl. Data Tables, 1993, 54, 2'
 W -0.66836 6.76498 29.08180 15.43000 14.43270 5.11982 1.72029 9.22590 0.32170
 57.05600 9.88749980927
 'International Tables Volume C Table 6.1.1.4 (pp. 500-502)'
 'Henke, Gullikson and Davis, At. Data and Nucl. Data Tables, 1993, 54, 2'
 Cu 0.34125 1.28947 13.33800 7.16760 5.61580 1.67350 3.58280 0.24700 11.39660
 64.81260 1.19099998474
 'International Tables Volume C Table 6.1.1.4 (pp. 500-502)'
 'Henke, Gullikson and Davis, At. Data and Nucl. Data Tables, 1993, 54, 2'

_space_group_crystal_system            'cubic'
_space_group_IT_number                 225
_space_group_name_H-M_alt              'F m -3 m'
_space_group_name_Hall                 '-F 4 2 3'
loop_
   _space_group_symop_id
   _space_group_symop_operation_xyz
 1 x,y,z
 2 x,-z,y
 3 x,z,-y
 4 z,y,-x
 5 -z,y,x
 6 -y,x,z
 7 y,-x,z
 8 z,x,y
 9 y,z,x
 10 -y,-z,x
 11 z,-x,-y
```

12 -y,z,-x
13 -z,-x,y
14 -z,x,-y
15 y,-z,-x
16 x,-y,-z
17 -x,y,-z
18 -x,-y,z
19 y,x,-z
20 -y,-x,-z
21 z,-y,x
22 -z,-y,-x
23 -x,z,y
24 -x,-z,-y
25 -x,-y,-z
26 -x,z,-y
27 -x,-z,y
28 -z,-y,x
29 z,-y,-x
30 y,-x,-z
31 -y,x,-z
32 -z,-x,-y
33 -y,-z,-x
34 y,z,-x
35 -z,x,y
36 y,-z,x
37 z,x,-y
38 z,-x,y
39 -y,z,x
40 -x,y,z
41 x,-y,z
42 x,y,-z
43 -y,-x,z
44 y,x,z
45 -z,y,-x
46 z,y,x
47 x,-z,-y
48 x,z,y
49 x,y+1/2,z+1/2
50 x,-z+1/2,y+1/2
51 x,z+1/2,-y+1/2
52 z,y+1/2,-x+1/2
53 -z,y+1/2,x+1/2
54 -y,x+1/2,z+1/2
55 y,-x+1/2,z+1/2

56 z,x+1/2,y+1/2
57 y,z+1/2,x+1/2
58 -y,-z+1/2,x+1/2
59 z,-x+1/2,-y+1/2
60 -y,z+1/2,-x+1/2
61 -z,-x+1/2,y+1/2
62 -z,x+1/2,-y+1/2
63 y,-z+1/2,-x+1/2
64 x,-y+1/2,-z+1/2
65 -x,y+1/2,-z+1/2
66 -x,-y+1/2,z+1/2
67 y,x+1/2,-z+1/2
68 -y,-x+1/2,-z+1/2
69 z,-y+1/2,x+1/2
70 -z,-y+1/2,-x+1/2
71 -x,z+1/2,y+1/2
72 -x,-z+1/2,-y+1/2
73 -x,-y+1/2,-z+1/2
74 -x,z+1/2,-y+1/2
75 -x,-z+1/2,y+1/2
76 -z,-y+1/2,x+1/2
77 z,-y+1/2,-x+1/2
78 y,-x+1/2,-z+1/2
79 -y,x+1/2,-z+1/2
80 -z,-x+1/2,-y+1/2
81 -y,-z+1/2,-x+1/2
82 y,z+1/2,-x+1/2
83 -z,x+1/2,y+1/2
84 y,-z+1/2,x+1/2
85 z,x+1/2,-y+1/2
86 z,-x+1/2,y+1/2
87 -y,z+1/2,x+1/2
88 -x,y+1/2,z+1/2
89 x,-y+1/2,z+1/2
90 x,y+1/2,-z+1/2
91 -y,-x+1/2,z+1/2
92 y,x+1/2,z+1/2
93 -z,y+1/2,-x+1/2
94 z,y+1/2,x+1/2
95 x,-z+1/2,-y+1/2
96 x,z+1/2,y+1/2
97 x+1/2,y,z+1/2
98 x+1/2,-z,y+1/2
99 x+1/2,z,-y+1/2

100 z+1/2,y,-x+1/2
101 -z+1/2,y,x+1/2
102 -y+1/2,x,z+1/2
103 y+1/2,-x,z+1/2
104 z+1/2,x,y+1/2
105 y+1/2,z,x+1/2
106 -y+1/2,-z,x+1/2
107 z+1/2,-x,-y+1/2
108 -y+1/2,z,-x+1/2
109 -z+1/2,-x,y+1/2
110 -z+1/2,x,-y+1/2
111 y+1/2,-z,-x+1/2
112 x+1/2,-y,-z+1/2
113 -x+1/2,y,-z+1/2
114 -x+1/2,-y,z+1/2
115 y+1/2,x,-z+1/2
116 -y+1/2,-x,-z+1/2
117 z+1/2,-y,x+1/2
118 -z+1/2,-y,-x+1/2
119 -x+1/2,z,y+1/2
120 -x+1/2,-z,-y+1/2
121 -x+1/2,-y,-z+1/2
122 -x+1/2,z,-y+1/2
123 -x+1/2,-z,y+1/2
124 -z+1/2,-y,x+1/2
125 z+1/2,-y,-x+1/2
126 y+1/2,-x,-z+1/2
127 -y+1/2,x,-z+1/2
128 -z+1/2,-x,-y+1/2
129 -y+1/2,-z,-x+1/2
130 y+1/2,z,-x+1/2
131 -z+1/2,x,y+1/2
132 y+1/2,-z,x+1/2
133 z+1/2,x,-y+1/2
134 z+1/2,-x,y+1/2
135 -y+1/2,z,x+1/2
136 -x+1/2,y,z+1/2
137 x+1/2,-y,z+1/2
138 x+1/2,y,-z+1/2
139 -y+1/2,-x,z+1/2
140 y+1/2,x,z+1/2
141 -z+1/2,y,-x+1/2
142 z+1/2,y,x+1/2
143 x+1/2,-z,-y+1/2

144 x+1/2,z,y+1/2
145 x+1/2,y+1/2,z
146 x+1/2,-z+1/2,y
147 x+1/2,z+1/2,-y
148 z+1/2,y+1/2,-x
149 -z+1/2,y+1/2,x
150 -y+1/2,x+1/2,z
151 y+1/2,-x+1/2,z
152 z+1/2,x+1/2,y
153 y+1/2,z+1/2,x
154 -y+1/2,-z+1/2,x
155 z+1/2,-x+1/2,-y
156 -y+1/2,z+1/2,-x
157 -z+1/2,-x+1/2,y
158 -z+1/2,x+1/2,-y
159 y+1/2,-z+1/2,-x
160 x+1/2,-y+1/2,-z
161 -x+1/2,y+1/2,-z
162 -x+1/2,-y+1/2,z
163 y+1/2,x+1/2,-z
164 -y+1/2,-x+1/2,-z
165 z+1/2,-y+1/2,x
166 -z+1/2,-y+1/2,-x
167 -x+1/2,z+1/2,y
168 -x+1/2,-z+1/2,-y
169 -x+1/2,-y+1/2,-z
170 -x+1/2,z+1/2,-y
171 -x+1/2,-z+1/2,y
172 -z+1/2,-y+1/2,x
173 z+1/2,-y+1/2,-x
174 y+1/2,-x+1/2,-z
175 -y+1/2,x+1/2,-z
176 -z+1/2,-x+1/2,-y
177 -y+1/2,-z+1/2,-x
178 y+1/2,z+1/2,-x
179 -z+1/2,x+1/2,y
180 y+1/2,-z+1/2,x
181 z+1/2,x+1/2,-y
182 z+1/2,-x+1/2,y
183 -y+1/2,z+1/2,x
184 -x+1/2,y+1/2,z
185 x+1/2,-y+1/2,z
186 x+1/2,y+1/2,-z
187 -y+1/2,-x+1/2,z

188 y+1/2,x+1/2,z
189 -z+1/2,y+1/2,-x
190 z+1/2,y+1/2,x
191 x+1/2,-z+1/2,-y
192 x+1/2,z+1/2,y

_symmetry_Int_Tables_number 225
_cell_length_a 44.4742(7)
_cell_length_b 44.4742(7)
_cell_length_c 44.4742(7)
_cell_angle_alpha 90
_cell_angle_beta 90
_cell_angle_gamma 90
_cell_volume 87968(4)
_cell_formula_units_Z 8
_cell_measurement_temperature 293(2)
_cell_measurement_reflns_used 3470
_cell_measurement_theta_max 28.3412
_cell_measurement_theta_min 3.0321
_exptl_absorpt_coefficient_mu 4.312
_exptl_absorpt_correction_T_max 0.439
_exptl_absorpt_correction_T_min 0.422
_exptl_absorpt_correction_type multi-scan
_exptl_absorpt_process_details
;
CrysAlisPro, Agilent Technologies,
Version 1.171.35.21 (release 20-01-2012 CrysAlis171 .NET)
(compiled Jan 23 2012,18:06:46)
Empirical absorption correction using spherical harmonics,
implemented in SCALE3 ABSPACK scaling algorithm.
;
_exptl_crystal_description polyhedra
_exptl_crystal_colour blue
_exptl_crystal_size_max 0.20
_exptl_crystal_size_mid 0.20
_exptl_crystal_size_min 0.20
_exptl_crystal_density_diffrn 1.1657
_exptl_crystal_F_000 28760.0
_diffrn_reflns_av_R_equivalents 0.1236
_diffrn_reflns_av_unetI/netI 0.1372
_diffrn_reflns_limit_h_max 35
_diffrn_reflns_limit_h_min -26
_diffrn_reflns_limit_k_max 56
_diffrn_reflns_limit_k_min -14

```
_diffrn_reflns_limit_l_max              58
_diffrn_reflns_limit_l_min              -21
_diffrn_reflns_number                   15788
_diffrn_reflns_theta_full               24.9930
_diffrn_reflns_theta_max                24.99
_diffrn_reflns_theta_min                3.04
_diffrn_detector_area_resol_mean    16.1954
_diffrn_measured_fraction_theta_full 0.9751
_diffrn_measured_fraction_theta_max 0.9751
_diffrn_measurement_details
;
#__ type_ start__ end____ width___ exp.time_
  1 omega     41.00     84.50     0.7500   500.0000
omega____ theta____ kappa____ phi______ frames
    -            23.0530 -122.0000    -35.0000 58
;
_diffrn_measurement_device_type      'multiwire proportional'
_diffrn_measurement_method             'phi and omega scans'
_diffrn_orient_matrix_UB_11            0.0103026000
_diffrn_orient_matrix_UB_12            0.0096500000
_diffrn_orient_matrix_UB_13            0.0074185000
_diffrn_orient_matrix_UB_21            0.0019344000
_diffrn_orient_matrix_UB_22            -0.0109049000
_diffrn_orient_matrix_UB_23            0.0114714000
_diffrn_orient_matrix_UB_31            0.0120182000
_diffrn_orient_matrix_UB_32            -0.0065319000
_diffrn_orient_matrix_UB_33            -0.0082115000
_diffrn_radiation_monochromator      graphite
_diffrn_ambient_temperature            293(2)
_diffrn_radiation_type                 'Mo K\a'
_diffrn_radiation_wavelength           0.71073
_diffrn_source                         'Enhance (Mo) X-ray Source'
_reflns_Friedel_coverage               0.0
_reflns_limit_h_max                    30
_reflns_limit_h_min                    0
_reflns_limit_k_max                    52
_reflns_limit_k_min                    5
_reflns_limit_l_max                    37
_reflns_limit_l_min                    0
_reflns_number_gt                      1872
_reflns_number_total                   3777
_reflns_odcompleteness_completeness 99.40
_reflns_odcompleteness_iscentric    1
_reflns_odcompleteness_theta           26.32
```

_reflns_threshold_expression I>=2u(I)
_computing_cell_refinement
;
CrysAlisPro, Agilent Technologies,
Version 1.171.35.21 (release 20-01-2012 CrysAlis171 .NET)
(compiled Jan 23 2012,18:06:46)
;
_computing_data_collection
;
CrysAlisPro, Agilent Technologies,
Version 1.171.35.21 (release 20-01-2012 CrysAlis171 .NET)
(compiled Jan 23 2012,18:06:46)
;
_computing_data_reduction
;
CrysAlisPro, Agilent Technologies,
Version 1.171.35.21 (release 20-01-2012 CrysAlis171 .NET)
(compiled Jan 23 2012,18:06:46)
;
_computing_molecular_graphics 'Olex2 (Dolomanov et al., 2009)'
_computing_publication_material 'Olex2 (Dolomanov et al., 2009)'
_computing_structure_refinement 'olex2.refine (Bourhis et al., 2015)'
_computing_structure_solution 'olex2.solve (Bourhis et al., 2015)'
_refine_diff_density_max 2.1956
_refine_diff_density_min -1.1318
_refine_diff_density_rms 0.1541
_refine_ls_d_res_high 0.8411
_refine_ls_d_res_low 6.7047
_refine_ls_goodness_of_fit_ref 0.8531
_refine_ls_hydrogen_treatment mixed
_refine_ls_matrix_type full
_refine_ls_number_constraints 4
_refine_ls_number_parameters 79
_refine_ls_number_reflns 3777
_refine_ls_number_restraints 7
_refine_ls_R_factor_all 0.1302
_refine_ls_R_factor_gt 0.0994
_refine_ls_restrained_S_all 0.8522
_refine_ls_shift/su_max 0.0002
_refine_ls_shift/su_mean 0.0000
_refine_ls_structure_factor_coef Fsqd
_refine_ls_weighting_details
'w=1/[\s^2^(Fo^2^)+(0.1587P)^2^] where P=(Fo^2^+2Fc^2^)/3'
_refine_ls_weighting_scheme calc

_refine_ls_wR_factor_gt 0.2314
_refine_ls_wR_factor_ref 0.2547
_olex2_refinement_description
;
1. Fixed Uiso
 At 1.2 times of:
  All C(H) groups
2. Restrained distances
 P1-O96
 1.4 with sigma of 0.02
 W1-O77
 1.835 with sigma of 0.02
 O78-W1
 2.247 with sigma of 0.02
 W1-O79
 1.957 with sigma of 0.02
 O78-O79
 3.225 with sigma of 0.04
 O79-O77
 2.922 with sigma of 0.04
 O77-O78
 3.286 with sigma of 0.04
3. Rigid bond restraints
 with sigma for 1-2 distances of 0.01 and sigma for 1-3 distances of 0.01
4. Uiso/Uaniso restraints and constraints
: within 3.8A with sigma of 0.04 and sigma for terminal atoms of 0.08
5. Others
 Fixed Sof: O2(0.5) P1(0.04167)
6.a Aromatic/amide H refined with riding coordinates:
 C3(H3), C8(H8)
;
loop_
 _atom_site_label
 _atom_site_type_symbol
 _atom_site_fract_x
 _atom_site_fract_y
 _atom_site_fract_z
 _atom_site_U_iso_or_equiv
 _atom_site_adp_type
 _atom_site_occupancy
 _atom_site_site_symmetry_order
 _atom_site_refinement_flags_posn
 _atom_site_refinement_flags_adp
 _atom_site_refinement_flags_occupancy

Cu1 Cu 0.168668(17) 0.168668(17) 0.0 0.0269(4) Uani 1.000000 4 S T P
Cu2 Cu 0.126563(18) 0.126563(18) 0.0 0.0278(4) Uani 1.000000 4 S T P
Cu3 Cu 0.32504(5) 0.17496(5) 0.12195(6) 0.1786(17) Uani 1.000000 2 S T P
O2 O 0.0928(3) 0.0928(3) 0.0 0.320(9) Uiso 2.000000 4 S T P
O3 O 0.20274(16) 0.20274(16) 0.0 0.065(3) Uiso 1.000000 4 S T P
O4 O 0.15180(9) 0.10747(9) 0.03080(9) 0.0444(10) Uiso 1.000000 1 . . .
O5 O 0.18716(10) 0.14352(10) 0.03059(10) 0.0482(10) Uiso 1.000000 1 . . .
O11 O 0.3393(2) 0.1607(2) 0.1607(2) 0.091(4) Uiso 1.000020 6 S T P
O30 O 0.3042(3) 0.1958(3) 0.0891(5) 0.236(8) Uiso 1.000000 2 S T P
N3 N 0.27094(17) 0.13891(19) 0.10352(18) 0.091(2) Uiso 1.000000 1 . . .
N4 N 0.29256(17) 0.14567(16) 0.12575(16) 0.085(2) Uiso 1.000000 1 . . .
C1 C 0.2560(3) 0.11874(18) 0.11874(18) 0.070(3) Uiso 1.000000 2 S T P
C2 C 0.2281(2) 0.10390(17) 0.10390(17) 0.065(3) Uiso 1.000000 2 S T P
C3 C 0.21645(15) 0.11625(16) 0.07728(16) 0.0553(17) Uiso 1.000000 1 . . .
H3 H 0.22654(15) 0.13161(16) 0.06731(16) 0.066(2) Uiso 1.000000 1 R . .
C7 C 0.19021(13) 0.10528(12) 0.06648(12) 0.0372(13) Uiso 1.000000 1 . . .
C8 C 0.17745(18) 0.08043(13) 0.08043(13) 0.0407(18) Uiso 1.000000 2 S T P
H8 H 0.15955(18) 0.07278(13) 0.07278(13) 0.049(2) Uiso 1.000000 2 RS T P
C10 C 0.17482(14) 0.12091(13) 0.04004(14) 0.0421(14) Uiso 1.000000 1 . . .
W1 W 0.30507(8) 0.19493(8) 0.24721(11) 0.482(3) Uani 1.000000 2 DS T P
O96 O 0.2638(4) 0.2638(4) 0.2638(4) 0.193(12) Uiso 1.000020 6 DS T P
P1 P 0.2334(4) 0.2666(4) 0.2666(4) 0.135(12) Uiso 0.250020 6 DS T P
O79 O 0.2813(3) 0.1698(4) 0.2187(3) 0.184(7) Uiso 1.000000 2 DS T P
O78 O 0.3394(3) 0.1606(3) 0.2618(8) 0.41(2) Uiso 1.000000 2 DS T P
O77 O 0.2780(3) 0.1976(6) 0.2780(3) 0.278(13) Uiso 1.000000 2 DS T P

loop_
_atom_site_aniso_label
_atom_site_aniso_U_11
_atom_site_aniso_U_22
_atom_site_aniso_U_33
_atom_site_aniso_U_12
_atom_site_aniso_U_13
_atom_site_aniso_U_23
Cu1 0.0301(5) 0.0301(5) 0.0205(6) -0.0110(5) -0.000000 0.000000
Cu2 0.0310(5) 0.0310(5) 0.0214(6) -0.0093(5) -0.000000 0.000000
Cu3 0.1770(18) 0.1770(18) 0.182(2) -0.1542(19) -0.1437(18) 0.1437(18)
W1 0.487(4) 0.487(4) 0.473(6) 0.011(5) -0.001(3) 0.001(3)

loop_
_geom_bond_atom_site_label_1
_geom_bond_atom_site_label_2
_geom_bond_distance
_geom_bond_site_symmetry_2

_geom_bond_publ_flag
Cu1 Cu2 2.6482(16) . ?
Cu1 O3 2.143(10) . ?
Cu1 O5 1.944(4) . ?
Cu1 O5 1.944(4) 19 ?
Cu1 O5 1.944(4) 42 ?
Cu1 O5 1.944(4) 44 ?
Cu2 O2 2.12(2) . ?
Cu2 O4 1.964(4) 19 ?
Cu2 O4 1.964(4) 44 ?
Cu2 O4 1.964(4) . ?
Cu2 O4 1.964(4) 42 ?
Cu3 O11 1.943(3) . ?
Cu3 O30 1.96(2) . ?
Cu3 N4 1.953(7) 187 ?
Cu3 N4 1.953(7) . ?
O4 C10 1.255(7) . ?
O5 C10 1.220(7) . ?
N3 N4 1.412(9) . ?
N3 C1 1.304(9) . ?
N4 N4 1.253(14) 48 ?
C1 C2 1.555(15) . ?
C2 C3 1.404(8) . ?
C2 C3 1.404(8) 48 ?
C3 H3 0.9300 . ?
C3 C7 1.353(8) . ?
C7 C8 1.389(7) . ?
C7 C10 1.528(8) . ?
C8 H8 0.9300 . ?
W1 P1 2.569(19) 77 ?
W1 O79 1.994(9) 108 ?
W1 O79 1.994(9) 141 ?
W1 O78 2.258(16) . ?
W1 O77 1.827(5) 46 ?
W1 O77 1.827(5) 187 ?
O96 O96 1.74(4) 110 ?
O96 O96 1.74(5) 154 ?
O96 O96 1.74(4) 59 ?
O96 P1 1.363(16) 157 ?
O96 P1 1.363(16) 9 ?
O96 P1 1.363(16) 162 ?
P1 P1 2.09(5) 59 ?
P1 P1 2.09(5) 63 ?
P1 P1 2.09(5) 64 ?

P1 O77 1.75(4) 110 ?
P1 O77 1.75(4) 162 ?
P1 O77 1.75(4) 9 ?
O77 O77 1.54(3) 59 ?
O77 O77 1.54(4) 154 ?

loop_
_geom_angle_atom_site_label_1
_geom_angle_atom_site_label_2
_geom_angle_atom_site_label_3
_geom_angle
_geom_angle_site_symmetry_1
_geom_angle_site_symmetry_3
_geom_angle_publ_flag
O3 Cu1 Cu2 180.0 . . ?
O5 Cu1 Cu2 83.82(12) 19 . ?
O5 Cu1 Cu2 83.82(12) 44 . ?
O5 Cu1 Cu2 83.82(12) . . ?
O5 Cu1 Cu2 83.82(12) 42 . ?
O5 Cu1 O3 96.18(12) 42 . ?
O5 Cu1 O3 96.18(12) 44 . ?
O5 Cu1 O3 96.18(12) . . ?
O5 Cu1 O3 96.18(12) 19 . ?
O5 Cu1 O5 89.8(3) 44 . ?
O5 Cu1 O5 167.6(2) 19 . ?
O5 Cu1 O5 88.8(3) 42 . ?
O5 Cu1 O5 88.8(3) 44 19 ?
O5 Cu1 O5 89.8(3) 42 19 ?
O5 Cu1 O5 167.6(2) 44 42 ?
O2 Cu2 Cu1 180.0 . . ?
O4 Cu2 Cu1 84.36(12) 42 . ?
O4 Cu2 Cu1 84.36(12) 44 . ?
O4 Cu2 Cu1 84.36(12) . . ?
O4 Cu2 Cu1 84.36(12) 19 . ?
O4 Cu2 O2 95.64(12) . . ?
O4 Cu2 O2 95.64(12) 19 . ?
O4 Cu2 O2 95.64(12) 44 . ?
O4 Cu2 O2 95.64(12) 42 . ?
O4 Cu2 O4 168.7(2) 44 42 ?
O4 Cu2 O4 90.4(2) 42 19 ?
O4 Cu2 O4 88.5(2) 44 19 ?
O4 Cu2 O4 88.5(2) 42 . ?
O4 Cu2 O4 90.4(2) 44 . ?
O4 Cu2 O4 168.7(2) 19 . ?

O30 Cu3 O11 165.6(8) . . ?
N4 Cu3 O11 87.0(2) 187 . ?
N4 Cu3 O11 87.0(2) . . ?
N4 Cu3 O30 91.7(2) 187 . ?
N4 Cu3 O30 91.7(2) . . ?
N4 Cu3 N4 168.5(5) 187 . ?
C10 O4 Cu2 119.2(4) . . ?
C10 O5 Cu1 121.6(4) . . ?
Cu3 O11 Cu3 118.2(2) 141 48 ?
Cu3 O11 Cu3 118.2(2) 141 . ?
Cu3 O11 Cu3 118.2(2) 48 . ?
C1 N3 N4 97.4(8) . . ?
N3 N4 Cu3 125.8(5) . . ?
N4 N4 Cu3 122.2(2) 48 . ?
N4 N4 N3 110.2(5) 48 . ?
N3 C1 N3 117.1(11) 48 . ?
C2 C1 N3 118.5(6) . 48 ?
C2 C1 N3 118.5(6) . . ?
C3 C2 C1 119.1(5) . . ?
C3 C2 C1 119.1(5) 48 . ?
C3 C2 C3 121.6(10) 48 . ?
H3 C3 C2 120.8(5) . . ?
C7 C3 C2 118.4(7) . . ?
C7 C3 H3 120.8(4) . . ?
C8 C7 C3 118.7(6) . . ?
C10 C7 C3 119.7(5) . . ?
C10 C7 C8 121.5(5) . . ?
C7 C8 C7 122.9(7) 48 . ?
H8 C8 C7 118.5(4) . . ?
H8 C8 C7 118.5(4) . 48 ?
O5 C10 O4 130.3(6) . . ?
C7 C10 O4 113.6(5) . . ?
C7 C10 O5 116.0(5) . . ?
O79 W1 P1 103.5(6) 108 77 ?
O79 W1 P1 103.5(6) 141 77 ?
O79 W1 O79 101.0(9) 141 108 ?
O78 W1 P1 143.6(11) . 77 ?
O78 W1 O79 99.3(7) . 108 ?
O78 W1 O79 99.3(7) . 141 ?
O77 W1 P1 42.9(9) 187 77 ?
O77 W1 P1 42.9(9) 46 77 ?
O77 W1 O79 144.3(10) 187 141 ?
O77 W1 O79 99.4(7) 46 141 ?
O77 W1 O79 99.4(7) 187 108 ?

O77 W1 O79 144.3(10) 46 108 ?
O77 W1 O78 105.9(11) 46 . ?
O77 W1 O78 105.9(11) 187 . ?
O77 W1 O77 49.7(13) 46 187 ?
O96 O96 O96 60.0(8) 59 110 ?
P1 O96 O96 50.4(15) 157 110 ?
P1 O96 O96 50.4(13) 162 59 ?
P1 O96 O96 50.4(15) 9 59 ?
P1 O96 O96 97.4(18) 157 59 ?
P1 O96 O96 97(2) 162 110 ?
P1 O96 O96 50.4(13) 9 110 ?
P1 O96 P1 100(2) 162 9 ?
P1 O96 P1 100(2) 157 9 ?
P1 O96 P1 100(2) 157 162 ?
W1 P1 W1 90.2(8) 9 172 ?
W1 P1 W1 90.2(8) 44 172 ?
W1 P1 W1 90.2(8) 44 9 ?
O96 P1 W1 133.68(18) 108 9 ?
O96 P1 W1 133.68(18) 154 44 ?
O96 P1 W1 77.8(14) 154 9 ?
O96 P1 W1 133.68(18) 8 9 ?
O96 P1 W1 133.68(18) 108 172 ?
O96 P1 W1 133.68(18) 154 172 ?
O96 P1 W1 77.8(14) 108 44 ?
O96 P1 W1 77.8(14) 8 172 ?
O96 P1 W1 133.68(18) 8 44 ?
O96 P1 O96 79(3) 108 8 ?
O96 P1 O96 79(3) 154 8 ?
O96 P1 O96 79(3) 108 154 ?
P1 P1 W1 160.4(6) 64 172 ?
P1 P1 W1 160.4(6) 59 9 ?
P1 P1 W1 103.5 63 9 ?
P1 P1 W1 103.5(7) 64 9 ?
P1 P1 W1 103.5(5) 63 172 ?
P1 P1 W1 103.5(5) 64 44 ?
P1 P1 W1 160.4(6) 63 44 ?
P1 P1 W1 103.5(7) 59 172 ?
P1 P1 W1 103.5(2) 59 44 ?
P1 P1 O96 40.1(12) 59 8 ?
P1 P1 O96 82.6(18) 63 108 ?
P1 P1 O96 40.1(13) 63 8 ?
P1 P1 O96 82.6(18) 59 154 ?
P1 P1 O96 40.1(12) 64 154 ?
P1 P1 O96 40.1(12) 59 108 ?

P1 P1 O96 40.1(11) 63 154 ?
P1 P1 O96 40.1(10) 64 108 ?
P1 P1 O96 82.6(18) 64 8 ?
P1 P1 P1 60.0(7) 63 59 ?
O77 P1 W1 45.3(4) 110 172 ?
O77 P1 W1 45.3(4) 9 9 ?
O77 P1 W1 45.3(4) 162 172 ?
O77 P1 W1 45.3(4) 110 9 ?
O77 P1 W1 45.3(4) 9 44 ?
O77 P1 W1 85.3(12) 110 44 ?
O77 P1 W1 45.3(4) 162 44 ?
O77 P1 W1 85.3(12) 9 172 ?
O77 P1 W1 85.3(12) 162 9 ?
O77 P1 O96 113.4(14) 110 154 ?
O77 P1 O96 113.4(14) 9 108 ?
O77 P1 O96 163(2) 162 154 ?
O77 P1 O96 163(2) 110 108 ?
O77 P1 O96 113.4(14) 162 8 ?
O77 P1 O96 163(2) 9 8 ?
O77 P1 O96 113.4(14) 110 8 ?
O77 P1 O96 113.4(14) 9 154 ?
O77 P1 O96 113.4(14) 162 108 ?
O77 P1 P1 114.3(10) 110 63 ?
O77 P1 P1 148.2(10) 162 63 ?
O77 P1 P1 148.2(6) 110 59 ?
O77 P1 P1 148.2 9 59 ?
O77 P1 P1 148.2 9 63 ?
O77 P1 P1 114.3(10) 162 59 ?
O77 P1 O77 52.1(11) 162 110 ?
O77 P1 O77 52.1(13) 9 110 ?
O77 P1 O77 52.1(13) 162 9 ?
W1 O79 W1 111.1(8) . 157 ?
W1 O77 W1 169.6(13) 59 187 ?
P1 O77 W1 91.8(8) 63 187 ?
P1 O77 W1 91.8(8) 63 59 ?
O77 O77 W1 125.1(10) 59 187 ?
O77 O77 W1 125.1(8) 154 59 ?
O77 O77 W1 65.1(9) 154 187 ?
O77 O77 W1 65.1(9) 59 59 ?
O77 O77 P1 63.9(8) 59 63 ?
O77 O77 P1 63.9(7) 154 63 ?

_olex2_submission_special_instructions 'No special instructions were received'
loop_

```
    _restr_distance_atom_site_label_1
    _restr_distance_atom_site_label_2
    _restr_distance_site_symmetry_2
    _restr_distance_target
    _restr_distance_target_weight_param
    _restr_distance_diff
  W1 O79 1 1.9570 0.0200 -0.0371
  W1 O77 1 1.8350 0.0200 0.0079
  P1 O96 1 1.4000 0.0200 0.0373
  O79 O77 1 2.9220 0.0400 0.0059
  O78 W1 1 2.2470 0.0200 -0.0106
  O78 O79 1 3.2250 0.0400 -0.0208
  O77 O78 1 3.2860 0.0400 0.0165
# SQUEEZE RESULTS (APPEND TO CIF)
loop_
    _platon_squeeze_void_nr
    _platon_squeeze_void_average_x
    _platon_squeeze_void_average_y
    _platon_squeeze_void_average_z
    _platon_squeeze_void_volume
    _platon_squeeze_void_count_electrons
             1      -0.005      -0.011      -0.005     58331.8      -723.3
_platon_squeeze_details
; ?
;
```
